# Tocotrienol-Rich Fraction (TRF) Treatment Promotes Proliferation Capacity of Stress-Induced Premature Senescence Myoblasts and Modulates the Renewal of Satellite Cells: Microarray Analysis

**DOI:** 10.1155/2019/9141343

**Published:** 2019-01-10

**Authors:** Jing Jye Lim, Wan Ngah Wan Zurinah, Vincent Mouly, Abdul Karim Norwahidah

**Affiliations:** ^1^Department of Biochemistry, Faculty of Medicine, Level 17, Preclinical Building, Universiti Kebangsaan Malaysia Medical Centre, Jalan Yaacob Latif, Bandar Tun Razak, 56000 Cheras, Kuala Lumpur, Malaysia; ^2^Thérapie des Maladies du Muscle Strié Institut de Myologie, UM76-UPMC Univ. Paris 6/ U974-Inserm/UMR7215-CNRS, G.H. Pitié-Salpétrière-INSERM, UMRS 974, Institut de Myologie, Université Pierre et Marie Curie, Paris, France

## Abstract

Human skeletal muscle is a vital organ involved in movement and force generation. It suffers from deterioration in mass, strength, and regenerative capacity in sarcopenia. Skeletal muscle satellite cells are involved in the regeneration process in response to muscle loss. Tocotrienol, an isomer of vitamin E, was reported to have a protective effect on cellular aging. This research is aimed at determining the modulation of tocotrienol-rich fraction (TRF) on the gene expressions of stress-induced premature senescence (SIPS) human skeletal muscle myoblasts (CHQ5B). CHQ5B cells were divided into three groups, i.e., untreated young control, SIPS control (treated with 1 mM hydrogen peroxide), and TRF-posttreated groups (24 hours of 50 *μ*g/mL TRF treatment after SIPS induction). The differential gene expressions were assessed using microarray, GSEA, and KEGG pathway analysis. Results showed that TRF treatment significantly regulated the gene expressions, i.e., p53 (RRM2B, SESN1), ErbB (EREG, SHC1, and SHC3), and FoxO (MSTN, SMAD3) signalling pathways in the SIPS myoblasts compared to the SIPS control group (*p* < 0.05). TRF treatment modulated the proliferation capacity of SIPS myoblasts through regulation of ErbB (upregulation of expression of *EREG*, *SHC1*, and *SHC3*) and FoxO (downregulation of expression of *MSTN* and *SMAD3*) and maintaining the renewal of satellite cells through p53 signalling (upregulation of *RRM2B* and *SESN1*), MRF, cell cycle, and Wnt signalling pathways.

## 1. Introduction

Skeletal muscle is one of the largest organs in the body and contributed to 45-55% of the total body weight [1]. However, skeletal muscle cells are not able to self-replenish as it is a terminally differentiated cells. Thus, skeletal muscle cells require a population of resident adult stem cells, satellite cells for maintenance and repair [2]. Satellite cells are typically mitotically quiescent in resting muscle and will be activated to prepare for cell cycle entry upon stimulation and during muscle injury. A subset of satellite cells will be self-renewed to maintain the satellite cell pool and regenerate a rapidly proliferating transit-amplifying myoblast population.

Regeneration of skeletal muscle cells deteriorated with the aging process. This muscle degeneration process due to aging is termed sarcopenia [3]. Muscle degeneration started with 0.5–1% after 30 years of age and the rate increases as age reaches 65 years [4]. The mechanism of this degeneration is still yet to be deciphered clearly, but it can involve oxidative stress [5]. Muscle atrophy is reported to be stimulated by the PI3K/Akt and NF-*κ*B signalling pathways [6]. Activation of PI3K/Akt signalling regulates the skeletal muscle mass and metabolism in skeletal muscle [7]. On the other hand, inhibition of this signalling pathway would inhibit the FoxO protein and upregulate the atrophy-related gene (atrogin), such as *atrogin1/MAFbx1* and *MuRF* [7, 8]. Braun and Gautel proposed that NF-*κ*B also would involve in the regulation of atrogin and regulate the catabolism and anabolism of muscle protein [7].

Myostatin, a member of the TGF superfamily, was reported as a muscle atrophy inducer by activating the transcription factor SMAD3 which leads to atrogin-1 expression and inhibition of Akt/mTOR signalling and protein synthesis. Moreover, SMAD3 is also involved in the inhibition of peroxisome proliferator-activated receptor-coactivator-1 (PGC1) promoter activity and increased FoxO-mediated signalling [9]. Besides the mentioned FoxO signalling pathway and the expression of atrogin in muscle atrophy, Brack et al. suggested a different signalling pathway in muscle aging, which is the activation of the Wnt/*β*-catenin signalling pathway that leads to increased muscle fibrosis [10].

Cell senescence is categorised into three major mechanisms, i.e., replicative senescence, oncogene-induced senescence, and stress-induced premature senescence (SIPS) [11]. SIPS cells and replicative senescence cells are similar in molecular action and morphology, such as flat and larger cells, increased activities of senescence-associated *β*-galactosidase, and cell cycle arrest. However, SIPS was not affected by the length of telomere [12–14]. Hydrogen peroxide (H_2_O_2_) is a common stress mediator in the SIPS model as it induced the cells to have senescence morphology alike with the replicative senescence cells [11, 12]. Generation of H_2_O_2_ by the mitochondria or external sources from the cells leads to the damage of mitochondrial components and thus initiated the degeneration process [15].

Since the degeneration of skeletal muscle was closely related to oxidative stress, it was suggested that reestablishment of the redox balance would be beneficial in the amelioration of age-related degeneration in skeletal muscle [16]. Vitamin E, especially tocotrienol, has been widely reported for its antioxidant effects in preventing aging [17–19]. Supplementation of vitamin E (*α*-tocopherol) together with vitamin C reduced oxidative stress and increased the activities of antioxidant enzymes in the rat skeletal muscle [20].

Various approaches, including pharmacological interventions, nutrition, and physical activities, have been reported to improve the aging conditions of the skeletal muscle. However, to date there is neither supplementation nor intervention that works effectively with minimum side effects [3]. Howard et al. reported that tocopherol promoted the repair of plasma membrane in mouse myocytes [21]. However, these findings were focused on the effects of tocopherol, but not tocotrienol, on skeletal muscle. Previously, our study revealed that the tocotrienol-rich fraction (TRF) has reduced the senescence morphology and improved the proliferation capacity towards stress-induced premature senescence cells [22]. Hence, we would like to identify and elucidate the mechanism involved in the regenerative action of TRF on stress-induced premature skeletal muscle cells.

## 2. Materials and Methods

### 2.1. CHQ5B Cell Culture

Human satellite cells were isolated from a biopsy of a 5-day-old infant quadriceps muscle and were kindly provided by Dr. Vincent Mouly from UMRS 787, Institut de Myologie, INSERM, Université Pierre et Marie Curie, Paris, France. Upon isolation, the satellite cells proliferated in culture as myoblasts (known as CHQ5B cells) and were considered to be at 1 mean population doubling (MPD). CHQ5B cells were cultivated in a growth medium at 37°C in a humid atmosphere containing 5% carbon dioxide (CO_2_) as described previously [22].

### 2.2. Treatment Protocols

The Tocotrienol-rich fraction (TRF) was purchased from Sime Darby Sdn. Bhd., Selangor, Malaysia (TRF Gold Tri E 70). TRF consists of *α*-tocotrienol (26.67%), *β*-tocotrienol (4.29%), *γ*-tocotrienol (32.60%), *δ*-tocotrienol (15.53%), and *α*-tocopherol (20.81%) [22]. The CHQ5B cells (PD 29 ± 3) were incubated with different treatments, i.e., untreated young control, stress-induced premature senescence (SIPS), and TRF-treated group (SIPS myoblasts posttreated with TRF). Untreated young control cells were cultured without any treatment with ROS inducer or TRF. The SIPS model was established by exposure of CHQ5B cells to the stressor, 1 mM H_2_O_2_ diluted in growth medium for 30 minutes [22, 23]. In the TRF-treated group, the CHQ5B cells were exposed to the stressor at 1 mM H_2_O_2_ for 30 minutes followed by incubation in 50 *μ*g/mL TRF (Sime Darby Bioganic Sdn Bhd) for 24 hours [22]. This concentration and duration of TRF treatment were in line with a previous study which showed that SIPS cells posttreated with 24 hours 50 *μ*g/mL TRF were able to increase the proliferation capacity of myoblast cells.

### 2.3. Total RNA Extraction and Purification

Total RNA from CHQ5B cells in different treatment groups was extracted using TRI Reagent (Molecular Research Center, Cincinnati, OH, USA) according to the manufacturer's instruction. Polyacryl Carrier (Molecular Research Center) was added in each extraction to precipitate the total RNA. The extracted total RNA pellet was then washed with 75% ethanol and air-dried. The extracted total RNA was dissolved in RNase and DNase-free distilled water and purified by using the RNeasy® Mini Kit (Qiagen, USA) according to the manufacturer's instruction. Total RNA was then stored at −80°C immediately after extraction and purification. RNA concentration and purity of the extracted RNA were determined by NanoDrop (Thermo Scientific, USA). The quality of RNA was assessed by an Agilent 2100 bioanalyzer (Agilent Technologies, USA). The high quality of RNA, i.e., RNA integrity number (RIN) ranging from 7 to 10 and absorbance ratio of A260 to A280 ranging from 1.5 to 2.1, was utilized for microarray analysis (Figure S01, Supplementary Materials).

### 2.4. Gene Expression Microarray Profiling

The isolated RNA was amplified and labelled using Affymetrix GeneChip 3' IVT Express and Affymetrix GeneChip Hybridisation, Wash, and Stain Kit (Affymetrix, Santa Clara, CA, USA) according to the manufacturer's protocol. The RNA was then hybridised to the Affymetrix GeneChip PrimeView Human Gene Expression Array cartridge, washed, and scanned according to the manufacturer's protocol. The arrays from three samples each of untreated young control, SIPS control, and TRF-treated cells were scanned and processed by using the AGCC Scan Control (Affymetrix).

### 2.5. Gene Expression Microarray Data Analysis and Statistics

The raw CEL data files from microarray profiling were imported into the Partek Genomics Suite (v. 6.6; Partek, St. Louis, MO) for analysis, and two-way analysis of variance (2-way ANOVA) was applied with a fold change of 1.5 for the selection of differentially expressed genes at a significance level of *p* < 0.05. The differentially expressed gene lists were further correlated for their relevant biological function and reaction pathway by analysing the GSEA (Gene Set Enrichment Analysis) and KEGG (Kyoto Encyclopedia of Genes and Genomes) using the Partek Genomic Suite. A significance level of*p* < 0.05in the GSEA analysis to identify the significant biological process involved was observed, whereas an enrichment score of*p* < 0.05in the KEGG pathway to identify the significant pathway was observed.

### 2.6. Quantitative Real-Time PCR (qPCR)

The microarray data was validated by using qualitative qPCR. Genes for validation, i.e., GDF15, EREG, RRM2B, SHC3, SHC1, SESN1, MSTN, MYOD1, and SMAD3, were chosen from pathway analysis. By using 2 *μ*L total RNA as template and iScript Reverse Transcription Supermix (Bio-Rad, USA), the cDNA is generated from RNA. The reactions were carried out as follows: priming for 5 minutes at 25°C, then reverse-transcription for 30 minutes at 42°C and inactivation of the reverse-transcription for 5 min at 85°C.

Primer sequences for GDF15, EREG, RRM2B, SHC3, SHC1, SESN1, MSTN, MYOD1, and SMAD3 are shown in Table 1(a). qPCR was carried out by using 1 *μ*L cDNA as template, 1 *μ*L of forward and reverse primers for genes of interest, and SSoAdvanced SYBR Green Supermix (Bio-Rad, USA). All reactions were run in duplicate using Real-Time PCR iQ5 (Bio-Rad, USA). The thermal cycling profiles are presented in Table 1(b). The melt curve analysis of each pair of primers and agarose gel electrophoresis that was performed on the PCR products was used to determine the primer specificity (Figure S02, Supplementary Materials). The gene expression level of each targeted gene was normalized to that of glyceraldehyde 3-phosphate dehydrogenase (GAPDH). It was presented as relative expression value (REV) by using the 2^−ΔΔCt^ method of relative quantification and the following equation:

(1) $$\begin{array}{c} REV=2^{Ct\;value\text{ of }GAPDH-Ct\;value\text{ of the }gene\text{ of }interest}. \end{array}$$

By referring to each REV value of the targeted gene, the fold change (FC) can be calculated by using the following equation:

(2) $$\begin{array}{c} Fold\;change={REV}_{treated\;cells}/{REV}_{untreated\;cells}. \end{array}$$

### 2.7. Statistical Analysis

Microarray data was analysed by using Partek Genomic Suite (v. 6.6; Partek, St. Louis, MO), and the differentially expressed gene lists were filtered based on a fold change of 1.5 and a significance level of *p* < 0.05 by using two-way analysis of variance (2-way ANOVA). The relevant biological function and reaction pathway was identified based on GSEA analysis at a significance level of *p* < 0.05 and KEGG analysis at an enrichment score *p* < 0.05 by using the Partek Genomic Suite.

The REV data in qPCR are presented as mean ± standard error of the mean (SEM). Statistical analysis was performed with the software IBM SPSS Statistics (version 20). Independent sample *T* test was used to determine the significant differences in between the SIPS control and TRF-treated groups. For all of the tests, *p* < 0.05 was considered statistically significant.

## 3. Results

### 3.1. Quality Control Assessment of the Samples and the Hierarchical Clustering of Significantly Expressed Genes

**Figure 1:**
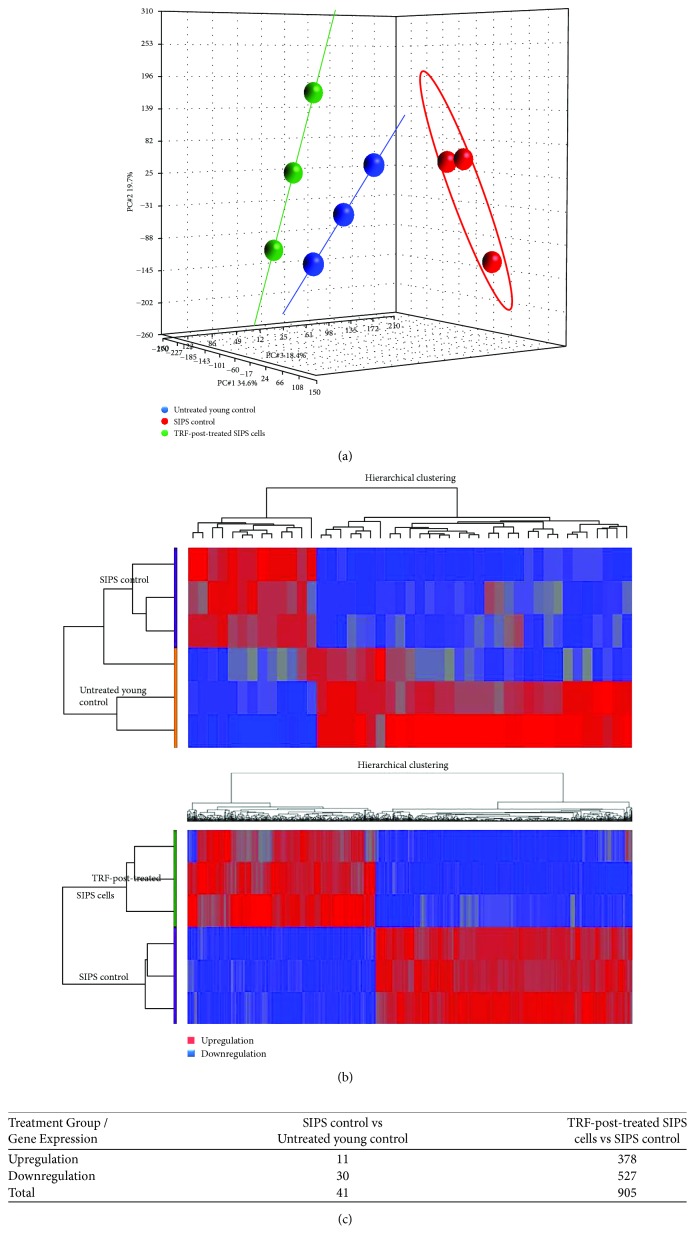
(a) PCA and (b) hierarchical clustering of the data. Clustering analysis was able to distinguish gene expression between untreated young control and SIPS control as well as between the TRF-treated group and the SIPS control group. (c) There were a total of 41 genes and 905 genes significantly expressed in between SIPS control and untreated young control and in between TRF-posttreated SIPS cells and SIPS control, respectively.

Principal component analysis (PCA) is a multivariate statistic which allows viewing of separation between groups of replicates. The untreated young control, SIPS, and TRF-posttreated groups were well separated (Figure 1(a)). Hierarchical cluster analysis was performed to organize genes into cluster based on their similarities of expression. The upregulation of gene expression was indicated in red, whereas the downregulation of gene expression was indicated in blue. Clustering analysis was able to distinguish gene expressions between untreated young control and SIPS groups as well as between TRF-posttreated and SIPS groups (Figure 1(b)).

### 3.2. Identification of Gene Expression Changes Associated with SIPS Myoblasts

The gene expression analysis using Partek Genomic Suite was performed to identify changes in the SIPS myoblasts. Statistical analysis of two-way analysis of variance (2-way ANOVA) revealed that a total of 41 genes were significantly regulated in SIPS myoblasts as compared to untreated young control cells (fold change<−1.5 or fold change > 1.5; *p* < 0.05); i.e., 11 genes were upregulated and 30 genes were downregulated (Figure 1(c)). The complete list of 41 differentially expressed genes is available in Table S01, Supplementary Materials.

### 3.3. Identification of Gene Expression Changes Associated with TRF-Post-treatment on SIPS Myoblasts

The gene expression analysis using Partek Genomic Suite was performed to identify changes in TRF-posttreated SIPS myoblasts. Statistical analysis of two-way analysis of variance (2-way ANOVA) revealed that a total of 905 genes were significantly regulated in TRF-posttreated SIPS myoblasts as compared to the SIPS group (fold change<−1.5 or fold change > 1.5; *p* < 0.05); i.e., 378 genes were upregulated and 527 genes were downregulated (Figure 1(c)). The complete list of 905 differentially expressed genes is available in Table S02, Supporting Materials. At present, only selected differentially expressed genes including growth differentiation factor 15 (*GDF15*), epiregulin (*EREG*), ribonucleotide reductase M2B (*RRM2B*), SHC (Src homology 2 domain containing) transforming protein 3 (*SHC3*), SHC transforming protein 1 (*SHC1*), sestrin 1 (*SESN1*), myostatin (MSTN), myogenic differentiation 1 (*MYOD1*), and SMAD family member 3 (*SMAD3*) that have attracted our interest will be discussed. The chosen genes regulated by TRF treatment were selected based on the biological processes and KEGG pathway analysis.

### 3.4. Biological Processes and Pathways Affected by SIPS

GSEA analysis was carried out by using the Partek Genomic Suite. GSEA analysis revealed the selected significant biological processes involved in SIPS myoblasts compared to the untreated young control group (*p* < 0.05) (Table 2). The positive value of the normalized enrichment score (NES) indicated an increment in the regulation of the biological process, whereas the negative value of NES indicated a reduction in the regulation of the stated biological process. SIPS myoblasts showed a significant increase in cellular biogenic amine metabolic process and apoptosis but decreased in activities of lipoprotein and regulation of skeletal muscle cell differentiation.

### 3.5. Biological Processes and Pathways Affected by the TRF-Post-treatment on SIPS Myoblasts

GSEA analysis revealed the selected significant biological processes involved in response to TRF-posttreatment SIPS myoblasts compared to SIPS control myoblasts (*p* < 0.05) (Table 3). TRF treatment has significantly increased the regulation of JNK cascade, cell growth, and adult walking behavior, but decreased the cell cycle activity. KEGG pathway analysis was carried out by using Partek Genomic Suite, and the data can be categorised according to the cluster of differentially expressed genes (fold change<−1.5 or fold change > 1.5; *p* < 0.05) and the pathway ANOVA statistical method (*p* < 0.05).

**Figure 2:**
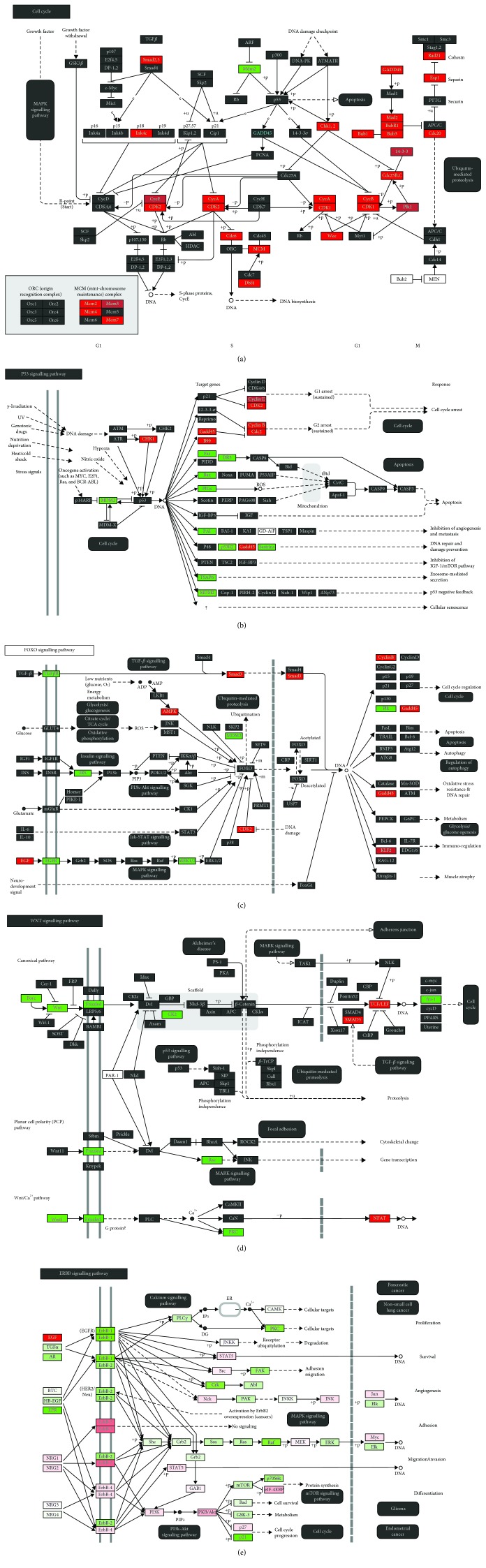
Differential gene expression involved in the KEGG pathway regulated by TRF treatment, i.e., (a) cell cycle, (b) p53 signalling pathway, (c) FoxO signalling pathway, (d) Wnt signalling pathway, and (e) ErbB signalling pathway.

Our KEGG pathway analysis showed that TRF treatment significantly modulated the p53 signalling pathway, FoxO signalling pathway, Wnt signalling pathway, cell cycle, and ErbB signalling pathway (Table 4). The regulation of gene expression involved in the stated pathway is presented in Figure 2.

### 3.6. Microarray Result Validation

**Figure 3:**
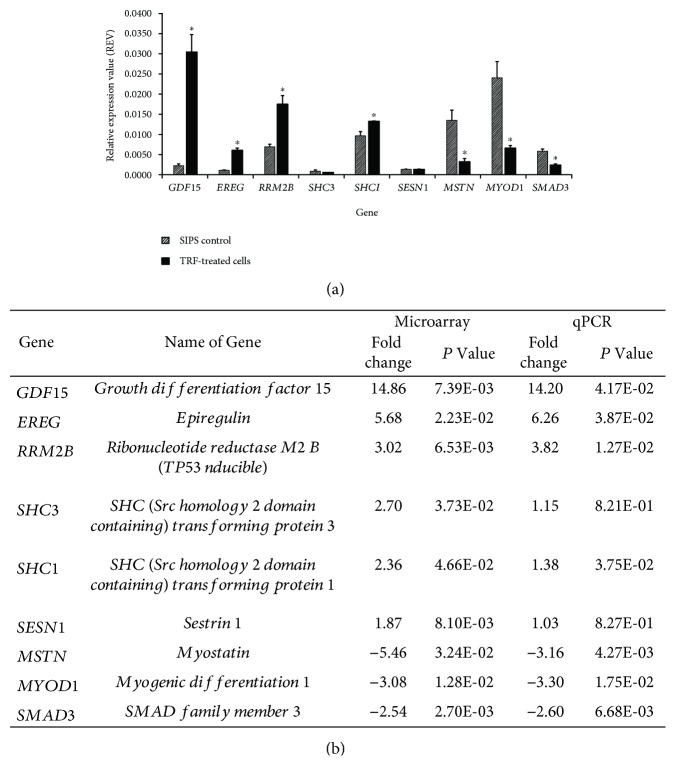
Microarray result validation. (a) The REV value and (b) the fold change were consistent between the microarray analysis and the qPCR analysis. The REV data are shown as mean ± SEM. ^∗^ *p* < 0.05 compared to SIPS control.

To confirm the microarray expression, we performed qPCR validation and found that all of the tested genes have consistent gene profiles. The REV value and fold change for all of the genes were consistent for both microarray analysis and qPCR analysis (Figure 3).

## 4. Discussion

Vitamin E, especially tocopherol, with a combination of other approaches, such as exercise, vitamin C, or selenium, has been reported to improve the condition of aged skeletal muscles by reducing oxidative stress and increasing the activities of antioxidant enzymes in the muscles of aged rats [20, 24]. Recently, one of the isomers of vitamin E, i.e., tocotrienol, has been proven for its effects in antioxidant defence enhancement and for improving the proliferation and differentiation in both replicative senescence and oxidative stress-induced premature myoblasts [22, 25, 26]. Khor et al. suggested that TRF reduced the senescence phenotypes in the skeletal muscle which may not only be limited to the encounter of oxidative stress but also instead be possibly associated with its regenerative capacity [23]. Little is known on the mechanism of tocotrienol towards the oxidative stress-associated senescence in myoblasts, especially human myoblasts. Thus, in this study, we revealed the novel insight into the differential gene expression by stress-induced human myoblasts with the treatment of TRF.

### 4.1. Alteration in Gene Expression and Biological Process in SIPS Cells

Hydrogen peroxide (H_2_O_2_) is a common inducer in the SIPS model in various cells [27, 28]. Research has shown that H_2_O_2_ downregulated the expression of miRNAs such as miR-15 and miR-106b families which contributes to some of the features of senescence cells. These features include the increased resistance to apoptosis and activation of p21^CDKN1A^. At the same time, it upregulates miR-182 which causes specific changes in senescence-associated gene expression [29]. Our GSEA analysis showed that SIPS has upregulated the cellular biogenic amine metabolic process in the cells while differential gene expression analysis showed that spermine oxidase (*SMOX*) was upregulated. Biogenic amine, such as polyamine, monoamine, and histamine, is involved in the mechanism of apoptosis progression [30]. The increasing expression of *SMOX* would increase the oxidation of spermine in the nucleus to spermidine, H_2_O_2_, and 3-aminopropanal and reduce the concentration of nuclear spermine, thus dysregulating the protective roles of spermine in free radical scavenging and DNA shielding and resulting in an overall increased potential for oxidative DNA damage in these cells [31]. Apoptosis has always been one of the suggested factors in the mass loss of skeletal muscle and muscle atrophy [32, 33], and our GSEA analysis was in line with this factor. The GSEA analysis showed the upregulation of cytochrome c released from mitochondria which is an indicator of early-stage apoptosis in SIPS myoblasts.

### 4.2. Alteration in Gene Expression, Biological Process, and Signalling Pathway in TRF-Post-treated SIPS Cells

#### 4.2.1. ErbB Signalling Pathway

KEGG pathway analysis showed that TRF treatment on SIPS myoblasts significantly regulated a few biological processes and pathways related to cell proliferation, including the ErbB signalling pathway and FoxO signalling pathway. Several ErbB signalling-related genes, such as *EGF*, *EGFR*, *EREG*, *SHC1*, and *SHC3*, were significantly regulated by the treatment of TRF. The upregulation of *SHC1* and *SHC3* expression by 2.70-fold and 2.36-fold, respectively, by TRF treatment on SIPS myoblasts suggested the involvement of TRF in modulating the ErbB pathway, hence improving the regenerative capacity of skeletal muscle via cell proliferation. A similar effect was observed in *C. elegans*, in which *SHC1* activated JNK signalling by binding to the MEK-1 kinase and hence regulating stress response and aging [34]. Epiregulin (*EREG*), an ErbB-signalling related gene, was upregulated with a 5.68-fold in TRF-posttreatment SIPS myoblasts compared to the SIPS group. Upregulation of *EREG* expression was reported before in C2C12 mouse myoblast cells after 4 hours from differentiation of myoblast cells. However, downregulation of the MRF gene and regulation of canonical Wnt signalling by the TRF on SIPS myoblasts minimised the possibilities of differentiation of human myoblasts (CHQ5B) in this study. Other studies with human cells showed that upregulation of EREG expression improved the proliferation ability of various human cells, i.e., mesenchymal stem cells, keratinocytes, and fibroblasts [35–37]. Hence, upregulation of *EREG* expression by TRF-post-treatment in SIPS myoblasts suggested a similar mechanism, i.e., modulation of the ErbB pathway which leads to increased proliferation capacity of myoblasts.

#### 4.2.2. Myostatin (MSTN)

Unpredictably, our findings showed that myostatin (*MSTN*), a negative regulator in the muscle growth and regenerative potential of skeletal muscle, was downregulated significantly by 5.46-fold. This would be a novel finding in the modulation of muscle regeneration by TRF. Exposure of myostatin to mouse C2C12 myoblast cells reduced the proliferation and protein synthesis and thus prevented the progression of cells from the G1 to S phase in the cell cycle [38, 39]. On the other hand, aside from an increment in total RNA and protein synthesis rate, mice with myostatin knockout had a larger size of myotube [40]. A study showed that muscle-associated senescence upregulated the expression of myostatin, but antagonists of myostatin activated the satellite cells and increased the protein level of Pax7 and MyoD which in turn improved the regenerative capacity of muscle cells [41, 42].

The signalling pathways for myostatin can be divided into SMAD-mediated and non-SMAD pathways. The present microarray analysis showed that the treatment of TRF on SIPS myoblasts modulated the FoxO signalling pathway through downregulation of *MSTN* and *SMAD3*. FoxO and SMAD are involved in the regulation of muscle growth through amplification of atrophy response and activation of MSTN expression [43]. SMAD3-mediated myostatin signalling increased the sarcomeric protein degradation via the ubiquitin-proteasome pathway by stimulating FoxO1 and atrogin-1 expression [44]. In this study, the downregulation of *MSTN* and *SMAD3* by TRF treatment suggested that the reduction in the SMAD-mediated myostatin signalling pathway and TRF treatment would reduce the protein degradation in the SIPS myoblasts.

#### 4.2.3. Ribonucleotide Reductase M2B (RRM2B)

Modulation of the ErbB signalling pathway and the FoxO signalling pathway by TRF treatment suggested that the mechanism involved might be related to cell proliferation. However, at the same time KEGG analysis demonstrated that TRF regulated the satellite cells to remain in the quiescent state through the p53 signalling, cell cycle, and Wnt signalling pathways. Interestingly, these findings showed a contradiction. Nevertheless, recent studies showed that the satellite cells are a heterogeneous population as they consist of satellite stem cells and committed progenitors [45]. Symmetric division of satellite cells allowed the cells to divide and expand the satellite stem cell subpopulation, whereas asymmetric division of satellite cells would maintain the stem cell population which is involved in the self-renewal and generation of myogenic progenitors [46, 47]. Dumont et al. suggested that a dynamic balance must exist in between the symmetric and asymmetric division even though the satellite cells are able to choose whether to perform symmetric or asymmetric division, and it allows them to coordinate their activity with the needs of the regenerating muscle [46]. An imbalance in the ratio of symmetric and asymmetric division would deteriorate the regenerative capacity of muscles. These were observed in the aging cells with a disruption in the satellite cells' ability to self-renew or return to the quiescent state [48, 49].

The TRF-post-treatment on SIPS myoblasts has targeted p53 signalling by upregulating the expression of the p53 target gene, i.e., *RRM2B* and *SESN1*. *RRM2B* activation is initiated by DNA damage and further involved in DNA repair regulated by p53 [50, 51]. Dysfunction of *RRM2B* was first reported in mitochondrial DNA depletion syndrome which caused early fatality in children [52, 53]. Kuo et al. reported that the expression of *RRM2B* was highly induced during oxidative stress in the human primer fibroblast (IMR90 cell) to stimulate antioxidant reaction [50]. Silencing of *RRM2B* expression leads to an increase in reactive oxygen species level, mitochondrial membrane depolarisation, and premature senescence in young fibroblasts. Thus, upregulation of *RRM2B* expression by TRF was suggested to stimulate the antioxidant defence in order to counteract with the increasing stress level in SIPS myoblasts.

#### 4.2.4. Sestrins

Aside from *RRM2B*, *SESN1* was also upregulated by the TRF. Sestrins were the third recently identified hallmark of sarcopenia [54]. Sestrin expression was upregulated in DNA damaged cells, oxidative stress condition, and hypoxia [55]. *SESN1* and *SESN2* are p53-regulated genes which are involved in the regulation of autophagy and cell viability, whereas *SESN3* is a FoxO-regulated gene which in turn activated *SESN1* [56–58]. Inhibition of mTORC1 activation via the sestrin-AMPK pathway was reported to extend the lifespan of mammalia [59–61]. A more specific study was carried out to investigate the expression of sestrins towards the skeletal muscle [62]. The thorax of adult Drosophila mainly consists of skeletal muscle enriched with Drosophila sestrins (*dSesn*). *dSesn*-null flies experienced acceleration in aging-associated degeneration, such as loss of sarcomeric structure and abnormality in mitochondria. However, treatment with vitamin E effectively prevented the deterioration in skeletal muscle and cardiac muscle by reducing the involvement of ROS [62].

GSEA and KEGG pathway analysis showed that the cell cycle was downregulated by TRF treatment in SIPS myoblasts. This finding was in contrast with the findings which reported that TRF reduced the cell cycle arrest in senescence human fibroblast cells [63]. However, according to Blagosklonny, cell cycle arrest does not absolutely refer to senescence and vice versa [64]. Cell cycle arrest actually induced the cells to proceed to another stage of the cell cycle, such as quiescence, senescence, apoptosis, motility, and differentiation [65]. Skeletal muscle satellite cells withdraw from the cell cycle for two purposes, i.e., to return to the quiescence state and carry out self-renewal or to differentiate and generate new muscle fibres. These withdrawals can be differentiated by the differential expression of genes or pathway activation. Notch signalling was activated, and *Spry1* expressions were upregulated when the cells return to the quiescence state, whereas *Delta1* expression was upregulated for the cells to differentiate after withdrawing from the cell cycle [45, 46]. However, in our study, neither *Spry1* nor *Delta1* expression was regulated by TRF treatment. Instead, the expression of *Spry4* was upregulated and the expression of *Fgf1* was downregulated by the TRF. Downregulation of *Fgf1* was once reported in the inhibition of mouse myoblast differentiation [66, 67]. Furthermore, upregulation of *Fgf2* was reported in the activation of satellite cells and in turn increased the expression of *Twist2* and *Spry4*, thus inhibiting the differentiation of mouse mesenchyme stem cells [68, 69]. Hence, we proposed that the withdrawal from the cell cycle of SIPS myoblasts posttreated with TRF was not due to differentiation, as differentiation was inhibited by downregulation of *Fgf1* and upregulation of *Spry4*. Instead, the TRF promoted the myoblast cells to exit the cell cycle and return to their quiescence state.

#### 4.2.5. Myogenic Regulatory Factor (MRF) and Wnt Signalling Pathway

The effect of TRF towards expression of the myogenic regulatory factor (MRF) was in line with the suggestion that the myoblasts exit the cell cycle for the maintenance of the quiescence cell population. The myogenic potential of satellite cells will highly depend on the expression of *Pax* and MRF (*MyoD*, *Myf5*, *myogenin*, and *MRF4*) [70]. Expression of *Myf5* and/or *MyoD* was upregulated during the activation of satellite cells to the myoblast and early myogenic differentiation [71–73], whereas differentiation of myoblasts would upregulate the expression of *myogenin* and *MRF4* (also known as *Myf6*) [73]. TRF treatment has downregulated the expression of *Myf5*, *MyoD1*, and *Myf6*, which means that TRF treatment did not promote the myoblast cells to withdraw from the cell cycle for differentiation.

The expression of MRF was regulated by the Wnt signalling pathway during the development of the embryo. However, in the adult skeletal muscle, the canonical Wnt signalling pathway regulated the differentiation of muscle satellite cells, whereas the noncanonical Wnt signalling pathway mediated the self-renewal of satellite stem cells and the growth of muscle fibres [74]. Our GSEA analysis revealed that the noncanonical Wnt signalling pathway was significantly regulated by TRF treatment. Among the Wnt involved in the noncanonical Wnt signalling pathway (*Wnt4*, *Wnt5a*, *Wnt5b*, *Wnt8a*, *Wnt8b*, *Wnt10a*, and *Wnt10b*), TRF treatment has upregulated the expression of *Wnt5a* and *Wnt7b*. Studies reported that Wnt were expressed at different times of injury in order to promote regeneration [75, 76]. *Wnt5a*, *Wnt5b*, and *Wnt7a* were upregulated at the early stage of regeneration, and *Wnt7b* and *Wnt3a* were regulated at the final stage after injury. The regulation of the Wnt signalling pathway, cell cycle, and p53 signalling by TRF treatment suggested the promotion of satellite cells to return to the quiescence state and maintain the population of quiescence cells.

#### 4.2.6. Growth Differentiation Factor 15 (GDF15)

Another critical finding of our microarray analysis was the expression of GDF15 which was highly upregulated by 14.9-fold. The expression of *GDF15* and its mechanism involved in the CHQ5B cells were not well studied. Previously, *GDF15* was reported as a negative regulator in skeletal muscle growth [77, 78]. Senescence, smoking, and environmental factors would increase the GDF15 level [79]. The levels of GDF15 increased in patients experiencing intensive care unit acquired muscle weakness (ICUAW) or muscle atrophy [80, 81]. However, aside from a high level of GDF15, SMAD2/3 was activated by GDF15 in the ICUAW patients and this was in contrast with our findings. Findings showed that SMAD3 expression was downregulated by TRF treatment. GDF15 prevents the ROS production and exhibits an antiapoptotic effect and cell proliferation regulation [78, 82, 83]. Hence, the conclusion on the activity of GDF15 towards CHQ5B cells remains unknown due to its lack of investigation. Development of therapeutic interventions with GDF15 or anti-GDF15 agents remains difficult until the mechanism that drives its activity is revealed with more evidences.

## 5. Conclusions

At present, most of the studies focused on the effect of tocopherol on the skeletal muscles, but less *in vitro* findings were reported on the effects of tocotrienol towards human skeletal muscle cells and the mechanism involved remains unclear. Recent studies showed that muscle regeneration does not solely depend on the myogenic proliferation of the satellite cell as prolonged imbalance between the expansion and maintenance of the satellite stem cell population leads to impaired muscle regeneration [46]. Our findings proposed that TRF treatment not only promotes the proliferation capacity of SIPS myoblasts through regulation of the ErbB signalling pathway (upregulation of expression of *EREG*, *SHC1*, and *SHC3*) and FoxO signalling pathway (downregulation of expression of *MSTN* and *SMAD3*). At the same time, TRF treatment is proposed to modulate the renewal of satellite cells through regulation of p53 signalling (upregulation of *RRM2B* and *SESN1*), cell cycle, Wnt signalling pathway, and expression of MRF. Even though these findings were exciting, more extensive studies such as proteomic analysis and various time points of TRF treatments in the SIPS myoblast model are suggested in order to give a more thorough idea on the involvement of TRF in the regenerative capacity and mechanism of skeletal muscles.

## Figures and Tables

**(a):** 

Accession number	Gene	Primer type	Primer sequences (5′–3′)
NM_001289746	*GAPDH*	Forward	TTGCCCTCAACGACCACTTT
		Reverse	TGGTCCAGGGGTCTTACTCC

NM_004864.2	*GDF15*	Forward	CAGGACGGTGAATGGCTCTC
		Reverse	TAGCGTTTCCGCAACTCTCG

NM_001432.2	*EREG*	Forward	CTGCAGGTGTGAAGTGGGTT
		Reverse	GTGGAACCGACGACTGTGAT

NM_015713.4	*RRM2B*	Forward	AGAGTTCTCGCCGGTTTGTC
		Reverse	GTCGACCTCTTCTGCTGTCC

NM_016848.5	*SHC3*	Forward	GCTTTTGCTGTGAGCAGCC
		Reverse	CCACCGTTAAAAGCCAGCAC

NM_183001.4	*SHC1*	Forward	CTTGGGAGCTACATTGCCTGT
		Reverse	TCAAAAAGCTCTCTGCCTGGAC

NM_014454.2	*SESN1*	Forward	TTGACAGCTCCACAACGTGA
		Reverse	TGTACACGAAAGGGCAGTCT

NM_005259.2	*MSTN*	Forward	TTGACATGAACCCAGGCACT
		Reverse	GTCCTGGGAAGGTTACAGCA

NM_002478.4	*MYOD1*	Forward	CGCCAGGATATGGAGCTACT
		Reverse	GAGTGCTCTTCGGGTTTCAG

NM_005902.3	*SMAD3*	Forward	CATGTCGTCCATCCTGCCTT
		Reverse	TTGGTGTTGACGTTCTGCGT

**(b):** 

Cycling step	Temperature	Time	Number of cycles
Enzyme activation/initial DNA denaturation	95°C	30 seconds	1
Denaturation	95°C	5 seconds	40
Annealing/extension	60°C	30 seconds	
Melt curve	65°C to 95°C (in 0.5°C increments)	5 seconds	1

**Table 2:** GSEA analysis on the biological processes regulated by the stress-induced premature senescence as compared to the untreated young control (*p* < 0.05).

*GO accession*	Biological process	NES	*p* value
GO:0006576	*Cellular biogenic amine metabolic process*	1.33	2.63*E* − 02
GO:0044106	*Cellular amine metabolic process*	1.33	2.63*E* − 02
GO:0046885	*Regulation of hormone biosynthetic process*	−1.40	3.17*E* − 02
GO:0051004	*Regulation of lipoprotein lipase activity*	−1.32	3.28*E* − 02
GO:0070874	*Negative regulation of glycogen metabolic process*	−1.44	3.39*E* − 02
GO:0032106	*Positive regulation of response to extracellular stimulus*	−1.48	3.45*E* − 02
GO:0032109	*Positive regulation of response to nutrient levels*	−1.48	3.45*E* − 02
GO:0031442	*Positive regulation of mRNA 3′-end processing*	−1.24	3.57*E* − 02
GO:0043001	*Golgi to plasma membrane protein transport*	−1.41	3.64*E* − 02
GO:2000973	*Regulation of pro-B cell differentiation*	−1.69	3.77*E* − 02
GO:0032494	*Response to peptidoglycan*	1.40	3.85*E* − 02
GO:0006400	*tRNA modification*	1.50	3.92*E* − 02
GO:0045807	*Positive regulation of endocytosis*	−1.34	4.11*E* − 02
GO:0006970	*Response to osmotic stress*	−1.15	4.17*E* − 02
GO:0090199	*Regulation of release of cytochrome c from mitochondria*	1.29	4.35*E* − 02
GO:0042953	*Lipoprotein transport*	−1.18	4.35*E* − 02
GO:0044872	*Lipoprotein localization*	−1.18	4.35*E* − 02
GO:0061418	*Regulation of transcription from RNA polymerase II promoter in response to hypoxia*	−1.52	4.35*E* − 02
GO:0048644	*Muscle organ morphogenesis*	−1.70	4.35*E* − 02
GO:0022615	*Protein to membrane docking*	−1.49	4.41*E* − 02
GO:0035563	*Positive regulation of chromatin binding*	1.55	4.44*E* − 02
GO:0048259	*Regulation of receptor-mediated endocytosis*	−1.25	4.48*E* − 02
GO:0032922	*Circadian regulation of gene expression*	−1.25	4.55*E* − 02
GO:0006694	*Steroid biosynthetic process*	−1.37	4.55*E* − 02
GO:2001014	*Regulation of skeletal muscle cell differentiation*	−1.57	4.55*E* − 02
GO:2000641	*Regulation of early endosome to late endosome transport*	−1.31	4.62*E* − 02
GO:0043112	*Receptor metabolic process*	−1.25	4.69*E* − 02
GO:0006891	*Intra-Golgi vesicle-mediated transport*	−1.38	4.69*E* − 02
GO:0035330	*Regulation of hippo signalling*	−1.44	4.69*E* − 02
GO:0035640	*Exploration behavior*	−1.47	4.69*E* − 02
GO:0006270	*DNA replication initiation*	1.46	4.76*E* − 02
GO:0031536	*Positive regulation of exit from mitosis*	1.29	4.76*E* − 02
GO:0048020	*CCR chemokine receptor binding*	−1.41	4.76*E* − 02
GO:0017144	*Drug metabolic process*	−1.42	4.76*E* − 02
GO:2000095	*Regulation of Wnt signalling pathway, planar cell polarity pathway*	−1.69	4.76*E* − 02
GO:0042347	*Negative regulation of NF-kappaB import into nucleus*	−1.37	4.84*E* − 02
GO:0046854	*Phosphatidylinositol phosphorylation*	−1.38	4.84*E* − 02
GO:0006901	*Vesicle coating*	−1.42	4.84*E* − 02
GO:0051045	*Negative regulation of membrane protein ectodomain proteolysis*	1.62	4.88*E* − 02
GO:0006335	*DNA replication-dependent nucleosome assembly*	1.51	4.88*E* − 02
GO:0034723	*DNA replication-dependent nucleosome organization*	1.51	4.88*E* − 02
GO:2000649	*Regulation of sodium ion transmembrane transporter activity*	−1.33	4.92*E* − 02
GO:0048260	*Positive regulation of receptor-mediated endocytosis*	−1.35	4.92*E* − 02
GO:0002381	*Immunoglobulin production involved in immunoglobulin-mediated immune response*	−1.36	4.92*E* − 02
GO:0018879	*Biphenyl metabolic process*	−1.45	4.92*E* − 02

**Table 3:** GSEA analysis on the biological processes regulated by the TRF-posttreated SIPS cells as compared to the SIPS control (*p* < 0.05).

*GO accession*	Biological process	NES	*p* value
GO:0009165	*Nucleotide biosynthetic process*	−1.36	1.85*E* − 02
GO:0006338	*Chromatin remodeling*	−1.76	1.85*E* − 02
GO:0046328	*Regulation of JNK cascade*	1.21	1.96*E* − 02
GO:0030968	*Endoplasmic reticulum unfolded protein response*	1.71	2.04*E* − 02
GO:0032069	*Regulation of nuclease activity*	1.65	2.04*E* − 02
GO:0016558	*Protein import into peroxisome matrix*	1.41	2.13*E* − 02
GO:0030259	*Lipid glycosylation*	1.56	2.22*E* − 02
GO:0090501	*RNA phosphodiester bond hydrolysis*	−1.64	2.22*E* − 02
GO:0071695	*Anatomical structure maturation*	1.39	2.27*E* − 02
GO:0032869	*Cellular response to insulin stimulus*	1.35	2.27*E* − 02
GO:0016049	*Cell growth*	1.55	2.38*E* − 02
GO:0019321	*Pentose metabolic process*	1.44	2.44*E* − 02
GO:2001241	*Positive regulation of the extrinsic apoptotic signalling pathway in absence of ligand*	1.29	2.44*E* − 02
GO:2001239	*Regulation of the extrinsic apoptotic signalling pathway in the absence of ligand*	1.24	2.44*E* − 02
GO:0032092	*Positive regulation of protein binding*	1.64	2.50*E* − 02
GO:0045599	*Negative regulation of fat cell differentiation*	1.29	2.56*E* − 02
GO:0071479	*Cellular response to ionizing radiation*	−1.37	3.17*E* − 02
GO:0006308	*DNA catabolic process*	−1.56	3.28*E* − 02
GO:0000082	*G1/S transition of mitotic cell cycle*	−1.77	3.28*E* − 02
GO:0044843	*Cell cycle G1/S phase transition*	−1.77	3.28*E* − 02
GO:0090025	*Regulation of monocyte chemotaxis*	1.52	3.57*E* − 02
GO:0046325	*Negative regulation of glucose import*	1.34	3.57*E* − 02
GO:0010800	*Positive regulation of peptidyl-threonine phosphorylation*	−1.28	3.57*E* − 02
GO:0007127	*Meiosis I*	−1.66	3.64*E* − 02
GO:0002689	*Negative regulation of leukocyte chemotaxis*	1.81	3.70*E* − 02
GO:0006534	*Cysteine metabolic process*	1.32	3.70*E* − 02
GO:0090231	*Regulation of spindle checkpoint*	−1.61	3.70*E* − 02
GO:1902850	*Microtubule cytoskeleton organization involved in mitosis*	−1.73	3.70*E* − 02
GO:0032200	*Telomere organization*	−1.88	3.70*E* − 02
GO:0071300	*Cellular response to retinoic acid*	1.26	3.77*E* − 02
GO:0006302	*Double-strand break repair*	−1.88	3.77*E* − 02
GO:0031064	*Negative regulation of histone deacetylation*	1.47	3.85*E* − 02
GO:0010155	*Regulation of proton transport*	1.37	3.85*E* − 02
GO:0032924	*Activin receptor signalling pathway*	−1.35	3.85*E* − 02
GO:0051181	*Cofactor transport*	1.62	3.92*E* − 02
GO:0000060	*Protein import into nucleus, translocation*	−1.71	3.92*E* − 02
GO:1902668	*Negative regulation of axon guidance*	1.67	4.00*E* − 02
GO:0043388	*Positive regulation of DNA binding*	1.39	4.00*E* − 02
GO:0043320	*Natural killer cell degranulation*	−1.34	4.00*E* − 02
GO:0010887	*Negative regulation of cholesterol storage*	1.62	4.08*E* − 02
GO:0032845	*Negative regulation of homeostatic process*	1.47	4.08*E* − 02
GO:0051896	*Regulation of protein kinase B signalling*	1.33	4.08*E* − 02
GO:0008334	*Histone mRNA metabolic process*	−1.69	4.08*E* − 02
GO:0097191	*Extrinsic apoptotic signalling pathway*	1.22	4.17*E* − 02
GO:0002686	*Negative regulation of leukocyte migration*	1.53	4.26*E* − 02
GO:0048706	*Embryonic skeletal system development*	1.36	4.26*E* − 02
GO:0097035	*Regulation of membrane lipid distribution*	1.35	4.26*E* − 02
GO:0061157	*mRNA destabilization*	−1.72	4.26*E* − 02
GO:2000615	*Regulation of histone H3-K9 acetylation*	−1.73	4.26*E* − 02
GO:0072528	*Pyrimidine-containing compound biosynthetic process*	−1.88	4.26*E* − 02
GO:1903036	*Positive regulation of response to wounding*	1.35	4.35*E* − 02
GO:1900746	*Regulation of vascular endothelial growth factor signalling pathway*	−1.53	4.35*E* − 02
GO:0009303	*rRNA transcription*	−1.45	4.41*E* − 02
GO:0045080	*Positive regulation of chemokine biosynthetic process*	1.88	4.44*E* − 02
GO:0008207	*C21-steroid hormone metabolic process*	1.69	4.44*E* − 02
GO:0050795	*Regulation of behavior*	1.51	4.44*E* − 02
GO:1902107	*Positive regulation of leukocyte differentiation*	1.28	4.44*E* − 02
GO:0017157	*Regulation of exocytosis*	1.16	4.44*E* − 02
GO:0007098	*Centrosome cycle*	−1.42	4.44*E* − 02
GO:0001946	*Lymphangiogenesis*	−1.43	4.48*E* − 02
GO:0050919	*Negative chemotaxis*	1.68	4.55*E* − 02
GO:0060338	*Regulation of the type I interferon-mediated signalling pathway*	1.65	4.55*E* − 02
GO:0050748	*Negative regulation of the lipoprotein metabolic process*	1.56	4.55*E* − 02
GO:0038031	*Noncanonical Wnt signalling pathway via JNK cascade*	1.53	4.55*E* − 02
GO:0010935	*Regulation of macrophage cytokine production*	1.49	4.55*E* − 02
GO:0060907	*Positive regulation of macrophage cytokine production*	1.40	4.55*E* − 02
GO:0034661	*ncRNA catabolic process*	−1.71	4.55*E* − 02
GO:0090503	*RNA phosphodiester bond hydrolysis, exonucleolytic*	−1.59	4.62*E* − 02
GO:1902624	*Positive regulation of neutrophil migration*	1.69	4.65*E* − 02
GO:2000406	*Positive regulation of T cell migration*	1.63	4.65*E* − 02
GO:2000403	*Positive regulation of lymphocyte migration*	1.56	4.65*E* − 02
GO:1901725	*Regulation of histone deacetylase activity*	2.14	4.76*E* − 02
GO:1902667	*Regulation of axon guidance*	1.81	4.76*E* − 02
GO:0048841	*Regulation of axon extension involved in axon guidance*	1.73	4.76*E* − 02
GO:0050882	*Voluntary musculoskeletal movement*	1.51	4.76*E* − 02
GO:0042113	*B cell activation*	−1.19	4.76*E* − 02
GO:0072522	*Purine-containing compound biosynthetic process*	−1.29	4.76*E* − 02
GO:1901991	*Negative regulation of mitotic cell cycle phase transition*	−1.47	4.76*E* − 02
GO:0006919	*Activation of cysteine-type endopeptidase activity involved in apoptotic process*	−1.23	4.84*E* − 02
GO:2000757	*Negative regulation of peptidyl-lysine acetylation*	−1.80	4.84*E* − 02
GO:0035067	*Negative regulation of histone acetylation*	−1.83	4.84*E* − 02
GO:0007628	*Adult walking behavior*	1.94	4.88*E* − 02
GO:0021756	*Striatum development*	−1.31	4.92*E* − 02
GO:1901988	*Negative regulation of cell cycle phase transition*	−1.46	4.92*E* − 02
GO:0045185	*Maintenance of protein location*	−1.48	4.92*E* − 02
GO:0051053	*Negative regulation of DNA metabolic process*	−1.52	4.92*E* − 02
GO:0051258	*Protein polymerization*	−1.53	4.92*E* − 02
GO:0044774	*Mitotic DNA integrity checkpoint*	−1.68	4.92*E* − 02
GO:0009124	*Nucleoside monophosphate biosynthetic process*	−1.71	4.92*E* − 02
GO:0043487	*Regulation of RNA stability*	−1.75	4.92*E* − 02
GO:0034501	*Protein localization to kinetochore*	−1.82	4.92*E* − 02
GO:0000077	*DNA damage checkpoint*	−1.91	4.92*E* − 02
GO:0000079	*Regulation of cyclin-dependent protein serine/threonine kinase activity*	−2.01	4.92*E* − 02

**(a):** 

*KEGG entry*	Pathway	NES	*p* value
hsa04110	*Cell cycle*	25.93	5.50*E* − 12
hsa03030	*DNA replication*	20.99	7.64*E* − 10
hsa04115	*p53 signalling pathway*	16.76	5.25*E* − 08
hsa05206	*MicroRNAs in cancer*	16.08	1.03*E* − 07
hsa03460	*Fanconi anemia pathway*	14.93	3.29*E* − 07
hsa05203	*Viral carcinogenesis*	14.45	5.30*E* − 07
hsa05200	*Pathways in cancer*	12.88	2.54*E* − 06
hsa03430	*Mismatch repair*	10.17	3.81*E* − 05
hsa05322	*Systemic lupus erythematosus*	10.00	4.53*E* − 05
hsa04114	*Oocyte meiosis*	9.11	1.11*E* − 04
hsa05034	*Alcoholism*	8.64	1.76*E* − 04
hsa03420	*Nucleotide excision repair*	7.96	3.50*E* − 04
hsa03440	*Homologous recombination*	6.78	1.13*E* − 03
hsa00900	*Terpenoid backbone biosynthesis*	6.72	1.21*E* − 03
hsa05219	*Bladder cancer*	6.38	1.70*E* − 03
hsa05166	*HTLV-I infection*	6.16	2.11*E* − 03
hsa00310	*Lysine degradation*	6.02	2.43*E* − 03
hsa05210	*Colorectal cancer*	5.56	3.86*E* − 03
hsa04914	*Progesterone-mediated oocyte maturation*	5.20	5.52*E* − 03
hsa04068	*FoxO signalling pathway*	5.10	6.10*E* − 03
hsa05205	*Proteoglycans in cancer*	4.75	8.61*E* − 03
hsa04978	*Mineral absorption*	4.50	1.11*E* − 02
hsa04390	*Hippo signalling pathway*	4.49	1.12*E* − 02
hsa05222	*Small cell lung cancer*	4.23	1.46*E* − 02
hsa05217	*Basal cell carcinoma*	4.06	1.72*E* − 02
hsa05212	*Pancreatic cancer*	4.04	1.76*E* − 02
hsa00480	*Glutathione metabolism*	3.40	3.34*E* − 02
hsa05214	*Glioma*	3.17	4.22*E* − 02
hsa04310	*Wnt signalling pathway*	3.16	4.25*E* − 02

**(b):** 

*KEGG entry*	Pathway	*p* value	Fold change
hsa05323	*Rheumatoid arthritis*	8.98*E* − 03	1.13
hsa04630	*Jak-STAT signalling pathway*	1.12*E* − 02	1.07
hsa04978	*Mineral absorption*	1.56*E* − 02	1.13
hsa00860	*Porphyrin and chlorophyll metabolism*	1.89*E* − 02	1.14
hsa04340	*Hedgehog signalling pathway*	1.93*E* − 02	1.10
hsa00520	*Amino sugar and nucleotide sugar metabolism*	2.06*E* − 02	1.11
hsa05217	*Basal cell carcinoma*	2.12*E* − 02	1.10
hsa00561	*Glycerolipid metabolism*	2.18*E* − 02	1.09
hsa00630	*Glyoxylate and dicarboxylate metabolism*	2.20*E* − 02	−1.16
hsa04330	*Notch signalling pathway*	2.22*E* − 02	1.08
hsa00564	*Glycerophospholipid metabolism*	2.33*E* − 02	1.07
hsa00100	*Steroid biosynthesis*	2.46*E* − 02	−1.20
hsa00040	*Pentose and glucuronate interconversions*	2.66*E* − 02	1.16
hsa00051	*Fructose and mannose metabolism*	2.76*E* − 02	1.10
hsa03050	*Proteasome*	2.79*E* − 01	1.11
hsa00980	*Metabolism of xenobiotics by cytochrome P450*	3.08*E* − 02	1.08
hsa00650	*Butanoate metabolism*	3.11*E* − 02	−1.13
hsa00052	*Galactose metabolism*	3.13*E* − 02	1.12
hsa04640	*Hematopoeitic cell lineage*	3.22*E* − 02	1.08
hsa03060	*Protein export*	3.34*E* − 02	1.13
hsa04130	*SNARE interactions in vesicular transport*	3.35*E* − 02	1.08
hsa04110	*Cell cycle*	3.39*E* − 02	−1.31
hsa04966	*Collecting duct acid secretion*	3.48*E* − 02	1.23
hsa03430	*Mismatch repair*	3.68*E* − 02	−1.38
hsa03030	*DNA replication*	3.70*E* − 02	−1.80
hsa03460	*Fanconi anemia pathway*	3.79*E* − 02	−1.29
hsa00533	*Glycosaminoglycan biosynthesis-keratan sulfate*	3.99*E* − 02	1.12
hsa04012	*ErbB signalling pathway*	4.04*E* − 02	1.05
hsa03450	*Nonhomologous end-joining*	4.25*E* − 02	−1.19
hsa04070	*Phosphatidylinositol signalling system*	4.28*E* − 02	1.06
hsa00190	*Oxidative phosphorylation*	4.33*E* − 02	1.08
hsa00730	*Thiamine metabolism*	4.41*E* − 02	1.17
hsa03440	*Homologous recombination*	4.43*E* − 02	−1.16
hsa00982	*Drug metabolism - cytochrome P450*	4.55*E* − 02	1.04
hsa00072	*Synthesis and degradation of ketone bodies*	4.65*E* − 02	−1.27
hsa00532	*Glycosaminoglycan biosynthesis - chondroitin sulfate/dermatan sulfate*	4.69*E* − 02	1.15
hsa04360	*Axon guidance*	4.74*E* − 02	1.05
hsa00280	*Valine, leucine, and isoleucine degradation*	4.93*E* − 02	−1.07
hsa04210	*Apoptosis*	4.96*E* − 02	1.06
hsa00300	*Lysine biosynthesis*	4.99*E* − 02	−1.47

## Data Availability

The data used to support the findings of this study are included within the article and supplementary information files.

## Abbreviations

AGCC: Affymetrix GeneChip Command Console
aRNA: Amplified RNA
CEL: Cell intensity file
Ct: Threshold cycle
DMEM: Dulbecco's modified Eagle medium
EGFR: Epidermal growth factor receptor
EREG: Epiregulin
GAPDH: Glyceraldehyde 3-phosphate dehydrogenase
GDF15: Growth differentiation factor 15
GSEA: Gene set enrichment analysis
H_2_O_2_: Hydrogen peroxide
KEGG: Kyoto Encyclopedia of Genes and Genomes
MRF: Myogenic regulatory factor
Myf5: Myogenic factor 5
MyoD: Myogenic differentiation 1
PCA: Principal component analysis
qPCR: Quantitative real-time polymerase chain reaction
REV: Relative expression value
RIN: RNA integrity number
SIPS: Stress-induced premature senescence
SMOX: Spermine oxidase
TRF: Tocotrienol-rich fraction.

## Supplementary Materials

Supplementary Materials Figure S01: RNA integrity number (RIN) assessed by the electropherogram bioanalyzer for the (a) untreated young control cells, (b) SIPS control, and (c) TRF-posttreated SIPS cells. Figure S02: the specificity of primers assessed by (a–j) standard curve and melt curve analysis of each pair of primers and (k) agarose gel electrophoresis that was performed on the PCR products. Table S01: the complete list of 41 differentially expressed genes associated with stress-induced premature senescent control cells as compared to untreated young control cells (fold change < −1.5 or fold change > 1.5; *p* < 0.05). Table S02: the complete list of 905 differentially expressed genes associated with TRF-posttreated SIPS cells as compared to SIPS control cells (fold change < −1.5 or fold change > 1.5; *p* < 0.05).
