# Supplementary material for: Tocotrienol-Rich Fraction (TRF) Treatment Promotes Proliferation Capacity of Stress-Induced Premature Senescence Myoblasts and Modulates the Renewal of Satellite Cells: Microarray Analysis
Source: Oxid Med Cell Longev. 2019 Jan 10;2019:9141343. doi: 10.1155/2019/9141343 (PMC6350575; doi:10.1155/2019/9141343)
Supplement: Supplementary Materials — Figure S01: RNA integrity number (RIN) assessed by the electropherogram bioanalyzer for the (a) untreated young control cells, (b) SIPS control, and (c) TRF-posttreated SIPS cells. Figure S02: the specificity of primers assessed by (a–j) standard curve and melt curve analysis of each pair of primers and (k) agarose gel electrophoresis that was performed on the PCR products. Table S01: the complete list of 41 differentially expressed genes associated with stress-induced premature senescent control cells as compared to untreated young control cells (fold change < −1.5 or fold change > 1.5; p < 0.05). Table S02: the complete list of 905 differentially expressed genes associated with TRF-posttreated SIPS cells as compared to SIPS control cells (fold change < −1.5 or fold change > 1.5; p < 0.05). [file 9141343.f1.docx]

**Supplementary Materials**

**Figure S01** RNA integrity number (RIN) assessed by the electropherogram bioanalyzer for the (a) untreated young control cells, (b) SIPS control and (c) TRF-post-treated SIPS cells.

(a)


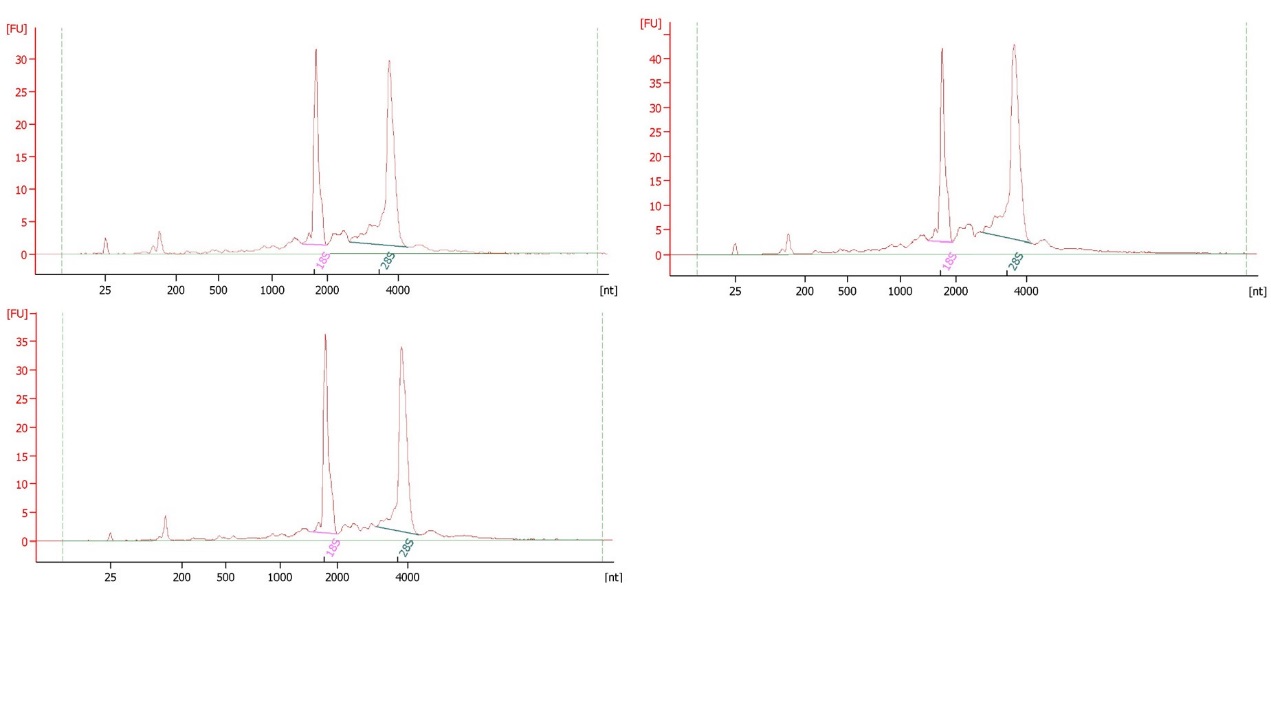


RIN: 9.30

RIN: 8.90

RIN: 9.20

(b)


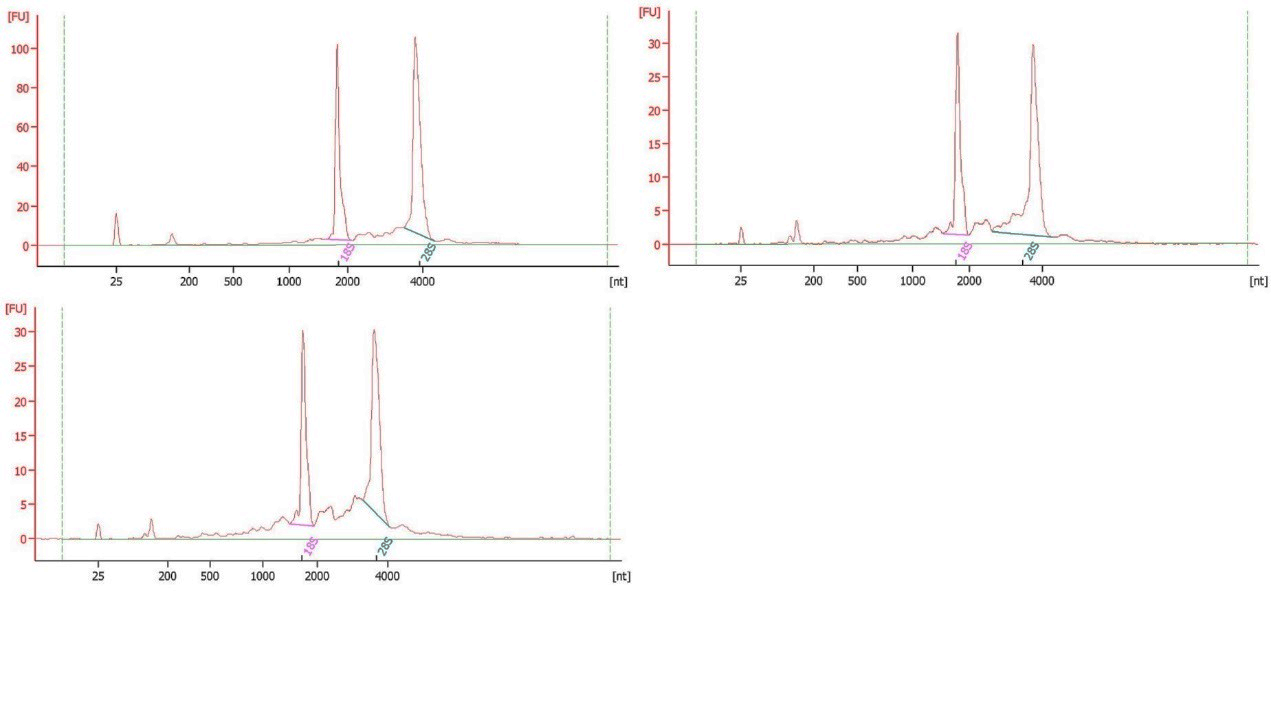


RIN: 8.50

RIN: 8.70

RIN: 9.60

(c)

RIN: 9.10


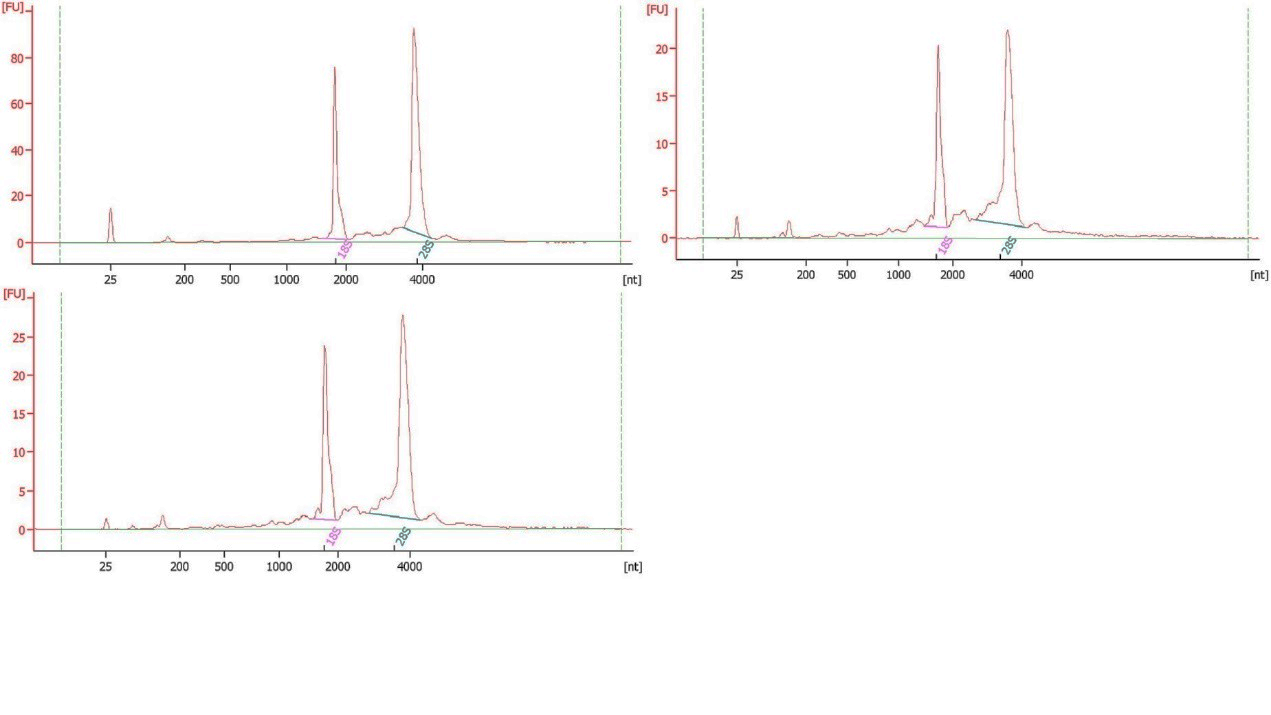


RIN: 9.30

RIN: 10

**Figure S02** The specificity of primers assessed by (a – j) standard curve and melt curve analysis of each pair of primers and (k) agarose gel electrophoresis that was performed on the PCR products.

**(a) *GAPDH***

i) Standard Curve

**
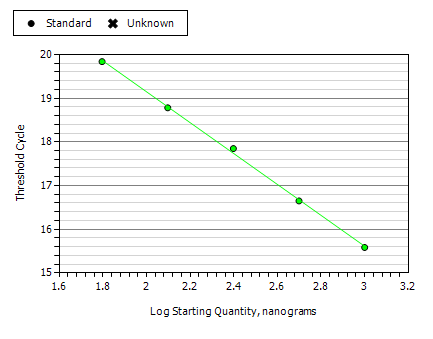
**

PCR efficiency: 91.9 %; R^2^: 0.999

Slope: -3.534; $y$-intercept: 26.215

ii) Melt curve

**
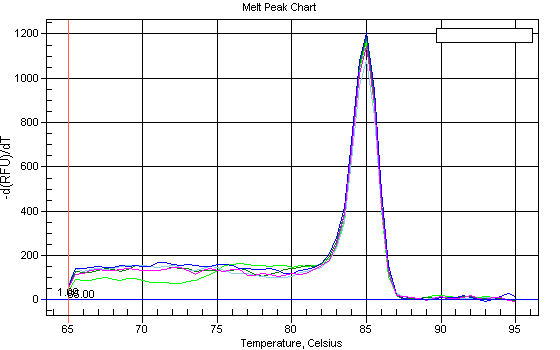
**

**(b) *GDF15***

i) Standard Curve

**
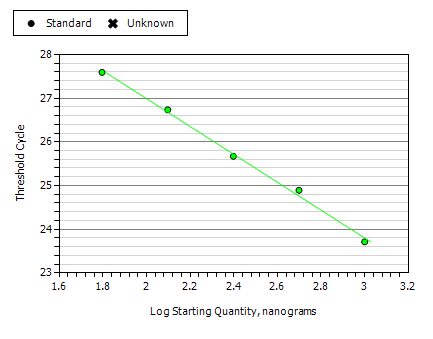
**

PCR efficiency: 105.8 %; R^2^: 0.996

Slope: -3.190; $y$-intercept: 33.370

ii) Melt Curve

**
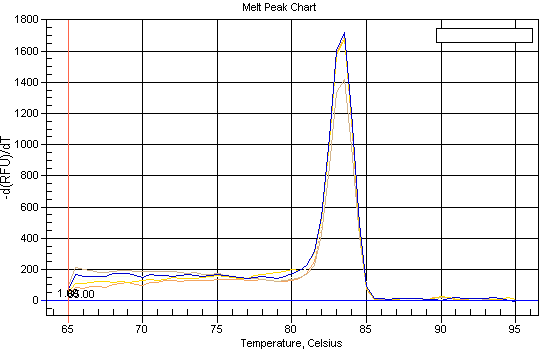
**

**(c) *EREG***

i) Standard Curve

**
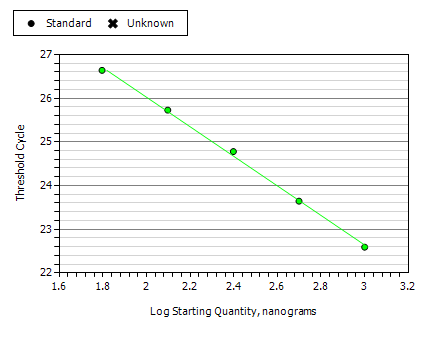
**

PCR efficiency: 97.6 %; R^2^: 0.998

Slope: -3.380; $y$-intercept: 32.782

ii) Melt Curve

**
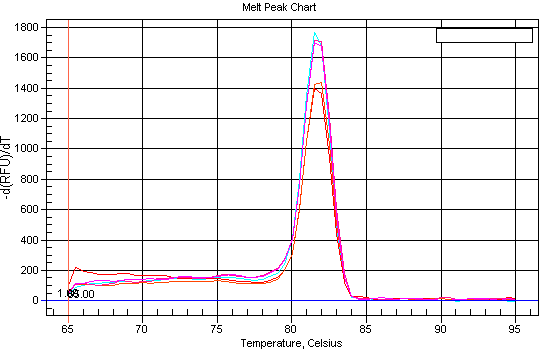
**

**(d) *RRM2B***

i) Standard Curve

**
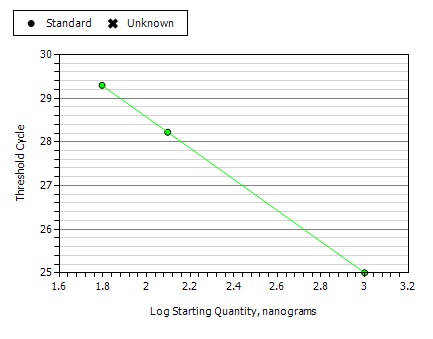
**

PCR efficciency: 90.9 %; R^2^: 1.000

Slope: -3.562; $y$-intercept: 35.691

ii) Melt Curve

**
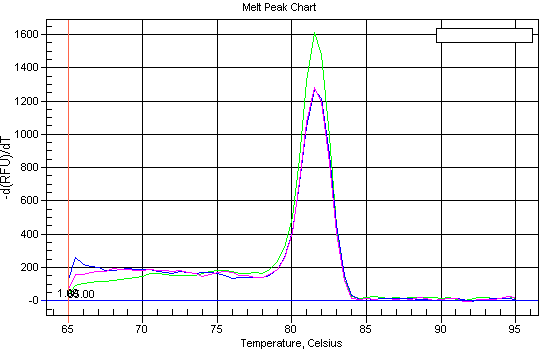
**

**(e) *SHC3***

i) Standard Curve

**
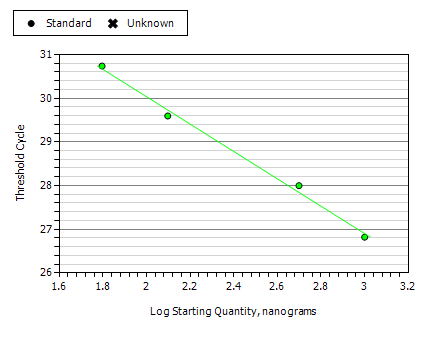
**

PCR efficiency: 108.6 %; R^2^: 0.994

Slope: -3.132; $y$-intercept: 36.298

ii) Melt Curve

**
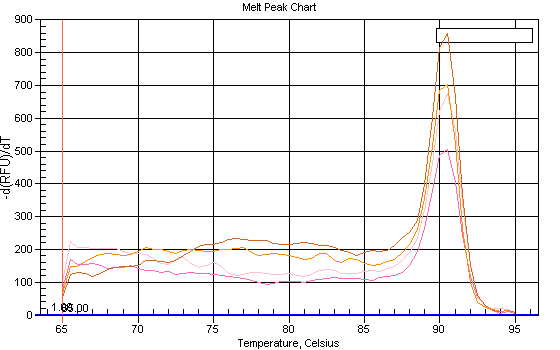
**

**(f) *SHC1***

i) Standard Curve


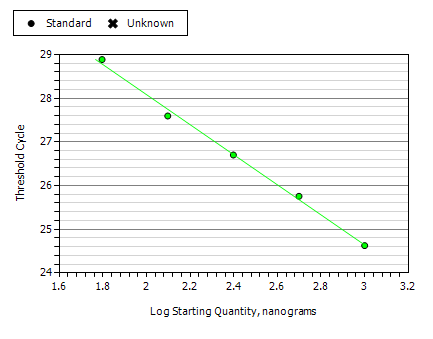


PCR efficiency: 95.3 %; R^2^: 0.996

Slope: -3.440; $y$-intercept: 34.962

ii) Melt Curve


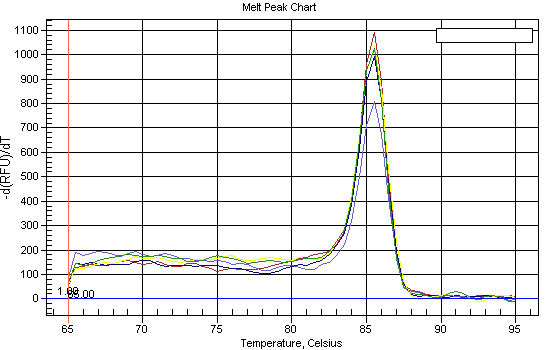


**(g) *SESN1***

i) Standard Curve

**
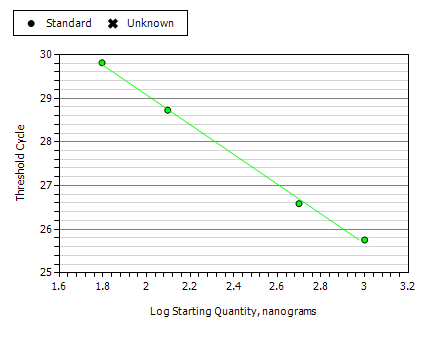
**

PCR efficiency: 96.7 %; R^2^: 0.998

Slope: -3.405; $y$-intercept: 35.883

ii) Melt Curve

**
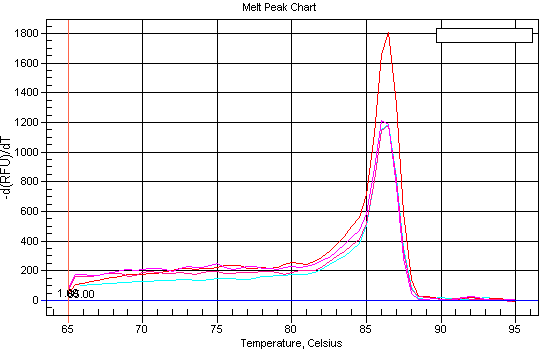
**

**(h) *MSTN***

i) Standard Curve

**
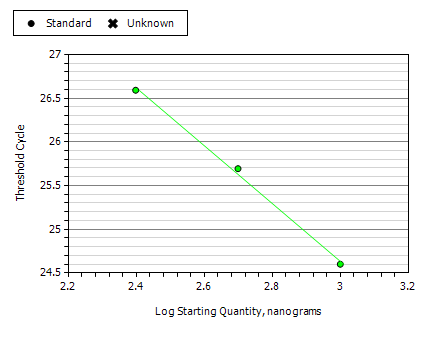
**

PCR efficiency: 100.6 %; R^2^: 0.997

Slope: -3.307; $y$-intercept: 34.554

ii) Melt Curve

**
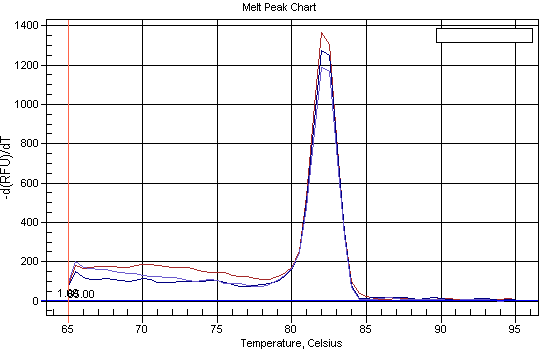
**

**(i) *MYOD1***

i) Standard Curve


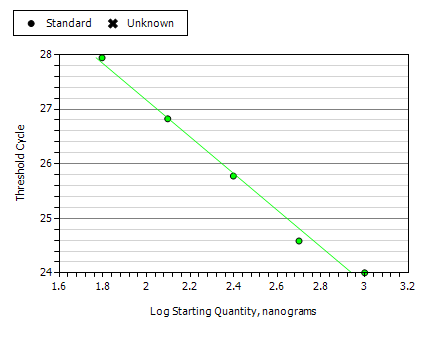


PCR efficiency: 98.5 %; R^2^: 0.990

Slope: -3.359; $y$-intercept: 33.881

ii) Melt Curve


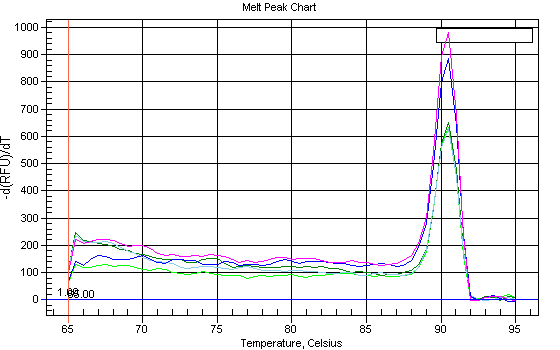


**(j) *SMAD3***

i) Standard Curve

**
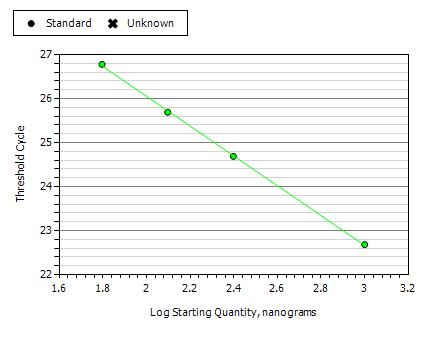
**

PCR efficiency: 97.3 %; R^2^: 1.000

Slope: -3.388; $y$-intercept: 32.830

ii) Melt Curve

**
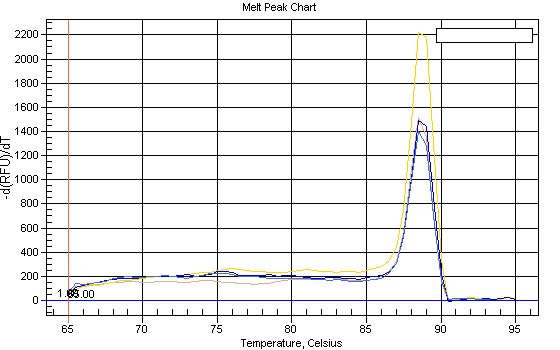
**

(k)

DNA ladder 1 kb

200 bp

100 bp

1

2

3

4

5

6


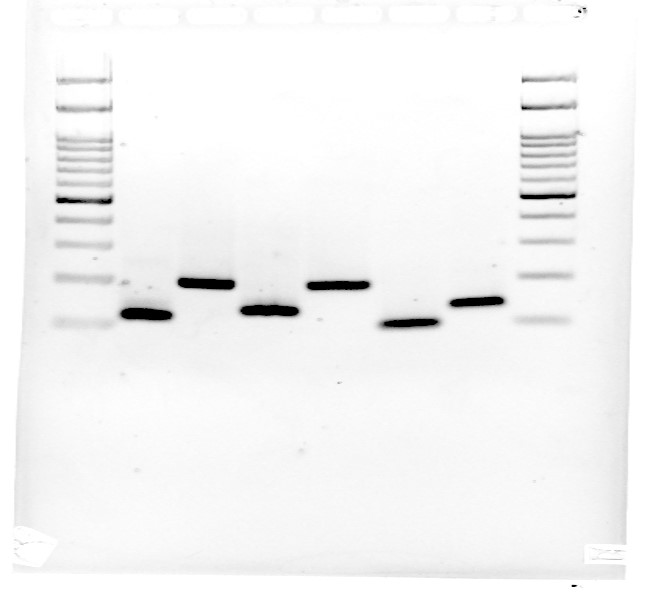


DNA ladder 1 kb

200 bp

100 bp

7

8

9

10


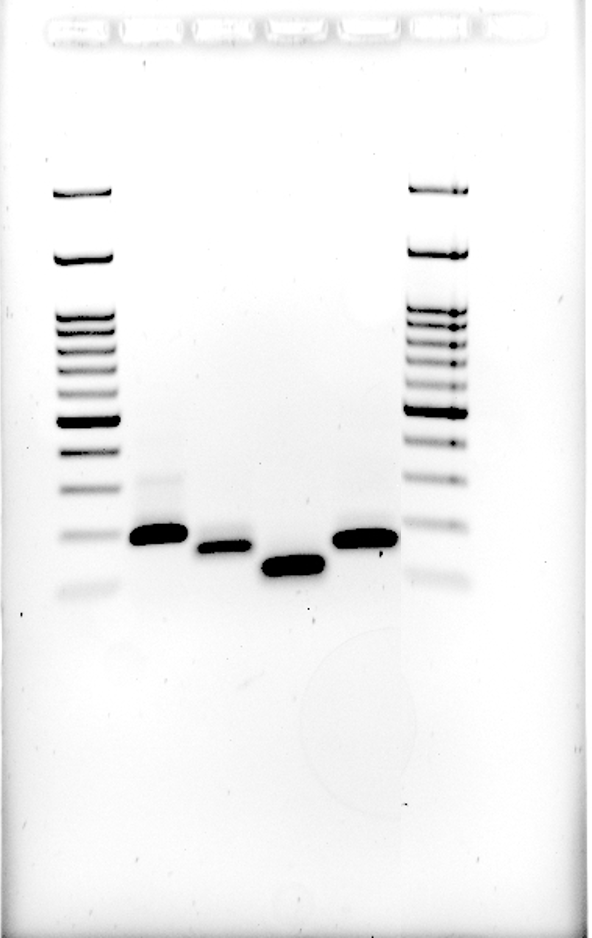


(1) *GAPDH* (120 bp), (2) *GDF15* (162 bp), (3) *EREG* (138 bp), (4) *MYOD1* (189 bp), (5) *SHC1* (102 bp), (6) *SHC3* (141 bp), (7) *SMAD3* (189 bp), (8) *SESN1* (150 bp), (9) *RRM2B* (101 bp) and (10) *MSTN* (150 bp).

**Table S01** The complete list of 41 differentially expressed genes associated with stress-induced premature senescent control cells as compared to untreated young control cells (Fold change < –1.5 or fold change > 1.5; p < 0.05)

| *RefSeq Transcript ID* | Gene Symbol | Name of Gene | P Value | Fold Change |
| --- | --- | --- | --- | --- |
| NM_030928 | *CDT1* | *chromatin licensing and DNA replication factor 1* | 2.36E-02 | 1.72 |
| NM_001128217 /// NM_021144 /// NM_033222 | *PSIP1* | *PC4 and SFRS1 interacting protein 1* | 2.46E-02 | 1.59 |
| NM_130847 | *AMOTL1* | *angiomotin like 1* | 2.86E-02 | 1.57 |
| NM_001199196 /// NM_033415 | *ARMC6* | *armadillo repeat containing 6* | 7.20E-03 | 1.54 |
| NM_004596 | *SNRPA* | *small nuclear ribonucleoprotein polypeptide A* | 4.81E-02 | 1.54 |
| NM_001270691 /// NM_175839 /// NM_175840 /// NM_175841 /// NM_175842 | *SMOX* | *spermine oxidase* | 2.89E-02 | 1.52 |
| NM_001166111 /// NM_001166112 /// NM_001166113 /// NM_001166114 /// NM_006702 | *PNPLA6* | *patatin-like phospholipase domain containing 6* | 4.97E-02 | 1.52 |
| NM_152735 | *ZBTB9* | *zinc finger and BTB domain containing 9* | 3.62E-02 | 1.52 |
| NM_018266 /// NR_073506 | *TMEM39A* | *transmembrane protein 39A* | 4.77E-02 | 1.51 |
| NM_024319 | *C1orf35* | *chromosome 1 open reading frame 35* | 1.42E-03 | 1.50 |
| NM_006810 /// NR_028444 | *PDIA5* | *protein disulfide isomerase family A, member 5* | 2.34E-03 | 1.50 |
| NM_001113239 /// NM_022740 | *HIPK2* | *homeodomain interacting protein kinase 2* | 1.75E-02 | -1.50 |
| NM_018112 | *TMEM38B* | *transmembrane protein 38B* | 3.89E-02 | -1.50 |
| NM_001271675 /// NM_014827 | *LOC441155 /// ZC3H11A* | *zinc finger CCCH-type domain-containing-like /// zinc finger CCCH-type containing 11A* | 3.76E-02 | -1.51 |
| NM_001111319 /// NM_024949 | *CLDN22 /// WWC2* | *claudin 22 /// WW and C2 domain containing 2* | 2.59E-02 | -1.51 |
| NM_001142568 /// NM_001276286 /// NM_020235 | *BBX* | *bobby sox homolog (Drosophila)* | 1.16E-02 | -1.51 |
| NM_173500 | *TTBK2* | *tau tubulin kinase 2* | 2.02E-02 | -1.52 |
| NM_017637 | *BNC2* | *basonuclin 2* | 3.66E-02 | -1.52 |
| NM_175856 | *CHSY3* | *chondroitin sulfate synthase 3* | 4.06E-02 | -1.52 |
| NM_013293 | *TRA2A* | *transformer 2 alpha homolog (Drosophila)* | 1.67E-02 | -1.53 |
| NM_014445 | *SERP1* | *stress-associated endoplasmic reticulum protein 1* | 4.97E-02 | -1.53 |
| NM_001077199 /// NM_001270492 /// NM_001270493 /// NM_139168 | *SREK1* | *splicing regulatory glutamine/lysine-rich protein 1* | 3.34E-02 | -1.53 |
| NM_004120 | *GBP2* | *guanylate binding protein 2, interferon-inducible* | 4.45E-02 | -1.54 |
| NM_003605 /// NM_181672 /// NM_181673 | *OGT* | *O-linked N-acetylglucosamine (GlcNAc) transferase* | 2.18E-02 | -1.54 |
| NM_001164315 /// NM_025190 | *ANKRD36 /// ANKRD36B* | *ankyrin repeat domain 36 /// ankyrin repeat domain 36B* | 4.37E-02 | -1.55 |
| NM_001171020 /// NM_152423 | *MUM1L1* | *melanoma associated antigen (mutated) 1-like 1* | 1.57E-02 | -1.58 |
| NM_001037324 /// NM_001100120 /// NM_001100121 /// NM_014693 /// NM_032331 | *ECE2* | *endothelin converting enzyme 2* | 2.53E-02 | -1.60 |
| NM_001159699 /// NM_001159700 /// NM_001159701 /// NM_001159702 /// NM_001159703 | *FHL1* | *four and a half LIM domains 1* | 1.65E-02 | -1.61 |
| NM_001080855 /// NM_001243756 /// NM_002859 /// NM_025157 | *PXN* | *paxillin* | 4.10E-05 | -1.61 |
| NM_001252668 /// NM_001252669 /// NM_016315 /// NR_045562 /// NR_045563 | *GULP1* | *GULP, engulfment adaptor PTB domain containing 1* | 3.16E-02 | -1.65 |
| NM_001135643 /// NM_001135644 /// NM_016221 | *DCTN4* | *dynactin 4 (p62)* | 2.56E-02 | -1.67 |
| NR_003604 /// NR_003605 /// NR_003606 /// NR_036658 /// NR_036659 | *ZFAS1* | *ZNFX1 antisense RNA 1* | 2.99E-02 | -1.68 |
| NM_030915 | *LBH* | *limb bud and heart development* | 2.98E-02 | -1.68 |
| NM_174908 /// NM_178335 | *CCDC50* | *coiled-coil domain containing 50* | 1.78E-03 | -1.69 |
| NM_000885 | *ITGA4* | *integrin, alpha 4 (antigen CD49D, alpha 4 subunit of VLA-4 receptor)* | 4.66E-02 | -1.81 |
| NM_001012969 /// NM_001159280 | *ADAL* | *adenosine deaminase-like* | 2.73E-02 | -1.82 |
| NM_015938 | *NMD3* | *NMD3 homolog (S. cerevisiae)* | 4.76E-02 | -1.88 |
| NM_001256410 /// NM_001256411 /// NM_001256412 /// NM_001256415 /// NM_021252 | *RAB18* | *RAB18, member RAS oncogene family* | 2.72E-02 | -1.89 |
| NM_016277 /// NM_183227 | *RAB23* | *RAB23, member RAS oncogene family* | 3.66E-02 | -1.94 |
| NM_001023567 /// NM_181077 /// NR_027409 /// NR_027410 | *GOLGA8A /// GOLGA8B* | *golgin A8 family, member A /// golgin A8 family, member B* | 1.80E-02 | -2.17 |
| NM_005259 | *MSTN* | *myostatin* | 1.00E-02 | -2.42 |

**Table S02** The complete list of 905 differentially expressed genes associated with associated with TRF-post-treated SIPS cells as compared to SIPS control cells (Fold change < –1.5 or fold change > 1.5; p < 0.05)

| *RefSeq Transcript ID* | Gene Symbol | | | | Name of Gene | P Value | Fold Change |
| --- | --- | --- | --- | --- | --- | --- | --- |
| NM_004864 | *GDF15* | | | | *growth differentiation factor 15* | 7.39E-03 | 14.86 |
| NM_001039667 /// NM_016109 /// NM_139314 | *ANGPTL4* | | | | *angiopoietin-like 4* | 7.17E-03 | 8.14 |
| NM_002203 /// NR_073103 /// NR_073104 /// NR_073105 /// NR_073106 /// NR_073107 | *ITGA2* | | | | *integrin, alpha 2 (CD49B, alpha 2 subunit of VLA-2 receptor)* | 1.29E-02 | 7.70 |
| NM_001353 | *AKR1C1* | | | | *aldo-keto reductase family 1, member C1* | 1.25E-03 | 5.95 |
| NM_001432 | *EREG* | | | | *epiregulin* | 2.23E-02 | 5.68 |
| NM_004163 | *RAB27B* | | | | *RAB27B, member RAS oncogene family* | 3.62E-03 | 5.00 |
| NM_020299 | *AKR1B10* | | | | *aldo-keto reductase family 1, member B10 (aldose reductase)* | 5.49E-03 | 4.91 |
| NM_002422 | *MMP3* | | | | *matrix metallopeptidase 3 (stromelysin 1, progelatinase)* | 1.71E-02 | 4.69 |
| NM_031866 | *FZD8* | | | | *frizzled family receptor 8* | 9.95E-03 | 4.61 |
| NM_001135241 /// NM_001353 /// NM_001354 /// NM_205845 | *AKR1C1 /// AKR1C2* | | | | *aldo-keto reductase family 1, member C1 /// aldo-keto reductase family 1, member C2* | 9.48E-03 | 4.41 |
| NM_001105543 /// NM_020911 /// NM_181775 | *PLXNA4* | | | | *plexin A4* | 4.97E-02 | 4.36 |
| NM_001144757 /// NM_003020 | *SCG5* | | | | *secretogranin V (7B2 protein)* | 4.39E-02 | 4.24 |
| NM_000212 | *ITGB3* | | | | *integrin, beta 3 (platelet glycoprotein IIIa, antigen CD61)* | 3.50E-02 | 3.93 |
| NM_031866 | *FZD8* | | | | *frizzled family receptor 8* | 8.35E-03 | 3.87 |
| NM_001946 /// NM_022652 | *DUSP6* | | | | *dual specificity phosphatase 6* | 1.27E-02 | 3.75 |
| NM_001042467 /// NM_024101 | *MLPH* | | | | *melanophilin* | 3.42E-03 | 3.72 |
| NM_000422 /// NM_002230 /// NM_021991 | *JUP /// KRT17* | | | | *junction plakoglobin /// keratin 17* | 4.84E-02 | 3.64 |
| NM_001256105 /// NM_003392 | *WNT5A* | | | | *wingless-type MMTV integration site family, member 5A* | 1.16E-02 | 3.61 |
| NM_203403 | *LURAP1L* | | | | *leucine rich adaptor protein 1-like* | 2.80E-03 | 3.61 |
| NM_001255976 /// NM_020182 /// NM_199169 /// NM_199170 /// NM_199171 | *PMEPA1* | | | | *prostate transmembrane protein, androgen induced 1* | 2.25E-02 | 3.52 |
| NM_176891 | *IFNE* | | | | *interferon, epsilon* | 1.74E-02 | 3.28 |
| NM_001143948 /// NM_032744 | *ADTRP* | | | | *androgen-dependent TFPI-regulating protein* | 3.94E-03 | 3.20 |
| NM_007350 | *PHLDA1* | | | | *pleckstrin homology-like domain, family A, member 1* | 6.42E-03 | 3.08 |
| NM_001128615 /// NM_001128616 /// NM_019555 | *ARHGEF3* | | | | *Rho guanine nucleotide exchange factor (GEF) 3* | 2.10E-02 | 3.04 |
| NM_145263 | *SPATA18* | | | | *spermatogenesis associated 18* | 7.70E-03 | 3.03 |
| NM_001172477 /// NM_001172478 /// NM_015713 | *RRM2B* | | | | *ribonucleotide reductase M2 B (TP53 inducible)* | 6.53E-03 | 3.02 |
| NM_001127496 /// NM_030964 | *SPRY4* | | | | *sprouty homolog 4 (Drosophila)* | 1.17E-03 | 2.99 |
| NM_001150 | *ANPEP* | | | | *alanyl (membrane) aminopeptidase* | 9.14E-03 | 2.94 |
| NM_013370 /// NM_182980 /// NM_182981 | *OSGIN1* | | | | *oxidative stress induced growth inhibitor 1* | 5.58E-05 | 2.92 |
| NM_001011649 /// NM_001272039 /// NM_018249 /// NR_073554 /// NR_073555 /// NR_073556 | *CDK5RAP2* | | | | *CDK5 regulatory subunit associated protein 2* | 2.96E-02 | 2.90 |
| NM_001145770 /// NM_001145771 /// NM_001145772 /// NM_001145773 /// NM_001145774 | | *GPR56* | | | *G protein-coupled receptor 56* | 3.20E-02 | 2.84 |
| NM_000582 /// NM_001040058 /// NM_001040060 /// NM_001251829 /// NM_001251830 | *SPP1* | | | | *secreted phosphoprotein 1* | 4.36E-02 | 2.82 |
| NM_001004417 /// NM_001004421 /// NM_001004422 /// NM_052905 | *FMNL2* | | | | *formin-like 2* | 1.46E-02 | 2.82 |
| NM_001394 /// NM_057158 | *DUSP4* | | | | *dual specificity phosphatase 4* | 2.88E-02 | 2.81 |
| NM_024769 | *CLMP* | | | | *CXADR-like membrane protein* | 1.92E-03 | 2.74 |
| NM_001015886 /// NM_003483 /// NM_003484 | *HMGA2* | | | | *high mobility group AT-hook 2* | 1.60E-03 | 2.74 |
| NM_001079533 /// NM_001079534 /// NM_001079535 /// NM_030594 | *CPEB1* | | | | *cytoplasmic polyadenylation element binding protein 1* | 2.50E-05 | 2.70 |
| NM_016848 | *SHC3* | | | | *SHC (Src homology 2 domain containing) transforming protein 3* | 3.73E-02 | 2.70 |
| NM_001146261 /// NM_001146262 /// NM_001146264 /// NM_001256006 /// NM_153262 | *SYT14* | | | | *synaptotagmin XIV* | 4.24E-03 | 2.70 |
| NM_152495 | *CNIH3* | | | | *cornichon homolog 3 (Drosophila)* | 2.42E-02 | 2.69 |
| NM_138444 | *KCTD12* | | | | *potassium channel tetramerisation domain containing 12* | 2.98E-02 | 2.68 |
| NM_025195 | *TRIB1* | | | | *tribbles homolog 1 (Drosophila)* | 3.69E-03 | 2.67 |
| NM_001024372 /// NM_024812 | *BAALC* | | | | *brain and acute leukemia, cytoplasmic* | 3.70E-02 | 2.66 |
| NM_054027 | *ANKH* | | | | *ankylosis, progressive homolog (mouse)* | 1.10E-03 | 2.63 |
| NM_014951 /// NM_199450 /// NM_199451 /// NM_199452 | *ZNF365* | | | | *zinc finger protein 365* | 5.70E-03 | 2.59 |
| NM_001001786 | *BLID* | | | | *BH3-like motif containing, cell death inducer* | 2.64E-02 | 2.58 |
| NM_001008781 | *FAT3* | | | | *FAT atypical cadherin 3* | 1.99E-02 | 2.57 |
| NM_000376 /// NM_001017535 /// NM_001017536 | *VDR* | | | | *vitamin D (1,25- dihydroxyvitamin D3) receptor* | 2.60E-02 | 2.54 |
| NM_001014797 /// NM_001161352 /// NM_001161353 /// NM_001271518 /// NM_001271519 | *KCNMA1* | | | | *potassium large conductance calcium-activated channel, subfamily M, alpha member 1* | 1.08E-03 | 2.53 |
| NM_002395 | *ME1* | | | | *malic enzyme 1, NADP(+)-dependent, cytosolic* | 2.06E-02 | 2.52 |
| NM_058238 | *WNT7B* | | | | *wingless-type MMTV integration site family, member 7B* | 1.70E-02 | 2.52 |
| NM_138809 | *CMBL* | | | | *carboxymethylenebutenolidase homolog (Pseudomonas)* | 1.48E-02 | 2.52 |
| NM_178507 | *OAF* | | | | *OAF homolog (Drosophila)* | 2.36E-02 | 2.51 |
| NM_001243797 /// NM_001243798 /// NM_001243799 /// NM_006022 /// NM_183422 | *TSC22D1* | | | | *TSC22 domain family, member 1* | 9.65E-03 | 2.50 |
| NM_001163147 /// NM_001163148 /// NM_001163149 /// NM_001163150 /// NM_001163151 | *ETV1* | | | | *ets variant 1* | 6.55E-03 | 2.47 |
| NM_006417 | *IFI44* | | | | *interferon-induced protein 44* | 5.70E-04 | 2.46 |
| NM_017633 | *FAM46A* | | | | *family with sequence similarity 46, member A* | 1.29E-02 | 2.44 |
| NM_001080470 | *ZNF697* | | | | *zinc finger protein 697* | 1.05E-02 | 2.44 |
| NM_005110 | *GFPT2* | | | | *glutamine-fructose-6-phosphate transaminase 2* | 2.02E-02 | 2.42 |
| NM_001145336 /// NM_001145337 /// NM_001145339 /// NM_001145340 /// NM_001278462 | *MDM2* | | | | *MDM2 oncogene, E3 ubiquitin protein ligase* | 1.73E-02 | 2.40 |
| NM_001271420 /// NM_003295 /// NM_017775 | *TPT1 /// TTC19* | | | | *tumor protein, translationally-controlled 1 /// tetratricopeptide repeat domain 19* | 3.41E-03 | 2.38 |
| NM_030938 /// NR_029493 | *MIR21 /// VMP1* | | | | *microRNA 21 /// vacuole membrane protein 1* | 1.48E-02 | 2.38 |
| NM_001025366 /// NM_001025367 /// NM_001025368 /// NM_001025369 /// NM_001025370 | *VEGFA* | | | | *vascular endothelial growth factor A* | 4.61E-03 | 2.36 |
| NM_001130040 /// NM_001130041 /// NM_001202859 /// NM_003029 /// NM_183001 | *SHC1* | | | | *SHC (Src homology 2 domain containing) transforming protein 1* | 4.66E-02 | 2.36 |
| NM_000688 /// NM_199166 | *ALAS1* | | | | *aminolevulinate, delta-, synthase 1* | 1.49E-03 | 2.34 |
| NM_006818 | *MLLT11* | | | | *myeloid/lymphoid or mixed-lineage leukemia (trithorax homolog, Drosophila); translocate* | 4.01E-02 | 2.32 |
| NM_000147 | *FUCA1* | | | | *fucosidase, alpha-L- 1, tissue* | 1.64E-02 | 2.31 |
| NM_001166055 /// NM_001256283 /// NM_001957 /// NR_045958 | *EDNRA* | | | | *endothelin receptor type A* | 1.96E-02 | 2.30 |
| NM_001163 | *APBA1* | | | | *amyloid beta (A4) precursor protein-binding, family A, member 1* | 4.50E-02 | 2.29 |
| NM_001267036 /// NM_001267037 /// NM_012244 /// NM_182728 /// NR_049767 | *SLC7A8* | | | | *solute carrier family 7 (amino acid transporter light chain, L system), member 8* | 9.08E-03 | 2.29 |
| NM_012417 /// NM_181671 | *PITPNC1* | | | | *phosphatidylinositol transfer protein, cytoplasmic 1* | 3.56E-02 | 2.28 |
| NM_000907 /// NM_003995 | *NPR2* | | | | *natriuretic peptide receptor B/guanylate cyclase B (atrionatriuretic peptide receptor B* | 2.67E-03 | 2.28 |
| NM_003702 /// NM_170587 | *RGS20* | | | | *regulator of G-protein signaling 20* | 4.45E-02 | 2.28 |
| NM_014278 | *HSPA4L* | | | | *heat shock 70kDa protein 4-like* | 4.06E-02 | 2.27 |
| NM_032947 | *SMIM3* | | | | *small integral membrane protein 3* | 3.42E-02 | 2.27 |
| NM_004073 | *PLK3* | | | | *polo-like kinase 3* | 1.10E-02 | 2.26 |
| NM_006254 /// NM_212539 | *PRKCD* | | | | *protein kinase C, delta* | 2.73E-03 | 2.25 |
| NM_001177676 /// NM_003485 | *GPR68* | | | | *G protein-coupled receptor 68* | 1.32E-05 | 2.24 |
| NM_012449 | *STEAP1* | | | | *six transmembrane epithelial antigen of the prostate 1* | 3.13E-02 | 2.23 |
| NM_014689 /// NM_017718 | *DOCK10* | | | | *dedicator of cytokinesis 10* | 2.36E-03 | 2.23 |
| NM_000903 /// NM_001025433 /// NM_001025434 | *NQO1* | | | | *NAD(P)H dehydrogenase, quinone 1* | 2.59E-02 | 2.22 |
| NM_001195797 /// NM_015364 | *LY96* | | | | *lymphocyte antigen 96* | 4.28E-02 | 2.22 |
| NM_007193 | *ANXA10* | | | | *annexin A10* | 2.47E-02 | 2.21 |
| NM_001260511 /// NM_001260512 /// NM_001267598 /// NM_020300 /// NM_145764 | *MGST1* | | | | *microsomal glutathione S-transferase 1* | 2.13E-03 | 2.19 |
| NM_002250 | *KCNN4* | | | | *potassium intermediate/small conductance calcium-activated channel, subfamily N, member 4* | 3.58E-03 | 2.18 |
| NM_006041 | *HS3ST3B1* | | | | *heparan sulfate (glucosamine) 3-O-sulfotransferase 3B1* | 4.57E-02 | 2.17 |
| NM_001271213 /// NM_021199 | *SQRDL* | | | | *sulfide quinone reductase-like (yeast)* | 2.17E-02 | 2.15 |
| NM_001276435 /// NM_001276436 /// NM_001276437 /// NM_001276438 /// NM_001276439 | *KCNJ15* | | | | *potassium inwardly-rectifying channel, subfamily J, member 15* | 4.62E-03 | 2.15 |
| NM_015080 /// NM_138732 /// NM_138734 | *NRXN2* | | | | *neurexin 2* | 6.84E-03 | 2.14 |
| NM_022825 /// NM_203473 /// NM_203474 /// NM_203475 /// NM_203476 | *PORCN* | | | | *porcupine homolog (Drosophila)* | 3.93E-02 | 2.13 |
| NM_003474 /// NM_021641 | *ADAM12* | | | | *ADAM metallopeptidase domain 12* | 4.83E-03 | 2.12 |
| NM_014938 | *MLXIP* | | | | *MLX interacting protein* | 9.85E-03 | 2.12 |
| NM_022361 /// NR_024539 | *POPDC3* | | | | *popeye domain containing 3* | 3.18E-03 | 2.12 |
| NM_005461 | *MAFB* | | | | *v-maf musculoaponeurotic fibrosarcoma oncogene homolog B (avian)* | 1.58E-02 | 2.12 |
| NM_013241 | *FHOD1* | | | | *formin homology 2 domain containing 1* | 9.22E-03 | 2.11 |
| NM_006933 | *SLC5A3* | | | | *solute carrier family 5 (sodium/myo-inositol cotransporter), member 3* | 3.42E-02 | 2.11 |
| NM_001195001 /// NM_005704 /// NM_133177 /// NM_133178 | *PTPRU* | | | | *protein tyrosine phosphatase, receptor type, U* | 5.19E-04 | 2.09 |
| NM_004267 | *CHST2* | | | | *carbohydrate (N-acetylglucosamine-6-O) sulfotransferase 2* | 1.41E-02 | 2.08 |
| NM_001135604 /// NM_007036 | *ESM1* | | | | *endothelial cell-specific molecule 1* | 1.55E-02 | 2.08 |
| NM_001142601 /// NM_001142602 /// NM_021972 /// NM_182965 | *SPHK1* | | | | *sphingosine kinase 1* | 3.45E-03 | 2.08 |
| NM_000123 | *ERCC5* | | | | *excision repair cross-complementation group 5* | 1.17E-02 | 2.07 |
| NM_000402 /// NM_001042351 | *G6PD* | | | | *glucose-6-phosphate dehydrogenase* | 2.38E-02 | 2.07 |
| NM_001103184 /// NM_001277313 /// NM_001277314 /// NM_198500 | *FMN1* | | | | *formin 1* | 3.11E-02 | 2.06 |
| NM_007011 /// NM_152924 | *ABHD2* | | | | *abhydrolase domain containing 2* | 4.81E-03 | 2.05 |
| NM_080725 | *SRXN1* | | | | *sulfiredoxin 1* | 3.36E-03 | 2.05 |
| NM_001146108 /// NM_001146109 /// NM_012212 | *PTGR1* | | | | *prostaglandin reductase 1* | 3.03E-03 | 2.04 |
| NM_001024628 /// NM_001024629 /// NM_001244972 /// NM_001244973 /// NM_003873 | *NRP1* | | | | *neuropilin 1* | 4.15E-02 | 2.03 |
| NM_006454 /// NR_039964 | *MIR4800 /// MXD4* | | | | *microRNA 4800 /// MAX dimerization protein 4* | 4.02E-02 | 2.03 |
| NM_000434 | *NEU1* | | | | *sialidase 1 (lysosomal sialidase)* | 3.76E-02 | 2.03 |
| NM_004567 | *PFKFB4* | | | | *6-phosphofructo-2-kinase/fructose-2,6-biphosphatase 4* | 7.41E-03 | 2.02 |
| NM_203371 | *FIBIN* | | | | *fin bud initiation factor homolog (zebrafish)* | 2.61E-02 | 2.02 |
| NM_001161841 /// NM_018837 /// NM_198596 | *SULF2* | | | | *sulfatase 2* | 1.41E-02 | 2.02 |
| NM_005968 /// NM_031203 | *HNRNPM* | | | | *heterogeneous nuclear ribonucleoprotein M* | 4.29E-02 | 2.02 |
| NM_001113239 /// NM_022740 | *HIPK2* | | | | *homeodomain interacting protein kinase 2* | 2.51E-03 | 2.02 |
| NM_032412 | *CYSTM1* | | | | *cysteine-rich transmembrane module containing 1* | 2.23E-02 | 2.01 |
| NM_002061 | *GCLM* | | | | *glutamate-cysteine ligase, modifier subunit* | 2.07E-02 | 2.01 |
| NM_015550 /// NM_145320 /// NM_145321 /// NM_145322 /// NM_145323 /// NM_145324 | *OSBPL3* | | | | *oxysterol binding protein-like 3* | 2.99E-02 | 2.00 |
| NM_003872 /// NM_018534 /// NM_201264 /// NM_201266 /// NM_201267 /// NM_201279 | *NRP2* | | | | *neuropilin 2* | 3. 87E-02 | 2.00 |
| NM_001258012 /// NM_001258013 /// NM_001258014 /// NM_001258015 /// NM_001258016 | *FDXR* | | | | *ferredoxin reductase* | 6.92E-03 | 2.00 |
| NM_033280 | *SEC11C* | | | | *SEC11 homolog C (S. cerevisiae)* | 3.55E-02 | 2.00 |
| NM_005027 /// NM_006332 /// NR_073517 | *IFI30 /// PIK3R2* | | | | *interferon, gamma-inducible protein 30 /// phosphoinositide-3-kinase, regulatory subunit 2 (beta)* | 4.45E-03 | 1.99 |
| NM_152594 | *SPRED1* | | | | *sprouty-related, EVH1 domain containing 1* | 8.88E-03 | 1.98 |
| NM_001225 /// NM_033306 /// NM_033307 | *CASP4* | | | | *caspase 4, apoptosis-related cysteine peptidase* | 3.59E-02 | 1.98 |
| NM_001080538 /// NM_020299 | *AKR1B10 /// AKR1B15* | | | | *aldo-keto reductase family 1, member B10 (aldose reductase) /// aldo-keto reductase family 1, member B15* | 9.43E-03 | 1.98 |
| NM_001135729 /// NM_001135730 /// NM_001135732 /// NM_005488 | *TOM1* | | | | *target of myb1 (chicken)* | 2.13E-02 | 1.97 |
| NM_003596 | *TPST1* | | | | *tyrosylprotein sulfotransferase 1* | 1.91E-02 | 1.97 |
| NM_016029 | *DHRS7* | | | | *dehydrogenase/reductase (SDR family) member 7* | 1.42E-02 | 1.96 |
| NM_198147 | *ABHD15* | | | | *abhydrolase domain containing 15* | 1.05E-02 | 1.96 |
| NM_001831 /// NR_038335 /// NR_045494 | *CLU* | | | | *clusterin* | 2.51E-02 | 1.95 |
| NM_012434 | *SLC17A5* | | | | *solute carrier family 17 (anion/sugar transporter), member 5* | 2.12E-02 | 1.95 |
| NM_030775 /// NM_032642 | *WNT5B* | | | | *wingless-type MMTV integration site family, member 5B* | 2.05E-02 | 1.95 |
| NM_002569 | *FURIN* | | | | *furin (paired basic amino acid cleaving enzyme)* | 2.53E-02 | 1.94 |
| NM_016029 | *DHRS7* | | | | *dehydrogenase/reductase (SDR family) member 7* | 6.69E-03 | 1.93 |
| NM_001134367 /// NM_001134368 /// NM_003043 | *SLC6A6* | | | | *solute carrier family 6 (neurotransmitter transporter, taurine), member 6* | 3.09E-02 | 1.93 |
| NM_006823 /// NM_181839 | *PKIA* | | | | *protein kinase (cAMP-dependent, catalytic) inhibitor alpha* | 3.02E-02 | 1.93 |
| NM_004995 | *MMP14* | | | | *matrix metallopeptidase 14 (membrane-inserted)* | 6.01E-03 | 1.92 |
| NM_001135648 /// NM_002844 | *PTPRK* | | | | *protein tyrosine phosphatase, receptor type, K* | 3.24E-03 | 1.91 |
| NM_001039574 /// NM_004978 /// NM_153763 /// NR_036437 | *KCNC4* | | | | *potassium voltage-gated channel, Shaw-related subfamily, member 4* | 2.95E-02 | 1.91 |
| NM_001166693 /// NM_005935 | *AFF1* | | | | *AF4/FMR2 family, member 1* | 3.36E-02 | 1.89 |
| NM_175918 | *CRIPAK* | | | | *cysteine-rich PAK1 inhibitor* | 4.39E-02 | 1.89 |
| NM_004170 | *SLC1A1* | | | | *solute carrier family 1 (neuronal/epithelial high affinity glutamate transporter, system Xag), member 1* | 2.32E-02 | 1.88 |
| NM_015288 | *PHF15* | | | | *PHD finger protein 15* | 3.56E-03 | 1.88 |
| NM_001199933 /// NM_001199934 /// NM_014454 | *SESN1* | | | | *sestrin 1* | 8.10E-03 | 1.87 |
| NM_003174 /// NM_021738 | *SVIL* | | | | *supervillin* | 2.56E-02 | 1.87 |
| NM_001178005 /// NM_017614 | *BHMT2* | | | | *betaine--homocysteine S-methyltransferase 2* | 2.00E-03 | 1.86 |
| NM_001198696 /// NM_032797 | *AIFM2* | | | | *apoptosis-inducing factor, mitochondrion-associated, 2* | 1.43E-02 | 1.86 |
| NM_001135216 /// NM_001243241 /// NM_001243242 /// NM_001243243 | *ZSCAN31* | | | | *zinc finger and SCAN domain containing 31* | 1.95E-02 | 1.86 |
| NM_032824 | *TMEM87B* | | | | *transmembrane protein 87B* | 3.35E-03 | 1.86 |
| NM_022772 | *EPS8L2* | | | | *EPS8-like 2* | 4.00E-02 | 1.86 |
| NM_001142391 /// NM_001142392 /// NM_019848 | *SLC10A3* | | | | *solute carrier family 10 (sodium/bile acid cotransporter family), member 3* | 1.07E-02 | 1.86 |
| NM_020375 | *C12orf5* | | | | *chromosome 12 open reading frame 5* | 3.01E-02 | 1.85 |
| NM_005746 /// NM_182790 | *NAMPT* | | | | *nicotinamide phosphoribosyltransferase* | 4.14E-02 | 1.85 |
| NM_001127453 /// NM_001127454 /// NM_004403 | *DFNA5* | | | | *deafness, autosomal dominant 5* | 3.56E-02 | 1.85 |
| NM_001098520 /// NM_001098521 /// NM_001098522 /// NM_001098523 /// NM_006410 | *HTATIP2* | | | | *HIV-1 Tat interactive protein 2, 30kDa* | 3.24E-02 | 1.84 |
| NM_001009607 /// NM_001080530 /// NM_032167 | *SNX29* | | | | *sorting nexin 29* | 2.94E-02 | 1.84 |
| NM_014600 | *EHD3* | | | | *EH-domain containing 3* | 2.44E-02 | 1.84 |
| NM_174921 | *SMIM14* | | | | *small integral membrane protein 14* | 2.77E-02 | 1.84 |
| NM_005996 /// NM_016569 | *TBX3* | | | | *T-box 3* | 5.65E-03 | 1.84 |
| NM_002872 | *RAC2* | | | | *ras-related C3 botulinum toxin substrate 2 (rho family, small GTP binding protein Rac2)* | 9.90E-05 | 1.83 |
| NM_005415 | *SLC20A1* | | | | *solute carrier family 20 (phosphate transporter), member 1* | 8.17E-03 | 1.83 |
| NM_000602 | *SERPINE1* | | | | *serpin peptidase inhibitor, clade E (nexin, plasminogen activator inhibitor type 1), member 1* | 4.47E-02 | 1.83 |
| NM_015221 | *DNMBP* | | | | *dynamin binding protein* | 1.64E-02 | 1.82 |
| NM_001080976 /// NM_013352 | *DSE* | | | | *dermatan sulfate epimerase* | 3.06E-04 | 1.82 |
| NM_001064 /// NM_001135055 /// NM_001135056 /// NM_001258028 | *TKT* | | | | *transketolase* | 3.50E-02 | 1.82 |
| NM_015348 | *TMEM131* | | | | *transmembrane protein 131* | 4.92E-03 | 1.82 |
| NM_015009 | *PDZRN3* | | | | *PDZ domain containing ring finger 3* | 2.57E-02 | 1.82 |
| NM_001142401 /// NM_001142402 /// NM_001142403 /// NM_001142404 /// NM_006016 | *CD164* | | | | *CD164 molecule, sialomucin* | 4.93E-02 | 1.82 |
| NM_003840 | *TNFRSF10D* | | | | *tumor necrosis factor receptor superfamily, member 10d, decoy with truncated death domain* | 2.32E-02 | 1.81 |
| NM_003749 | *IRS2* | | | | *insulin receptor substrate 2* | 3.05E-02 | 1.81 |
| NM_006876 | *B3GNT1* | | | | *UDP-GlcNAc: betaGal beta-1,3-N-acetylglucosaminyltransferase 1* | 1.11E-02 | 1.81 |
| NM_001267040 /// NM_004973 | *JARID2* | | | | *jumonji, AT rich interactive domain 2* | 9.65E-03 | 1.80 |
| NM_001080855 /// NM_001243756 /// NM_002859 /// NM_025157 | *PXN* | | | | *paxillin* | 4.20E-02 | 1.80 |
| NM_000382 /// NM_001031806 | *ALDH3A2* | | | | *aldehyde dehydrogenase 3 family, member A2* | 8.94E-03 | 1.80 |
| NM_022477 /// NM_032013 /// NR_038370 | *NDRG3* | | | | *NDRG family member 3* | 3.93E-03 | 1.80 |
| NM_005438 | *FOSL1* | | | | *FOS-like antigen 1* | 1.98E-02 | 1.79 |
| NR_002956 | *SNORA14B* | | | | *small nucleolar RNA, H/ACA box 14B* | 4.34E-02 | 1.78 |
| NM_002131 /// NM_145899 /// NM_145901 /// NM_145902 /// NM_145903 /// NM_145904 | *HMGA1* | | | | *high mobility group AT-hook 1* | 1.20E-02 | 1.78 |
| NR_002912 | *SNORA67* | | | | *small nucleolar RNA, H/ACA box 67* | 1.84E-02 | 1.78 |
| NM_001167903 /// NM_022736 | *MFSD1* | | | | *major facilitator superfamily domain containing 1* | 4.94E-02 | 1.78 |
| NM_001001342 /// NM_173809 /// NR_046296 /// NR_046314 /// NR_046315 | *BLOC1S2* | | | | *biogenesis of lysosomal organelles complex-1, subunit 2* | 2.34E-02 | 1.78 |
| NM_025191 | *EDEM3* | | | | *ER degradation enhancer, mannosidase alpha-like 3* | 2.65E-02 | 1.78 |
| NM_015299 | *KHNYN* | | | | *KH and NYN domain containing* | 9.03E-03 | 1.78 |
| NM_005194 | *CEBPB* | | | | *CCAAT/enhancer binding protein (C/EBP), beta* | 9.74E-03 | 1.78 |
| NM_001030059 | *PPAPDC1A* | | | | *phosphatidic acid phosphatase type 2 domain containing 1A* | 3.93E-03 | 1.78 |
| NR_002819 | *MALAT1* | | | | *metastasis associated lung adenocarcinoma transcript 1 (non-protein coding)* | 4.24E-02 | 1.78 |
| NM_001256024 /// NM_001256025 /// NM_001256026 /// NM_001256027 /// NM_021226 | *ARHGAP22* | | | | *Rho GTPase activating protein 22* | 2.75E-02 | 1.77 |
| NM_001039457 /// NM_004047 | *ATP6V0B* | | | | *ATPase, H+ transporting, lysosomal 21kDa, V0 subunit b* | 1.71E-02 | 1.77 |
| NM_153367 | *ZCCHC24* | | | | *zinc finger, CCHC domain containing 24* | 1.26E-02 | 1.77 |
| NM_004760 | *STK17A* | | | | *serine/threonine kinase 17a* | 2.46E-02 | 1.77 |
| NM_001178088 /// NM_003898 | *SYNJ2* | | | | *synaptojanin 2* | 2.12E-02 | 1.77 |
| NM_004148 | *NINJ1* | | | | *ninjurin 1* | 4.99E-02 | 1.77 |
| NM_003144 | *SSR1* | | | | *signal sequence receptor, alpha* | 3.45E-02 | 1.77 |
| NM_001165903 /// NM_004603 | *STX1A* | | | | *syntaxin 1A (brain)* | 2.77E-02 | 1.77 |
| NR_026943 | *LOC642852* | | | | *uncharacterized LOC642852* | 1.34E-02 | 1.77 |
| NM_001257971 /// NM_001257972 /// NM_001257973 /// NM_001912 /// NM_145918 | *CTSL1* | | | | *cathepsin L1* | 3.85E-02 | 1.76 |
| NM_001164664 /// NM_015183 /// NM_198828 | *MAST4* | | | | *microtubule associated serine/threonine kinase family member 4* | 4.63E-02 | 1.76 |
| NM_005228 /// NM_201282 /// NM_201283 /// NM_201284 | *EGFR* | | | | *epidermal growth factor receptor* | 2.41E-02 | 1.76 |
| NM_001200001 /// NM_024408 | *NOTCH2* | | | | *notch 2* | 1.58E-02 | 1.76 |
| NM_005952 | *MT1X* | | | | *metallothionein 1X* | 1.01E-02 | 1.75 |
| NM_020448 | *NIPAL3* | | | | *NIPA-like domain containing 3* | 4.91E-02 | 1.75 |
| NM_000959 /// NM_001039585 | *PTGFR* | | | | *prostaglandin F receptor (FP)* | 1.99E-02 | 1.75 |
| NM_001145769 /// NM_004628 | *XPC* | | | | *xeroderma pigmentosum, complementation group C* | 3.73E-02 | 1.75 |
| NM_000043 /// NM_152871 /// NM_152872 /// NM_152873 /// NM_152874 /// NM_152875 | *FAS* | | | | *Fas cell surface death receptor* | 8.08E-04 | 1.74 |
| NM_001005336 /// NM_004408 | *DNM1* | | | | *dynamin 1* | 5.98E-04 | 1.74 |
| NM_001270526 /// NM_001270527 /// NM_004099 /// NM_198194 /// NR_073037 | *STOM* | | | | *stomatin* | 2.66E-02 | 1.74 |
| NM_001242854 /// NM_001242855 /// NM_001242856 /// NM_012402 | *ARFIP2* | | | | *ADP-ribosylation factor interacting protein 2* | 2.39E-02 | 1.73 |
| NM_001099784 /// NM_001271651 /// NM_019085 /// NR_073401 | *FBXL19* | | | | *F-box and leucine-rich repeat protein 19* | 1.55E-02 | 1.73 |
| NM_178148 /// NR_039790 | *MIR4647 /// SLC35B2* | | | | *microRNA 4647 /// solute carrier family 35, member B2* | 2.85E-02 | 1.73 |
| NM_203403 | *LURAP1L* | | | | *leucine rich adaptor protein 1-like* | 9.22E-03 | 1.72 |
| NM_001040194 /// NM_001040195 /// NM_001040196 /// NM_001040197 /// NM_020350 | *AGTRAP* | | | | *angiotensin II receptor-associated protein* | 2.36E-02 | 1.72 |
| NM_001001928 /// NM_001001929 /// NM_001001930 /// NM_005036 /// NM_032644 | *PPARA* | | | | *peroxisome proliferator-activated receptor alpha* | 1.92E-02 | 1.72 |
| NM_012334 | *MYO10* | | | | *myosin X* | 3.90E-02 | 1.72 |
| NM_002636 /// NM_024165 /// NR_027692 | *PHF1* | | | | *PHD finger protein 1* | 1.61E-03 | 1.72 |
| NM_000228 /// NM_001017402 /// NM_001127641 | *LAMB3* | | | | *laminin, beta 3* | 4.73E-03 | 1.72 |
| NM_001097599 /// NM_001097600 /// NM_025246 | *SLC35G2* | | | | *solute carrier family 35, member G2* | 4.35E-02 | 1.72 |
| NM_001244705 /// NM_001244706 /// NM_015989 | *CSAD* | | | | *cysteine sulfinic acid decarboxylase* | 3.39E-02 | 1.72 |
| NM_003842 /// NM_147187 | *TNFRSF10B* | | | | *tumor necrosis factor receptor superfamily, member 10b* | 3.91E-02 | 1.71 |
| NM_020412 | *CHMP1B* | | | | *charged multivesicular body protein 1B* | 2.91E-02 | 1.71 |
| NM_205855 | *FAM180A* | | | | *family with sequence similarity 180, member A* | 2.14E-02 | 1.71 |
| NM_001136528 /// NM_001136530 /// NM_006216 | *SERPINE2* | | | | *serpin peptidase inhibitor, clade E (nexin, plasminogen activator inhibitor type 1), member 2* | 1.88E-02 | 1.71 |
| NM_016230 | *CYB5R4* | | | | *cytochrome b5 reductase 4* | 1.11E-02 | 1.71 |
| NM_001127383 /// NM_001256909 /// NM_024843 | *CYBRD1* | | | | *cytochrome b reductase 1* | 1.30E-02 | 1.70 |
| NM_002032 | *FTH1* | | | | *ferritin, heavy polypeptide 1* | 3.88E-02 | 1.70 |
| NM_001037131 /// NM_001244888 /// NM_014914 | *AGAP1* | | | | *ArfGAP with GTPase domain, ankyrin repeat and PH domain 1* | 6.86E-04 | 1.70 |
| NM_020381 | *PDSS2* | | | | *prenyl (decaprenyl) diphosphate synthase, subunit 2* | 3.59E-02 | 1.70 |
| NM_002737 | *PRKCA* | | | | *protein kinase C, alpha* | 1.05E-02 | 1.70 |
| NM_006828 /// NM_022091 | *ASCC3* | | | | *activating signal cointegrator 1 complex subunit 3* | 7.20E-04 | 1.70 |
| NM_014331 | *SLC7A11* | | | | *solute carrier family 7 (anionic amino acid transporter light chain, xc- system), member 11* | 4.08E-02 | 1.69 |
| NM_022818 | *MAP1LC3B* | | | | *microtubule-associated protein 1 light chain 3 beta* | 4.09E-02 | 1.69 |
| NM_001191322 /// NM_001191323 /// NM_013372 | *GREM1* | | | | *gremlin 1, DAN family BMP antagonist* | 1.39E-02 | 1.69 |
| NM_000690 /// NM_001204889 | *ALDH2* | | | | *aldehyde dehydrogenase 2 family (mitochondrial)* | 4.12E-02 | 1.69 |
| NM_003463 | *PTP4A1* | | | | *protein tyrosine phosphatase type IVA, member 1* | 8.44E-03 | 1.69 |
| NM_001127891 /// NM_004530 | *MMP2* | | | | *matrix metallopeptidase 2 (gelatinase A, 72kDa gelatinase, 72kDa type IV collagenase)* | 3.18E-02 | 1.68 |
| NM_001037537 /// NM_006214 | *PHYH* | | | | *phytanoyl-CoA 2-hydroxylase* | 1.79E-02 | 1.68 |
| NM_001005743 /// NM_001005744 /// NM_001005745 /// NM_003744 | *NUMB* | | | | *numb homolog (Drosophila)* | 3.06E-02 | 1.68 |
| NM_024838 | *THNSL1* | | | | *threonine synthase-like 1 (S. cerevisiae)* | 1.96E-02 | 1.68 |
| NM_001145001 /// NM_001166167 /// NM_001166168 /// NM_001166169 /// NM_001166170 | *NEK6* | | | | *NIMA-related kinase 6* | 3.68E-03 | 1.67 |
| NM_145172 | *WDR63* | | | | *WD repeat domain 63* | 2.25E-02 | 1.67 |
| NM_003821 | *RIPK2* | | | | *receptor-interacting serine-threonine kinase 2* | 3.89E-03 | 1.67 |
| NM_001023567 /// NM_181077 /// NR_027409 /// NR_027410 | *GOLGA8A /// GOLGA8B* | | | | *golgin A8 family, member A /// golgin A8 family, member B* | 1.44E-02 | 1.67 |
| NM_001009955 /// NM_018070 /// NM_145716 | *SSBP3* | | | | *single stranded DNA binding protein 3* | 1.35E-02 | 1.67 |
| NM_001164615 /// NM_001164616 /// NM_003506 | *FZD6* | | | | *frizzled family receptor 6* | 2.53E-02 | 1.67 |
| NM_001031716 /// NM_001254736 /// NM_022837 /// NR_045622 /// NR_045623 | *NABP1* | | | | *nucleic acid binding protein 1* | 1.27E-02 | 1.67 |
| NM_001039664 /// NM_003790 /// NM_148965 /// NM_148966 /// NM_148967 /// NM_148968 | *TNFRSF25* | | | | *tumor necrosis factor receptor superfamily, member 25* | 4.66E-02 | 1.67 |
| NM_001006940 /// NM_001006941 /// NM_005787 /// NR_024533 /// NR_024534 | *ALG3* | | | | *ALG3, alpha-1,3- mannosyltransferase* | 8.30E-04 | 1.67 |
| NM_004798 | *KIF3B* | | | | *kinesin family member 3B* | 1.20E-02 | 1.66 |
| NM_001191002 /// NM_001191003 /// NM_004832 | *GSTO1* | | | | *glutathione S-transferase omega 1* | 2.13E-02 | 1.66 |
| NM_001191 /// NM_138578 | *BCL2L1* | | | | *BCL2-like 1* | 3.90E-02 | 1.66 |
| NM_022726 | *ELOVL4* | | | | *ELOVL fatty acid elongase 4* | 2.28E-02 | 1.66 |
| NM_015144 | *ZCCHC14* | | | | *zinc finger, CCHC domain containing 14* | 3.02E-02 | 1.66 |
| NM_030571 | *NDFIP1* | | | | *Nedd4 family interacting protein 1* | 9.19E-03 | 1.65 |
| NM_203434 | *IER5L* | | | | *immediate early response 5-like* | 4.81E-02 | 1.65 |
| NM_001135662 /// NM_001135663 /// NM_001135664 /// NM_003929 | *RAB29* | | | | *RAB29, member RAS oncogene* | 1.33E-02 | 1.65 |
| NM_005429 | *VEGFC* | | | | *vascular endothelial growth factor C* | 2.47E-02 | 1.65 |
| NM_001256647 /// NM_007121 | *NR1H2* | | | | *nuclear receptor subfamily 1, group H, member 2* | 2.42E-02 | 1.65 |
| NM_024496 | *IRF2BPL* | | | | *interferon regulatory factor 2 binding protein-like* | 2.75E-02 | 1.65 |
| NM_001007254 /// NM_001695 | *ATP6V1C1* | | | | *ATPase, H+ transporting, lysosomal 42kDa, V1 subunit C1* | 3.28E-03 | 1.65 |
| NM_001184700 /// NM_001184701 /// NM_003359 | *UGDH* | | | | *UDP-glucose 6-dehydrogenase* | 1.29E-02 | 1.64 |
| NM_004531 /// NM_176806 | *MOCS2* | | | | *molybdenum cofactor synthesis 2* | 4.55E-02 | 1.64 |
| NM_001164410 /// NM_004935 | *CDK5* | | | | *cyclin-dependent kinase 5* | 2.77E-03 | 1.64 |
| NM_016113 | *TRPV2* | | | | *transient receptor potential cation channel, subfamily V, member 2* | 4.57E-02 | 1.64 |
| NM_002970 /// NR_027783 | *SAT1* | | | | *spermidine/spermine N1-acetyltransferase 1* | 3.20E-02 | 1.64 |
| NM_012098 | *ANGPTL2* | | | | *angiopoietin-like 2* | 3.68E-04 | 1.64 |
| NM_005228 /// NM_201282 /// NM_201283 /// NM_201284 | *EGFR* | | | | *epidermal growth factor receptor* | 3.34E-02 | 1.64 |
| NM_032536 | *NTNG2* | | | | *netrin G2* | 2.98E-02 | 1.64 |
| NM_015946 | *PELO* | | | | *pelota homolog (Drosophila)* | 1.85E-03 | 1.64 |
| NM_001242559 /// NM_001242560 /// NM_004834 /// NM_145686 /// NM_145687 | *MAP4K4* | | | | *mitogen-activated protein kinase kinase kinase kinase 4* | 3.18E-02 | 1.64 |
| NM_001130841 /// NM_003729 | *RTCA* | | | | *RNA 3'-terminal phosphate cyclase* | 4.95E-02 | 1.63 |
| NM_005570 | *LMAN1* | | | | *lectin, mannose-binding, 1* | 2.42E-02 | 1.63 |
| NM_001012505 /// NM_001244808 /// NM_001244810 /// NM_001244812 /// NM_001244813 | *FOXP1* | | | | *forkhead box P1* | 2.41E-02 | 1.63 |
| NM_130807 | *MOB3A* | | | | *MOB kinase activator 3A* | 1.09E-02 | 1.63 |
| NM_001202513 /// NM_001202514 /// NM_002357 | *MXD1* | | | | *MAX dimerization protein 1* | 1.34E-02 | 1.63 |
| NM_001145155 /// NM_001145156 /// NM_001145157 /// NM_021005 | *NR2F2* | | | | *nuclear receptor subfamily 2, group F, member 2* | 2.51E-02 | 1.63 |
| NM_015089 | *CUL9* | | | | *cullin 9* | 2.90E-02 | 1.62 |
| NM_002675 /// NM_033238 /// NM_033239 /// NM_033240 /// NM_033244 /// NM_033246 | *PML* | | | | *promyelocytic leukemia* | 5.48E-03 | 1.62 |
| NM_001033026 /// NM_033420 | *TMEM259* | | | | *transmembrane protein 259* | 4.07E-02 | 1.62 |
| NM_152734 | *C6orf89* | | | | *chromosome 6 open reading frame 89* | 2.31E-03 | 1.62 |
| NM_001153 | *ANXA4* | | | | *annexin A4* | 6.07E-03 | 1.62 |
| NM_004181 | *UCHL1* | | | | *ubiquitin carboxyl-terminal esterase L1 (ubiquitin thiolesterase)* | 1.70E-03 | 1.62 |
| NM_003439 | *ZKSCAN1* | | | | *zinc finger with KRAB and SCAN domains 1* | 3.11E-02 | 1.61 |
| NM_001167738 /// NM_020443 | *NAV1* | | | | *neuron navigator 1* | 1.42E-02 | 1.61 |
| NM_003522 /// NM_003523 /// NM_003526 | *HIST1H2BC /// HIST1H2BE /// HIST1H2BF* | | | | *histone cluster 1, H2bc /// histone cluster 1, H2be /// histone cluster 1, H2bf* | 1.26E-02 | 1.61 |
| NM_005347 | *HSPA5* | | | | *heat shock 70kDa protein 5 (glucose-regulated protein, 78kDa)* | 1.26E-02 | 1.61 |
| NM_001163673 /// NM_001163809 /// NM_001163811 /// NM_152348 | *WDR81* | | | | *WD repeat domain 81* | 2.27E-02 | 1.61 |
| NM_003107 | *SOX4* | | | | *SRY (sex determining region Y)-box 4* | 3.43E-02 | 1.61 |
| NM_001320 | *CSNK2B* | | | | *casein kinase 2, beta polypeptide* | 1.60E-02 | 1.61 |
| NM_003170 | *SUPT6H* | | | | *suppressor of Ty 6 homolog (S. cerevisiae)* | 3.38E-02 | 1.60 |
| NM_007270 | *FKBP9* | | | | *FK506 binding protein 9, 63 kDa* | 1.91E-02 | 1.60 |
| NM_173557 | *RNF152* | | | | *ring finger protein 152* | 2.80E-02 | 1.60 |
| NM_001690 | *ATP6V1A* | | | | *ATPase, H+ transporting, lysosomal 70kDa, V1 subunit A* | 1.51E-02 | 1.60 |
| NM_006887 | *ZFP36L2* | | | | *ZFP36 ring finger protein-like 2* | 4.28E-02 | 1.60 |
| NM_025135 | *FHOD3* | | | | *formin homology 2 domain containing 3* | 4.30E-02 | 1.60 |
| NM_006460 | *HEXIM1* | | | | *hexamethylene bis-acetamide inducible 1* | 9.81E-03 | 1.60 |
| NM_006988 | *ADAMTS1* | | | | *ADAM metallopeptidase with thrombospondin type 1 motif, 1* | 3.81E-02 | 1.60 |
| NM_000628 | *IL10RB* | | | | *interleukin 10 receptor, beta* | 2.06E-02 | 1.59 |
| NM_018425 | *PI4K2A* | | | | *phosphatidylinositol 4-kinase type 2 alpha* | 7.84E-03 | 1.59 |
| NM_005230 | *ELK3* | | | | *ELK3, ETS-domain protein (SRF accessory protein 2)* | 2.91E-02 | 1.59 |
| NM_001024843 /// NM_001162501 /// NM_015088 | *TNRC6B* | | | | *trinucleotide repeat containing 6B* | 3.51E-02 | 1.59 |
| NM_000610 /// NM_001001389 /// NM_001001390 /// NM_001001391 /// NM_001001392 | *CD44* | | | | *CD44 molecule (Indian blood group)* | 2.19E-02 | 1.58 |
| NM_004324 /// NM_138761 /// NM_138762 /// NM_138763 /// NM_138764 /// NR_027882 | *BAX* | | | | *BCL2-associated X protein* | 4.67E-02 | 1.58 |
| NM_004090 | *DUSP3* | | | | *dual specificity phosphatase 3* | 1.38E-02 | 1.58 |
| NM_001198915 /// NM_001198916 /// NM_003622 /// NM_177444 | *PPFIBP1* | | | | *PTPRF interacting protein, binding protein 1 (liprin beta 1)* | 3.28E-02 | 1.58 |
| NM_001024847 /// NM_003242 | *TGFBR2* | | | | *transforming growth factor, beta receptor II (70/80kDa)* | 1.77E-02 | 1.58 |
| NM_014044 | *UNC50* | | | | *unc-50 homolog (C. elegans)* | 9.91E-03 | 1.58 |
| NM_001040059 /// NM_001251 | *CD68* | | | | *CD68 molecule* | 1.32E-02 | 1.58 |
| NM_005715 | *UST* | | | | *uronyl-2-sulfotransferase* | 4.84E-02 | 1.58 |
| NM_001243372 /// NM_001243374 /// NM_001829 /// NM_173872 | *CLCN3* | | | | *chloride channel, voltage-sensitive 3* | 4.39E-02 | 1.58 |
| NM_012170 /// NM_147188 /// NR_037623 | *FBXO22* | | | | *F-box protein 22* | 3.67E-04 | 1.58 |
| NM_001167 /// NM_001204401 /// NR_037916 | *XIAP* | | | | *X-linked inhibitor of apoptosis* | 7.93E-03 | 1.57 |
| NM_001267562 /// NM_001267563 /// NM_001267564 /// NM_001267565 /// NM_001267566 | *CREM* | | | | *cAMP responsive element modulator* | 4.87E-02 | 1.57 |
| NM_000146 | *FTL* | | | | *ferritin, light polypeptide* | 4.92E-02 | 1.57 |
| NM_004223 /// NM_198183 | *UBE2L6* | | | | *ubiquitin-conjugating enzyme E2L 6* | 3.30E-02 | 1.57 |
| NM_001077516 /// NM_006979 | *SLC39A7* | | | | *solute carrier family 39 (zinc transporter), member 7* | 9.87E-03 | 1.57 |
| NM_001258357 /// NM_001258358 /// NM_001258359 /// NM_002149 /// NM_134421 | *HPCAL1* | | | | *hippocalcin-like 1* | 3.67E-03 | 1.57 |
| NM_002487 | *NDN* | | | | *necdin, melanoma antigen (MAGE) family member* | 2.89E-02 | 1.57 |
| NM_001039842 | *OXLD1* | | | | *oxidoreductase-like domain containing 1* | 1.59E-02 | 1.57 |
| NM_001024666 /// NM_001184960 /// NM_031892 | *SH3KBP1* | | | | *SH3-domain kinase binding protein 1* | 2.76E-03 | 1.57 |
| NM_001190274 /// NM_012167 /// NM_018693 /// NM_025133 | *FBXO11* | | | | *F-box protein 11* | 4.14E-02 | 1.57 |
| NM_001256182 /// NM_001256183 /// NM_013275 /// NR_045839 | *ANKRD11* | | | | *ankyrin repeat domain 11* | 3.47E-02 | 1.57 |
| NM_001206802 /// NM_004881 /// NM_147184 | *TP53I3* | | | | *tumor protein p53 inducible protein 3* | 1.84E-02 | 1.56 |
| NM_001146191 /// NM_003953 /// NM_024569 | *MPZL1* | | | | *myelin protein zero-like 1* | 2.82E-02 | 1.56 |
| NM_003388 /// NM_032421 | *CLIP2* | | | | *CAP-GLY domain containing linker protein 2* | 6.65E-03 | 1.56 |
| NM_006503 /// NM_153001 | *PSMC4* | | | | *proteasome (prosome, macropain) 26S subunit, ATPase, 4* | 1.64E-02 | 1.56 |
| NM_001144822 /// NM_001779 /// NR_026665 | *CD58* | | | | *CD58 molecule* | 3.44E-02 | 1.56 |
| NM_001080519 | *BAHCC1* | | | | *BAH domain and coiled-coil containing 1* | 1.25E-02 | 1.56 |
| NM_001037553 /// NM_020132 | *AGPAT3* | | | | *1-acylglycerol-3-phosphate O-acyltransferase 3* | 4.07E-02 | 1.56 |
| NM_001258205 /// NM_145068 | *TRPV3* | | | | *transient receptor potential cation channel, subfamily V, member 3* | 3.06E-02 | 1.56 |
| NM_005953 | *MT2A* | | | | *metallothionein 2A* | 1.73E-02 | 1.56 |
| NM_000188 /// NM_033496 /// NM_033497 /// NM_033498 /// NM_033500 | *HK1* | | | | *hexokinase 1* | 6.25E-03 | 1.56 |
| NM_001008410 /// NM_018234 /// NM_182915 | *STEAP3* | | | | *STEAP family member 3, metalloreductase* | 1.32E-02 | 1.55 |
| NM_001278469 /// NM_001278470 /// NM_001278471 /// NM_001278472 /// NM_015026 | *MON2* | | | | *MON2 homolog (S. cerevisiae)* | 9.19E-03 | 1.55 |
| NM_002373 | *MAP1A* | | | | *microtubule-associated protein 1A* | 2.63E-02 | 1.55 |
| NM_003088 | *FSCN1* | | | | *fascin homolog 1, actin-bundling protein (Strongylocentrotus purpuratus)* | 2.34E-02 | 1.55 |
| NM_000122 | *ERCC3* | | | | *excision repair cross-complementing rodent repair deficiency, complementation group 3* | 4.85E-02 | 1.55 |
| NM_014445 | *SERP1* | | | | *stress-associated endoplasmic reticulum protein 1* | 1.63E-02 | 1.55 |
| NM_001115113 /// NM_025160 | *WDR26* | | | | *WD repeat domain 26* | 3.99E-02 | 1.54 |
| NM_030662 | *MAP2K2* | | | | *mitogen-activated protein kinase kinase 2* | 2.52E-02 | 1.54 |
| NM_015289 | *VPS39* | | | | *vacuolar protein sorting 39 homolog (S. cerevisiae)* | 1.87E-02 | 1.54 |
| NM_005669 | *REEP5* | | | | *receptor accessory protein 5* | 4.98E-02 | 1.54 |
| NM_001164750 /// NM_001164751 /// NM_001164752 /// NM_001164753 /// NM_001164754 | *ASPH* | | | | *aspartate beta-hydroxylase* | 2.46E-02 | 1.54 |
| NM_001244889 /// NM_170744 | *UNC5B* | | | | *unc-5 homolog B (C. elegans)* | 3.27E-02 | 1.54 |
| NM_001169106 /// NM_001169107 /// NM_015262 /// XM_003960860 | *FAM21C* | | | | *family with sequence similarity 21, member C* | 1.30E-02 | 1.54 |
| NM_005868 | *BET1* | | | | *Bet1 golgi vesicular membrane trafficking protein* | 2.34E-02 | 1.54 |
| NM_003969 | *UBE2M* | | | | *ubiquitin-conjugating enzyme E2M* | 2.24E-02 | 1.53 |
| NM_001178078 /// NM_001178079 /// NM_001178080 /// NM_001178081 /// NM_003153 | *STAT6* | | | | *signal transducer and activator of transcription 6, interleukin-4 induced* | 3.71E-02 | 1.53 |
| NM_017971 | *MRPL20* | | | | *mitochondrial ribosomal protein L20* | 4.69E-02 | 1.53 |
| NM_014983 | *HMGXB3* | | | | *HMG box domain containing 3* | 1.26E-02 | 1.53 |
| NM_032317 | *DNAJC30* | | | | *DnaJ (Hsp40) homolog, subfamily C, member 30* | 6.91E-03 | 1.53 |
| NM_000086 /// NM_001042432 | *CLN3* | | | | *ceroid-lipofuscinosis, neuronal 3* | 5.56E-03 | 1.53 |
| NM_001024736 /// NM_025240 | *CD276* | | | | *CD276 molecule* | 2.17E-02 | 1.53 |
| NM_001142448 /// NM_001142449 /// NM_001142450 /// NM_001142451 /// NM_032038 | *SPNS1* | | | | *spinster homolog 1 (Drosophila)* | 1.59E-03 | 1.53 |
| NM_002035 | *KDSR* | | | | *3-ketodihydrosphingosine reductase* | 4.44E-02 | 1.52 |
| NM_001243877 /// NM_001243885 /// NM_005749 | *TOB1* | | | | *transducer of ERBB2, 1* | 2.37E-03 | 1.52 |
| NM_001113402 /// NM_001278411 /// NM_001278412 /// NR_004854 /// NR_103522 | *AMN1* | | | | *antagonist of mitotic exit network 1 homolog (S. cerevisiae)* | 1.31E-02 | 1.52 |
| NM_001174092 /// NM_032508 | *TMEM185A* | | | | *transmembrane protein 185A* | 4.40E-02 | 1.52 |
| NM_020894 | *UVSSA* | | | | *UV-stimulated scaffold protein A* | 9.28E-03 | 1.52 |
| NM_021959 /// NM_170781 | *PPP1R11* | | | | *protein phosphatase 1, regulatory (inhibitor) subunit 11* | 4.32E-02 | 1.52 |
| NM_024963 | *FBXL18* | | | | *F-box and leucine-rich repeat protein 18* | 2.07E-02 | 1.52 |
| NM_016297 | *PCYOX1* | | | | *prenylcysteine oxidase 1* | 2.52E-02 | 1.52 |
| NM_001080391 /// NM_001206701 /// NM_001206702 /// NM_001206703 /// NM_001206704 | *SP100* | | | | *SP100 nuclear antigen*  229 | 2.46E-03 | 1.52 |
| NM_006646 | *WASF3* | | | | *WAS protein family, member 3* | 3.85E-02 | 1.52 |
| NM_032892 | *FRMD5* | | | | *FERM domain containing 5* | 3.63E-02 | 1.51 |
| NM_001199573 /// NM_006074 | *TRIM22* | | | | *tripartite motif containing 22* | 4.22E-02 | 1.51 |
| NM_001040118 /// NM_001135190 /// NM_015242 | *ARAP1* | | | | *ArfGAP with RhoGAP domain, ankyrin repeat and PH domain 1* | 1.22E-02 | 1.51 |
| NM_005957 | *MTHFR* | | | | *methylenetetrahydrofolate reductase (NAD(P)H)* | 2.27E-02 | 1.51 |
| NM_133467 | *CITED4* | | | | *Cbp/p300-interacting transactivator, with Glu/Asp-rich carboxy-terminal domain, 4* | 1.79E-03 | 1.51 |
| NM_001174087 /// NM_001174088 /// NM_006534 /// NM_181659 | *NCOA3* | | | | *nuclear receptor coactivator 3* | 2.99E-03 | 1.51 |
| NM_021203 | *SRPRB* | | | | *signal recognition particle receptor, B subunit* | 4.15E-02 | 1.51 |
| NM_001171506 /// NM_001171507 /// NM_001171508 /// NM_001171509 /// NM_001171510 | *MCFD2* | | | | *multiple coagulation factor deficiency 2* | 1.47E-02 | 1.51 |
| NM_020533 | *MCOLN1* | | | | *mucolipin 1* | 4.65E-02 | 1.51 |
| NM_001011708 /// NM_013341 | *OLA1* | | | | *Obg-like ATPase 1* | 2.80E-02 | 1.51 |
| NM_001166050 /// NM_001166051 /// NM_001166052 /// NM_001166053 | *APBB2* | | | | *amyloid beta (A4) precursor protein-binding, family B, member 2* | 3.13E-02 | 1.51 |
| NM_000169 | *GLA* | | | | *galactosidase, alpha* | 3.31E-02 | 1.51 |
| NM_001018111 /// NM_005397 | *PODXL* | | | | *podocalyxin-like* | 2.12E-02 | 1.51 |
| NM_001166347 /// NM_001166348 /// NM_001166349 /// NM_173626 | *SLC26A11* | | | | *solute carrier family 26, member 11* | 2.55E-02 | 1.50 |
| NM_017732 /// NM_177938 /// NM_177939 | *P4HTM* | | | | *prolyl 4-hydroxylase, transmembrane (endoplasmic reticulum)* | 2.36E-02 | 1.50 |
| NM_000188 /// NM_033496 /// NM_033497 /// NM_033498 | *HK1* | | | | *hexokinase 1* | 3.73E-02 | 1.50 |
| NM_152261 | *C12orf23* | | | | *chromosome 12 open reading frame 23* | 1.72E-02 | 1.50 |
| NM_015954 | *DERA* | | | | *deoxyribose-phosphate aldolase (putative)* | 1.86E-03 | -1.50 |
| NM_001122824 /// NM_001146216 /// NM_006283 | *TACC1* | | | | *transforming, acidic coiled-coil containing protein 1* | 6.47E-03 | -1.50 |
| NM_001134470 /// NM_173552 | *C3orf58* | | | | *chromosome 3 open reading frame 58* | 3.15E-02 | -1.50 |
| NM_001129993 /// NM_032506 | *KIAA1841* | | | | *KIAA1841* | 3.53E-03 | -1.50 |
| NM_001272011 /// NM_014226 /// NR_073540 /// NR_073541 /// NR_073542 /// NR_073543 | *MOK* | | | | *MOK protein kinase* | 7.78E-03 | -1.50 |
| NM_001003795 /// NM_173537 | *GTF2IRD2 /// GTF2IRD2B* | | | | *GTF2I repeat domain containing 2 /// GTF2I repeat domain containing 2B* | 2.11E-02 | -1.51 |
| NM_001144924 /// NM_001199053 /// NM_030941 | *LOC81691* | | | | *exonuclease NEF-sp* | 4.12E-02 | -1.51 |
| NM_016058 | *TPRKB* | | | | *TP53RK binding protein* | 3.92E-02 | -1.51 |
| NM_145063 | *OARD1* | | | | *O-acyl-ADP-ribose deacylase 1* | 1.74E-02 | -1.51 |
| NM_001134875 /// NM_001134876 /// NM_001134877 /// NM_001198983 /// NM_173608 | *C14orf80* | | | | *chromosome 14 open reading frame 80* | 3.47E-02 | -1.51 |
| NM_001134647 /// NM_021638 /// NM_198595 | *AFAP1* | | | | *actin filament associated protein 1* | 2.06E-03 | -1.51 |
| NM_001256267 /// NM_001256268 /// NM_032578 /// NR_045662 /// NR_045663 | *MYPN* | | | | *myopalladin* | 1.58E-02 | -1.51 |
| NM_018159 | *NUDT11* | | | | *nudix (nucleoside diphosphate linked moiety X)-type motif 11* | 2.44E-02 | -1.51 |
| NM_033121 | *ANKRD13A* | | | | *ankyrin repeat domain 13A* | 4.65E-03 | -1.51 |
| NM_003405 | *YWHAH* | | | | *tyrosine 3-monooxygenase/tryptophan 5-monooxygenase activation protein, eta polypeptide* | 4.28E-02 | -1.51 |
| NM_001256658 /// NM_001256659 /// NM_001256660 /// NM_001256661 /// NM_001256662 | *TEAD2* | | | | *TEA domain family member 2* | 9.64E-03 | -1.51 |
| NM_015225 /// NM_138818 | *PRUNE2* | | | | *prune homolog 2 (Drosophila)* | 2.97E-02 | -1.51 |
| NM_001201480 /// NM_001201481 /// NM_001201482 /// NM_032523 /// NM_145739 | *OSBPL6* | | | | *oxysterol binding protein-like 6* | 3.67E-03 | -1.52 |
| NM_145307 | *RTKN2* | | | | *rhotekin 2* | 3.95E-02 | -1.52 |
| NM_001199295 /// NM_153263 | *ZNF549* | | | | *zinc finger protein 549* | 3.33E-02 | -1.52 |
| NM_001124 | *ADM* | | | | *adrenomedullin* | 6.65E-03 | -1.52 |
| NM_000249 /// NM_001167617 /// NM_001167618 /// NM_001167619 /// NM_001258271 | *MLH1* | | | | *mutL homolog 1, colon cancer, nonpolyposis type 2 (E. coli)* | 4.60E-02 | -1.52 |
| NM_001001701 /// NM_001170330 | *C4orf3* | | | | *chromosome 4 open reading frame 3* | 8.51E-03 | -1.52 |
| NM_001130834 /// NM_001290 | *LDB2* | | | | *LIM domain binding 2* | 3.00E-02 | -1.52 |
| NM_001258390 /// NM_001258391 /// NM_178834 | *LAYN* | | | | *layilin* | 2.75E-02 | -1.52 |
| NM_001271667 /// NM_001271668 /// NM_001271669 /// NM_032122 /// NM_183040 | *DTNBP1* | | | | *dystrobrevin binding protein 1* | 3.62E-02 | -1.52 |
| NM_001008938 /// NM_014756 | *CKAP5* | | | | *cytoskeleton associated protein 5* | 6.38E-03 | -1.52 |
| NM_000373 /// NR_033434 /// NR_033437 | *UMPS* | | | | *uridine monophosphate synthetase* | 3.22E-02 | -1.52 |
| NM_199342 | *CCDC23* | | | | *coiled-coil domain containing 23* | 2.00E-02 | -1.52 |
| NM_001115007 /// NM_001115008 /// NM_194282 | *LIN54* | | | | *lin-54 homolog (C. elegans)* | 1.65E-03 | -1.53 |
| NM_000022 | *ADA* | | | | *adenosine deaminase* | 3.62E-02 | -1.53 |
| NM_001224 /// NM_032982 /// NM_032983 | *CASP2* | | | | *caspase 2, apoptosis-related cysteine peptidase* | 4.20E-02 | -1.53 |
| NM_032336 | *GINS4* | | | | *GINS complex subunit 4 (Sld5 homolog)* | 1.33E-02 | -1.53 |
| NM_001130914 /// NM_006806 | *BTG3* | | | | *BTG family, member 3* | 4.71E-02 | -1.53 |
| NM_016441 | *CRIM1* | | | | *cysteine rich transmembrane BMP regulator 1 (chordin-like)* | 3.46E-02 | -1.53 |
| NM_194313 | *KIF24* | | | | *kinesin family member 24* | 2.52E-02 | -1.53 |
| NM_194272 | *RBPMS2* | | | | *RNA binding protein with multiple splicing 2* | 7.26E-03 | -1.53 |
| NM_001242795 /// NM_001242796 /// NM_014669 | *NUP93* | | | | *nucleoporin 93kDa* | 1.64E-02 | -1.54 |
| NM_001195573 /// NM_001271282 /// NM_030621 /// NM_177438 | *DICER1* | | | | *dicer 1, ribonuclease type III* | 1.90E-03 | -1.54 |
| NM_001243597 /// NM_016952 | *CDON* | | | | *cell adhesion associated, oncogene regulated* | 1.63E-02 | -1.54 |
| NM_006275 /// NR_034009 | *SRSF6* | | | | *serine/arginine-rich splicing factor 6* | 2.68E-02 | -1.54 |
| NM_003480 | *MFAP5* | | | | *microfibrillar associated protein 5* | 3.91E-02 | -1.54 |
| NM_021947 | *SRR* | | | | *serine racemase* | 1.86E-02 | -1.54 |
| NM_001172420 /// NM_018100 /// NR_033327 | *EFHC1* | | | | *EF-hand domain (C-terminal) containing 1* | 6.09E-03 | -1.54 |
| NM_004499 /// NM_031266 | *HNRNPAB* | | | | *heterogeneous nuclear ribonucleoprotein A/B* | 1.65E-02 | -1.54 |
| NM_015207 | *OTUD3* | | | | *OTU domain containing 3* | 8.21E-03 | -1.54 |
| NM_138702 /// NM_177456 | *MAGEC3* | | | | *melanoma antigen family C, 3* | 1.14E-02 | -1.54 |
| NM_001267556 /// NM_001267557 /// NM_001267558 /// NM_001267559 /// NM_013232 | *PDCD6* | | | | *programmed cell death 6* | 2.47E-02 | -1.54 |
| NM_001031714 /// NM_022489 /// NM_032714 | *INF2* | | | | *inverted formin, FH2 and WH2 domain containing* | 1.81E-02 | -1.55 |
| NM_015549 | *PLEKHG3* | | | | *pleckstrin homology domain containing, family G (with RhoGef domain) member 3* | 7.43E-03 | -1.55 |
| NM_001238 /// NM_057182 | *CCNE1* | | | | *cyclin E1* | 3.81E-02 | -1.55 |
| NM_174902 | *LDLRAD3* | | | | *low density lipoprotein receptor class A domain containing 3* | 2.80E-02 | -1.55 |
| NR_002436 | *SNORA33* | | | | *small nucleolar RNA, H/ACA box 33* | 1.51E-03 | -1.55 |
| NM_001243776 /// NM_001243777 /// NM_014679 | *CEP57* | | | | *centrosomal protein 57kDa* | 3.26E-03 | -1.55 |
| NM_013242 | *C16orf80* | | | | *chromosome 16 open reading frame 80* | 1.90E-02 | -1.55 |
| NM_001164603 /// NM_015338 | *ASXL1* | | | | *additional sex combs like 1 (Drosophila)* | 2.50E-02 | -1.55 |
| NM_001171967 /// NM_024805 | *RBFA* | | | | *ribosome binding factor A (putative)* | 4.49E-03 | -1.55 |
| NM_001010844 | *IRAK1BP1* | | | *interleukin-1 receptor-associated kinase 1 binding protein 1* | | 2.61E-02 | -1.56 |
| NM_004436 /// NM_207042 /// NM_207043 /// NM_207044 /// NM_207045 /// NM_207046 | *ENSA* | | | | *endosulfine alpha* | 3.01E-03 | -1.56 |
| NM_015076 | *CDK19* | | | | *cyclin-dependent kinase 19* | 4.29E-02 | -1.56 |
| NM_006092 | *NOD1* | | | | *nucleotide-binding oligomerization domain containing 1* | 3.73E-02 | -1.56 |
| NM_147189 | *FAM110B* | | | | *family with sequence similarity 110, member B* | 4.45E-03 | -1.56 |
| NM_001191005 /// NM_001191006 /// NM_001191007 /// NM_001191009 /// NM_006625 | *SRSF10* | | | | *serine/arginine-rich splicing factor 10* | 8.44E-04 | -1.56 |
| NM_006031 | *PCNT* | | | | *pericentrin* | 3.97E-02 | -1.56 |
| NM_017518 /// NM_207106 /// NM_207107 /// NR_073156 | *HAUS7* | | | | *HAUS augmin-like complex, subunit 7* | 2.24E-02 | -1.56 |
| NM_001130721 /// NM_024090 | *ELOVL6* | | | | *ELOVL fatty acid elongase 6* | 3.20E-02 | -1.56 |
| NM_000135 /// NM_001018112 | *FANCA* | | | | *Fanconi anemia, complementation group A* | 4.26E-02 | -1.57 |
| NM_020190 | *OLFML3* | | | | *olfactomedin-like 3* | 3.97E-02 | -1.57 |
| NM_032876 /// NM_198086 | *AJUBA* | | | | *ajuba LIM protein* | 2.78E-02 | -1.57 |
| NM_001033505 /// NM_001033506 /// NM_001326 | *CSTF3* | | | | *cleavage stimulation factor, 3' pre-RNA, subunit 3, 77kDa* | 2.97E-02 | -1.57 |
| NM_001040458 /// NM_001198541 /// NM_016442 | *ERAP1* | | | | *endoplasmic reticulum aminopeptidase 1* | 2.67E-02 | -1.57 |
| NM_001166269 /// NM_001166270 /// NM_017815 /// NR_039856 | *HAUS4 /// MIR4707* | | | | *HAUS augmin-like complex, subunit 4 /// microRNA 4707* | 3.76E-02 | -1.57 |
| NM_001195098 /// NM_001195099 /// NM_015199 | *ANKRD28* | | | | *ankyrin repeat domain 28* | 2.25E-02 | -1.58 |
| NM_001256574 /// NM_001256575 /// NM_001256576 /// NM_003633 /// NR_046318 | *ENC1* | | | | *ectodermal-neural cortex 1 (with BTB domain)* | 2.60E-02 | -1.58 |
| NM_001080449 /// NR_102264 | *DNA2* | | | | *DNA replication helicase/nuclease 2* | 1.98E-02 | -1.58 |
| NM_001099776 /// NM_018392 /// NM_138698 | *C4orf21* | | | | *chromosome 4 open reading frame 21* | 2.69E-02 | -1.58 |
| NM_001402 /// NM_001403 | *EEF1A1* | | | | *eukaryotic translation elongation factor 1 alpha 1* | 9.68E-03 | -1.58 |
| NM_001042724 /// NM_002856 | *PVRL2* | | | *poliovirus receptor-related 2 (herpesvirus entry mediator B)* | | 1.71E-02 | -1.59 |
| NM_016270 | *KLF2* | | | | *Kruppel-like factor 2 (lung)* | 4.39E-02 | -1.59 |
| NM_001193357 /// NM_012346 /// NM_016553 /// NM_153718 /// NM_153719 | *NUP62* | | | | *nucleoporin 62kDa* | 2.16E-02 | -1.59 |
| NM_014426 /// NM_152227 | *SNX5* | | | | *sorting nexin 5* | 2.95E-02 | -1.59 |
| NM_002945 | *RPA1* | | | | *replication protein A1, 70kDa* | 3.43E-02 | -1.59 |
| NM_001244584 /// NM_197964 /// NR_073058 /// NR_073059 /// NR_073060 | *C7orf55* | | | | *chromosome 7 open reading frame 55 /// C7orf55-LUC7L2 readthrough* | 4.48E-02 | -1.59 |
| NM_022774 | *EXO5* | | | | *exonuclease 5* | 1.38E-02 | -1.59 |
| NM_152341 | *PAQR4* | | | | *progestin and adipoQ receptor family member IV* | 1.16E-02 | -1.60 |
| NM_016049 | *EMC9* | | | | *ER membrane protein complex subunit 9* | 4.32E-02 | -1.60 |
| NM_012105 /// NM_138991 /// NM_138992 | *BACE2* | | | | *beta-site APP-cleaving enzyme 2* | 4.45E-02 | -1.60 |
| NM_001001715 /// NM_005766 | *FARP1* | | | | *FERM, RhoGEF (ARHGEF) and pleckstrin domain protein 1 (chondrocyte-derived)* | 1.25E-02 | -1.60 |
| NM_001040152 /// NM_001172437 /// NM_001172438 /// NM_001184961 /// NM_001184962 | | *PEG10* | | | *paternally expressed 10* | 4.42E-02 | -1.60 |
| NM_004580 /// NM_183234 /// NM_183235 /// NM_183236 | *RAB27A* | | | | *RAB27A, member RAS oncogene family* | 4.29E-02 | -1.60 |
| NM_052862 | *RCSD1* | | | | *RCSD domain containing 1* | 7.51E-03 | -1.61 |
| NM_006026 | *H1FX* | | | | *H1 histone family, member X* | 3.78E-02 | -1.61 |
| NM_001042618 /// NM_005484 | *PARP2* | | | | *poly (ADP-ribose) polymerase 2* | 7.22E-03 | -1.61 |
| NM_001256371 /// NM_001256372 /// NM_024680 | *E2F8* | | | | *E2F transcription factor 8* | 3.60E-02 | -1.62 |
| NM_006265 | *RAD21* | | | | *RAD21 homolog (S. pombe)* | 4.11E-02 | -1.62 |
| NM_007172 /// NM_153645 | *NUP50* | | | | *nucleoporin 50kDa* | 7.52E-03 | -1.62 |
| NM_004537 /// NM_139207 | *NAP1L1* | | | | *nucleosome assembly protein 1-like 1* | 4.93E-03 | -1.62 |
| NM_001001323 /// NM_001682 | *ATP2B1* | | | | *ATPase, Ca++ transporting, plasma membrane 1* | 3.86E-02 | -1.63 |
| NM_001039348 /// NM_001039349 /// NM_004105 | *EFEMP1* | | | | *EGF containing fibulin-like extracellular matrix protein 1* | 2.29E-02 | -1.63 |
| NM_001199290 /// NM_144711 | *KLHL23 /// PHOSPHO2-KLHL23* | | | | *kelch-like family member 23 /// PHOSPHO2-KLHL23 readthrough* | 4.29E-02 | -1.63 |
| NM_001258217 /// NM_001258218 /// NM_001258219 /// NM_001258220 /// NM_024039 | *MIS12* | | | | *MIS12, MIND kinetochore complex component, homolog (S. pombe)* | 1.97E-02 | -1.64 |
| NM_007018 | *CNTRL* | | | | *centriolin* | 1.73E-02 | -1.64 |
| NM_138569 | *MLIP* | | | | *muscular LMNA-interacting protein* | 3.62E-02 | -1.64 |
| NM_012176 /// NM_033484 | *FBXO4* | | | | *F-box protein 4* | 9.02E-04 | -1.64 |
| NM_015101 | *COLGALT2* | | | | *collagen beta(1-O)galactosyltransferase 2* | 4.44E-02 | -1.64 |
| NM_001195685 /// NM_194317 | *LYPD6* | | | | *LY6/PLAUR domain containing 6* | 1.24E-02 | -1.64 |
| NM_001007793 /// NM_004725 | *BUB3* | | | | *BUB3 mitotic checkpoint protein* | 3.29E-02 | -1.64 |
| NM_001252197 /// NM_005853 | *IRX5* | | | | *iroquois homeobox 5* | 8.49E-03 | -1.64 |
| NM_206886 | *CCDC18* | | | | *coiled-coil domain containing 18* | 4.49E-02 | -1.64 |
| NM_001242359 /// NM_014836 /// NR_024554 /// NR_024555 | *RHOBTB1* | | | | *Rho-related BTB domain containing 1* | 3.41E-02 | -1.64 |
| NM_138443 /// NR_026978 | *HAUS1* | | | | *HAUS augmin-like complex, subunit 1* | 4.07E-02 | -1.65 |
| NM_006231 | *POLE* | | | | *polymerase (DNA directed), epsilon, catalytic subunit* | 3.28E-02 | -1.65 |
| NM_002998 | *SDC2* | | | | *syndecan 2* | 2.32E-02 | -1.65 |
| NM_145702 | *TIGD1* | | | | *tigger transposable element derived 1* | 5.69E-03 | -1.65 |
| NM_199249 /// NM_199250 /// NR_003069 | *C19orf48 /// SNORD88C* | | | | *chromosome 19 open reading frame 48 /// small nucleolar RNA, C/D box 88C* | 1.65E-02 | -1.65 |
| NM_000991 /// NM_001136134 /// NM_001136135 /// NM_001136136 /// NM_001136137 | *RPL28* | | | | *ribosomal protein L28* | 2.92E-02 | -1.65 |
| NM_018361 | *AGPAT5* | | | | *1-acylglycerol-3-phosphate O-acyltransferase 5* | 3.70E-02 | -1.65 |
| NM_006951 | *TAF5* | | | | *TAF5 RNA polymerase II, TATA box binding protein (TBP)-associated factor, 100kDa* | 2.02E-02 | -1.66 |
| NM_001424 | *EMP2* | | | | *epithelial membrane protein 2* | 8.12E-04 | -1.66 |
| NM_003108 | *SOX11* | | | | *SRY (sex determining region Y)-box 11* | 4.05E-02 | -1.66 |
| NM_018288 /// NM_133325 | *PHF10* | | | | *PHD finger protein 10* | 4.60E-02 | -1.66 |
| NM_025108 | *C16orf59* | | | | *chromosome 16 open reading frame 59* | 1.79E-02 | -1.66 |
| NM_006401 | *ANP32B* | | | | *acidic (leucine-rich) nuclear phosphoprotein 32 family, member B* | 2.61E-02 | -1.66 |
| NM_005030 | *PLK1* | | | | *polo-like kinase 1* | 3.66E-02 | -1.66 |
| NM_001002032 /// NM_001002033 /// NM_016185 | *HN1* | | | | *hematological and neurological expressed 1* | 1.17E-03 | -1.66 |
| NM_001199022 /// NM_014711 | *CCP110* | | | | *centriolar coiled coil protein 110kDa* | 3.86E-02 | -1.67 |
| NM_144570 | *HN1L* | | | | *hematological and neurological expressed 1-like* | 2.24E-02 | -1.67 |
| NM_001145713 /// NM_001145714 /// NM_005500 /// NR_027280 | | *SAE1* | | | *SUMO1 activating enzyme subunit 1* | 2.92E-02 | -1.67 |
| NM_032827 | *ATOH8* | | | | *atonal homolog 8 (Drosophila)* | 2.14E-02 | -1.67 |
| NM_024096 | *DCTPP1* | | | | *dCTP pyrophosphatase 1* | 3.43E-02 | -1.68 |
| NM_001270472 /// NM_002388 | *MCM3* | | | | *minichromosome maintenance complex component 3* | 4.92E-02 | -1.68 |
| NM_006681 | *NMU* | | | | *neuromedin U* | 3.53E-02 | -1.68 |
| NM_002883 | *RANGAP1* | | | | *Ran GTPase activating protein 1* | 3.06E-03 | -1.68 |
| NM_001126336 /// NM_001164097 /// NM_001164098 /// NM_004385 | | *VCAN* | | | *versican* | 1.81E-02 | -1.69 |
| NM_032246 | *MEX3B* | | | | *mex-3 homolog B (C. elegans)* | 2.71E-02 | -1.69 |
| NM_001007563 | *IGFBPL1* | | | | *insulin-like growth factor binding protein-like 1* | 8.85E-03 | -1.69 |
| NM_006166 | *NFYB* | | | | *nuclear transcription factor Y, beta* | 1.91E-02 | -1.69 |
| NM_001164391 /// NM_001164392 /// NM_001164393 /// NM_007358 | *MTF2* | | | | *metal response element binding transcription factor 2* | 7.58E-03 | -1.69 |
| NM_001184705 /// NM_005327 | *HADH* | | | | *hydroxyacyl-CoA dehydrogenase* | 3.08E-02 | -1.69 |
| NM_001127325 /// NM_006341 | *MAD2L2* | | | | *MAD2 mitotic arrest deficient-like 2 (yeast)* | 2.30E-02 | -1.70 |
| NM_001143905 /// NM_001194995 /// NM_152269 | *C12orf65* | | | | *chromosome 12 open reading frame 65* | 4.20E-02 | -1.71 |
| NM_002947 | *RPA3* | | | | *replication protein A3, 14kDa* | 4.85E-02 | -1.71 |
| NM_012291 | *ESPL1* | | | | *extra spindle pole bodies homolog 1 (S. cerevisiae)* | 4.17E-02 | -1.71 |
| NM_001002857 /// NM_001002858 /// NM_001136015 /// NM_004039 | *ANXA2* | | | | *annexin A2* | 4.96E-02 | -1.71 |
| NM_000060 | *BTD* | | | | *biotinidase* | 2.91E-02 | -1.71 |
| NM_004301 /// NM_177989 /// NM_178042 | *ACTL6A* | | | | *actin-like 6A* | 2.24E-02 | -1.72 |
| NM_003173 | *SUV39H1* | | | | *suppressor of variegation 3-9 homolog 1 (Drosophila)* | 3.92E-03 | -1.72 |
| NM_001197293 /// NM_001244604 /// NM_001386 | *DPYSL2* | | | | *dihydropyrimidinase-like 2* | 5.28E-04 | -1.72 |
| NM_000817 /// NM_013445 | *GAD1* | | | | *glutamate decarboxylase 1 (brain, 67kDa)* | 3.66E-02 | -1.72 |
| NM_001195602 /// NM_001195604 /// NM_012247 | *SEPHS1* | | | | *selenophosphate synthetase 1* | 2.41E-02 | -1.72 |
| NM_020992 | *PDLIM1* | | | | *PDZ and LIM domain 1* | 2.73E-02 | -1.73 |
| NM_024546 | *RNF219* | | | | *ring finger protein 219* | 6.87E-03 | -1.73 |
| NM_032354 /// NM_183065 | *TMEM107* | | | | *transmembrane protein 107* | 2.24E-02 | -1.73 |
| NM_005482 | *PIGK* | | | | *phosphatidylinositol glycan anchor biosynthesis, class K* | 4.39E-02 | -1.73 |
| NM_001085399 /// NM_001085400 | *RELL1* | | | | *RELT-like 1* | 1.20E-02 | -1.73 |
| NM_001136469 /// NM_023943 | *TMEM108* | | | | *transmembrane protein 108* | 1.58E-02 | -1.73 |
| NM_001127228 /// NM_006807 | *CBX1* | | | | *chromobox homolog 1* | 2.38E-02 | -1.74 |
| NM_022092 | *CHTF18* | | | | *CTF18, chromosome transmission fidelity factor 18 homolog (S. cerevisiae)* | 3.60E-02 | -1.74 |
| NM_001030060 | *SAMD5* | | | | *sterile alpha motif domain containing 5* | 1.40E-02 | -1.74 |
| NM_015419 | *MXRA5* | | | | *matrix-remodelling associated 5* | 9.81E-04 | -1.74 |
| NM_001010866 /// NM_001130924 | *TMEM201* | | | | *transmembrane protein 201* | 3.44E-02 | -1.74 |
| NM_004755 /// NM_182398 | *RPS6KA5* | | | | *ribosomal protein S6 kinase, 90kDa, polypeptide 5* | 1.17E-02 | -1.75 |
| NM_001164326 /// NM_006696 /// NM_139199 /// NM_183359 | *BRD8* | | | | *bromodomain containing 8* | 7.76E-03 | -1.75 |
| NM_001130009 /// NM_182625 | *GEN1* | | | | *GEN1 Holliday junction 5' flap endonuclease* | 7.97E-03 | -1.75 |
| NM_014722 /// NM_015864 | *FAM65B* | | | | *family with sequence similarity 65, member B* | 3.29E-02 | -1.76 |
| NM_012412 /// NM_138635 /// NM_201436 /// NM_201516 /// NM_201517 | *H2AFV* | | | | *H2A histone family, member V* | 2.98E-02 | -1.76 |
| NM_020401 | *NUP107* | | | | *nucleoporin 107kDa* | 1.52E-02 | -1.76 |
| NM_001008274 | *TRIM72* | | | | *tripartite motif containing 72* | 2.69E-02 | -1.76 |
| NM_152637 | *METTL7B* | | | | *methyltransferase like 7B* | 3.89E-02 | -1.76 |
| NM_001424 | *EMP2* | | | | *epithelial membrane protein 2* | 1.86E-02 | -1.77 |
| NM_001031684 /// NM_001195446 /// NM_006276 | *SRSF7* | | | | *serine/arginine-rich splicing factor 7* | 2.59E-02 | -1.77 |
| NM_000079 /// NM_001039523 | *CHRNA1* | | | | *cholinergic receptor, nicotinic, alpha 1 (muscle)* | 1.00E-02 | -1.77 |
| NM_006162 /// NM_172387 /// NM_172388 /// NM_172389 /// NM_172390 | *NFATC1* | | | | *nuclear factor of activated T-cells, cytoplasmic, calcineurin-dependent 1* | 1.65E-02 | -1.77 |
| NM_018843 | *SLC25A40* | | | | *solute carrier family 25, member 40* | 1.36E-02 | -1.77 |
| NM_021922 | *FANCE* | | | | *Fanconi anemia, complementation group E* | 5.58E-03 | -1.77 |
| NM_015147 | *CEP68* | | | | *centrosomal protein 68kDa* | 5.22E-03 | -1.77 |
| NM_001144964 /// NM_001144965 /// NM_001144966 /// NM_001144967 /// NM_001144968 | *NEDD4L* | | | | *neural precursor cell expressed, developmentally down-regulated 4-like, E3 ubiquitin protein ligase* | 3.32E-02 | -1.78 |
| NM_001040409 /// NM_006636 /// NR_027405 | *MTHFD2* | | | | *methylenetetrahydrofolate dehydrogenase (NADP+ dependent) 2, methenyltetrahydrofolate c* | 3.33E-02 | -1.78 |
| NM_017588 /// NM_052821 | *WDR5* | | | | *WD repeat domain 5* | 6.49E-04 | -1.78 |
| NM_016396 | *CTDSPL2* | | | | *CTD (carboxy-terminal domain, RNA polymerase II, polypeptide A) small phosphatase like* | 3.59E-02 | -1.78 |
| NM_022906 /// NR_040585 /// NR_040586 | *STAG3L4* | | | | *stromal antigen 3-like 4* | 3.49E-02 | -1.78 |
| NM_001204402 /// NM_002764 | *PRPS1* | | | | *phosphoribosyl pyrophosphate synthetase 1* | 2.21E-02 | -1.78 |
| NM_001159699 /// NM_001159700 /// NM_001159701 /// NM_001159702 /// NM_001159703 | | *FHL1* | | | *four and a half LIM domains 1* | 1.42E-02 | -1.78 |
| NM_001010927 | *TIAM2* | | | | *T-cell lymphoma invasion and metastasis 2* | 2.58E-02 | -1.78 |
| NM_000747 | *CHRNB1* | | | | *cholinergic receptor, nicotinic, beta 1 (muscle)* | 2.98E-02 | -1.78 |
| NM_001039841 | *ARHGAP11B* | | | | *Rho GTPase activating protein 11B* | 3.17E-02 | -1.79 |
| NM_006527 | *SLBP* | | | | *stem-loop binding protein* | 4.73E-02 | -1.80 |
| NM_001031720 /// NM_024751 | *GSTCD* | | | | *glutathione S-transferase, C-terminal domain containing* | 1.68E-03 | -1.80 |
| NM_001143941 /// NM_001143942 /// NM_153020 | *RBM24* | | | | *RNA binding motif protein 24* | 2.36E-03 | -1.80 |
| NM_001159736 /// NM_032725 | *BUD13* | | | | *BUD13 homolog (S. cerevisiae)* | 4.84E-02 | -1.80 |
| NM_014059 | *RGCC* | | | | *regulator of cell cycle* | 4.85E-04 | -1.80 |
| NM_005796 | *NUTF2* | | | | *nuclear transport factor 2* | 3.06E-02 | -1.80 |
| NM_005708 | *GPC6* | | | | *glypican 6* | 3.68E-02 | -1.80 |
| NM_004462 | *FDFT1* | | | | *farnesyl-diphosphate farnesyltransferase 1* | 3.25E-03 | -1.80 |
| NM_001008938 /// NM_014756 | *CKAP5* | | | | *cytoskeleton associated protein 5* | 5.52E-03 | -1.81 |
| NM_018283 | *NUDT15* | | *nudix (nucleoside diphosphate linked moiety X)-type motif 15* | | | 4.38E-02 | -1.81 |
| NM_001040633 /// NM_016203 /// NM_024429 | *PRKAG2* | | | | *protein kinase, AMP-activated, gamma 2 non-catalytic subunit* | 1.06E-02 | -1.81 |
| NM_017858 | *TIPIN* | | | | *TIMELESS interacting protein* | 2.55E-02 | -1.81 |
| NM_001142653 /// NM_001142654 /// NM_001261430 | *PTGES3L* | | | | *prostaglandin E synthase 3 (cytosolic)-like* | 2.05E-02 | -1.81 |
| NM_024844 | *NUP85* | | | | *nucleoporin 85kDa* | 4.52E-02 | -1.81 |
| NM_031283 | *TCF7L1* | | | | *transcription factor 7-like 1 (T-cell specific, HMG-box)* | 1.73E-02 | -1.81 |
| NM_014285 | *EXOSC2* | | | | *exosome component 2* | 3.29E-02 | -1.82 |
| NM_004342 /// NM_033138 /// NM_033139 /// NM_033140 /// NM_033157 | *CALD1* | | | | *caldesmon 1* | 3.96E-02 | -1.82 |
| NM_001114636 /// NM_018062 | *FANCL* | | | | *Fanconi anemia, complementation group L* | 3.61E-02 | -1.82 |
| NM_001206957 /// NM_007182 /// NM_170712 /// NM_170713 /// NM_170714 /// NM_170715 | *RASSF1* | | | | *Ras association (RalGDS/AF-6) domain family member 1* | 3.88E-02 | -1.83 |
| NM_004414 /// NM_203417 /// NM_203418 | *RCAN1* | | | | *regulator of calcineurin 1* | 4.42E-02 | -1.83 |
| NM_001202 /// NM_130850 /// NM_130851 | *BMP4* | | | | *bone morphogenetic protein 4* | 5.61E-03 | -1.83 |
| NM_001010853 | *PM20D2* | | | | *peptidase M20 domain containing 2* | 6.69E-04 | -1.83 |
| NM_001111077 /// NM_003379 | *EZR* | | | | *ezrin* | 1.52E-03 | -1.83 |
| NM_001172309 /// NM_144573 | *NEXN* | | | | *nexilin (F actin binding protein)* | 4.53E-02 | -1.83 |
| NM_024619 /// NR_046408 | *FN3KRP* | | | | *fructosamine 3 kinase related protein* | 2.04E-03 | -1.83 |
| NM_000885 | *ITGA4* | | | | *integrin, alpha 4 (antigen CD49D, alpha 4 subunit of VLA-4 receptor)* | 1.75E-02 | -1.83 |
| NM_031453 | *FAM107B* | | | | *family with sequence similarity 107, member B* | 4.71E-02 | -1.83 |
| NM_015675 | *GADD45B* | | | | *growth arrest and DNA-damage-inducible, beta* | 4.11E-02 | -1.84 |
| NM_003507 | *FZD7* | | | | *frizzled family receptor 7* | 1.79E-02 | -1.84 |
| NM_001071775 | *MZT1* | | | | *mitotic spindle organizing protein 1* | 2.75E-02 | -1.84 |
| NM_000994 /// NM_001007073 /// NM_001007074 | *RPL32* | | | | *ribosomal protein L32* | 2.21E-02 | -1.84 |
| NM_032818 | *ARHGEF39* | | | | *Rho guanine nucleotide exchange factor (GEF) 39* | 4.80E-02 | -1.85 |
| NM_000689 | *ALDH1A1* | | | | *aldehyde dehydrogenase 1 family, member A1* | 8.38E-03 | -1.85 |
| NM_021178 /// NM_182849 /// NM_182851 /// NM_182852 | *CCNB1IP1* | | | | *cyclin B1 interacting protein 1, E3 ubiquitin protein ligase* | 8.06E-03 | -1.85 |
| NM_001127321 /// NM_001127322 /// NM_012117 | *CBX5* | | | | *chromobox homolog 5* | 2.03E-02 | -1.86 |
| NM_175856 | *CHSY3* | | | | *chondroitin sulfate synthase 3* | 4.45E-02 | -1.86 |
| NM_017696 /// NM_153255 | *MCM9* | | | | *minichromosome maintenance complex component 9* | 3.13E-02 | -1.86 |
| NM_002946 | *RPA2* | | | | *replication protein A2, 32kDa* | 4.26E-02 | -1.87 |
| NM_001135175 /// NM_001135176 /// NM_001135177 /// NM_152905 | *NEDD1* | | | | *neural precursor cell expressed, developmentally down-regulated 1* | 7.14E-03 | -1.87 |
| NM_001008744 /// NM_018319 | *TDP1* | | | | *tyrosyl-DNA phosphodiesterase 1* | 8.78E-04 | -1.87 |
| NM_001093725 | *MEX3A* | | | | *mex-3 homolog A (C. elegans)* | 2.08E-03 | -1.88 |
| NM_003885 | *CDK5R1* | | | | *cyclin-dependent kinase 5, regulatory subunit 1 (p35)* | 4.66E-02 | -1.88 |
| NM_002894 /// NM_203291 /// NM_203292 | *RBBP8* | | | | *retinoblastoma binding protein 8* | 4.26E-02 | -1.88 |
| NM_001130690 /// NM_006661 /// NR_045597 | *PDE10A* | | | | *phosphodiesterase 10A* | 3.24E-02 | -1.89 |
| NM_001100619 /// NM_001256438 /// NM_138375 /// NR_023359 | *CABLES1* | | | | *Cdk5 and Abl enzyme substrate 1* | 4.13E-03 | -1.89 |
| NM_020123 | *TM9SF3* | | | | *transmembrane 9 superfamily member 3* | 4.83E-02 | -1.89 |
| NM_001033673 /// NM_002853 /// NM_133282 /// NM_133377 /// NR_026591 | *RAD1* | | | | *RAD1 homolog (S. pombe)* | 3.93E-02 | -1.90 |
| NM_030928 | *CDT1* | | | | *chromatin licensing and DNA replication factor 1* | 2.71E-02 | -1.90 |
| NM_005590 /// NM_005591 | *MRE11A* | | | | *MRE11 meiotic recombination 11 homolog A (S. cerevisiae)* | 4.57E-02 | -1.91 |
| NM_001616 | *ACVR2A* | | | | *activin A receptor, type IIA* | 1.05E-02 | -1.91 |
| NM_001256171 /// NM_001256172 /// NM_001256173 /// NM_003429 /// NR_045830 | *ZNF85* | | | | *zinc finger protein 85* | 3.86E-02 | -1.91 |
| NM_000459 | *TEK* | | | | *TEK tyrosine kinase, endothelial* | 1.19E-02 | -1.92 |
| NM_004629 | *FANCG* | | | | *Fanconi anemia, complementation group G* | 4.95E-02 | -1.92 |
| NM_001134848 | *CCDC152* | | | | *coiled-coil domain containing 152* | 4.53E-02 | -1.93 |
| NM_018663 | *PXMP2* | | | | *peroxisomal membrane protein 2, 22kDa* | 6.88E-03 | -1.94 |
| NM_006795 | *EHD1* | | | | *EH-domain containing 1* | 3.75E-02 | -1.94 |
| NM_015261 | *NCAPD3* | | | | *non-SMC condensin II complex, subunit D3* | 3.35E-02 | -1.94 |
| NR_034105 /// NR_034106 | *CRNDE* | | | | *colorectal neoplasia differentially expressed (non-protein coding)* | 4.88E-03 | -1.95 |
| NM_001137550 /// NM_001137551 /// NM_001137552 /// NM_001137553 /// NM_004735 | *LRRFIP1* | | | | *leucine rich repeat (in FLII) interacting protein 1* | 4.79E-02 | -1.96 |
| NM_003498 | *SNN* | | | | *stannin* | 3.89E-04 | -1.96 |
| NM_006617 | *NES* | | | | *nestin* | 3.78E-02 | -1.97 |
| NM_001278312 /// NM_004298 /// NM_153485 | *NUP155* | | | | *nucleoporin 155kDa* | 2.96E-02 | -1.97 |
| NM_000179 | *MSH6* | | | | *mutS homolog 6 (E. coli)* | 3.99E-02 | -1.97 |
| NM_198566 | *C5orf34* | | | | *chromosome 5 open reading frame 34* | 4.31E-02 | -1.97 |
| NM_001037164 /// NM_001195396 /// NM_005738 /// NM_212460 | *ARL4A* | | | | *ADP-ribosylation factor-like 4A* | 4.62E-02 | -1.99 |
| NM_152683 | *CCDC111* | | | | *coiled-coil domain containing 111* | 1.02E-02 | -1.99 |
| NM_002742 | *PRKD1* | | | | *protein kinase D1* | 3.36E-02 | -2.00 |
| NM_001243723 /// NM_001276325 /// NM_006515 /// NR_024022 /// NR_075073 | *SETMAR* | | | | *SET domain and mariner transposase fusion gene* | 2.68E-03 | -2.00 |
| NM_005923 | *MAP3K5* | | | | *mitogen-activated protein kinase kinase kinase 5* | 1.83E-02 | -2.00 |
| NM_006716 | *DBF4* | | | | *DBF4 homolog (S. cerevisiae)* | 1.77E-02 | -2.01 |
| NM_005483 | *CHAF1A* | | | | *chromatin assembly factor 1, subunit A (p150)* | 6.67E-03 | -2.02 |
| NM_001100417 /// NM_175748 /// NR_038150 | *UBR7* | | | | *ubiquitin protein ligase E3 component n-recognin 7 (putative)* | 3.55E-02 | -2.02 |
| NM_001204300 /// NM_001256423 /// NM_032900 | *ARHGAP19* | | | | *Rho GTPase activating protein 19* | 3.93E-02 | -2.02 |
| NM_001080383 /// NM_005497 | *GJC1* | | | | *gap junction protein, gamma 1, 45kDa* | 4.18E-03 | -2.03 |
| NM_003920 | *TIMELESS* | | | | *timeless circadian clock* | 4.50E-02 | -2.03 |
| NM_001168551 /// NM_018087 /// NR_033142 | *NDC1* | | | | *NDC1 transmembrane nucleoporin* | 3.00E-02 | -2.03 |
| NM_001113411 /// NM_001244714 /// NM_001278224 /// NM_018291 /// NR_103473 | *FGGY* | | | | *FGGY carbohydrate kinase domain containing* | 2.18E-02 | -2.04 |
| NM_001270467 /// NM_001270468 /// NM_001270469 /// NM_001270470 /// NM_001270471 | *SOCS2* | | | | *suppressor of cytokine signaling 2* | 8.51E-03 | -2.04 |
| NM_001018113 /// NM_152633 | *FANCB* | | | | *Fanconi anemia, complementation group B* | 3.32E-02 | -2.04 |
| NM_002689 | *POLA2* | | | | *polymerase (DNA directed), alpha 2, accessory subunit* | 2.06E-02 | -2.05 |
| NM_001135821 /// NM_001135822 /// NM_001242824 /// NM_001242825 /// NM_002004 | *FDPS* | | | | *farnesyl diphosphate synthase* | 1.09E-02 | -2.05 |
| NM_020147 | *THAP10* | | | | *THAP domain containing 10* | 2.09E-02 | -2.06 |
| NM_001166252 /// NM_014839 | *LPPR4* | | | | *lipid phosphate phosphatase-related protein type 4* | 3.66E-02 | -2.07 |
| NM_001167865 /// NM_017615 | *NSMCE4A* | | | | *non-SMC element 4 homolog A (S. cerevisiae)* | 4.90E-02 | -2.07 |
| NM_001195193 /// NM_002482 /// NM_152298 /// NM_172164 | *NASP* | | | | *nuclear autoantigenic sperm protein (histone-binding)* | 2.85E-02 | -2.08 |
| NM_017915 | *PARPBP* | | | | *PARP1 binding protein* | 3.19E-02 | -2.09 |
| NM_013258 /// NM_145182 /// NM_145183 | *PYCARD* | | | | *PYD and CARD domain containing* | 2.67E-02 | -2.09 |
| NM_001798 /// NM_052827 | *CDK2* | | | | *cyclin-dependent kinase 2* | 3.19E-02 | -2.10 |
| NM_002168 | *IDH2* | | | | *isocitrate dehydrogenase 2 (NADP+), mitochondrial* | 5.71E-03 | -2.10 |
| NM_001131005 /// NM_001193347 /// NM_001193348 /// NM_001193349 /// NM_001193350 | *MEF2C* | | | | *myocyte enhancer factor 2C* | 5.33E-03 | -2.11 |
| NM_001256012 /// NM_001256095 /// NM_005964 | *MYH10* | | | | *myosin, heavy chain 10, non-muscle* | 4.35E-02 | -2.12 |
| NM_001042459 /// NM_014890 /// NM_182909 | *FILIP1L* | | | | *filamin A interacting protein 1-like* | 1.28E-03 | -2.12 |
| NM_001012968 | *SPIN4* | | | | *spindlin family, member 4* | 3.84E-02 | -2.13 |
| NM_024491 | *CEP70* | | | | *centrosomal protein 70kDa* | 3.67E-03 | -2.13 |
| NM_001042476 /// NM_001278260 /// NM_001278261 /// NM_001278262 /// NM_001278263 | *CARHSP1* | | | | *calcium regulated heat stable protein 1, 24kDa* | 6.53E-04 | -2.14 |
| NM_024590 | *ARSJ* | | | | *arylsulfatase family, member J* | 3.46E-02 | -2.14 |
| NM_000961 | *PTGIS* | | | | *prostaglandin I2 (prostacyclin) synthase* | 7.47E-03 | -2.15 |
| NM_198468 | *MMS22L* | | | | *MMS22-like, DNA repair protein* | 4.16E-03 | -2.15 |
| NM_001145453 /// NM_005264 /// NM_145793 | *GFRA1* | | | | *GDNF family receptor alpha 1* | 3.32E-02 | -2.16 |
| NM_000251 /// NM_001258281 | *MSH2* | | | | *mutS homolog 2, colon cancer, nonpolyposis type 1 (E. coli)* | 1.96E-02 | -2.17 |
| NM_001100624 /// NM_001100625 /// NM_001270473 /// NM_001270474 /// NM_018455 | *CENPN* | | | | *centromere protein N* | 4.20E-02 | -2.17 |
| NM_004120 | *GBP2* | | | | *guanylate binding protein 2, interferon-inducible* | 2.44E-02 | -2.17 |
| NM_001178130 /// NM_001178131 /// NM_001963 | *EGF* | | | | *epidermal growth factor* | 1.19E-02 | -2.18 |
| NM_021020 | *LZTS1* | | | | *leucine zipper, putative tumor suppressor 1* | 1.58E-02 | -2.18 |
| NM_024336 | *IRX3* | | | | *iroquois homeobox 3* | 2.17E-03 | -2.18 |
| NM_001099652 | *GPR137C* | | | | *G protein-coupled receptor 137C* | 1.78E-02 | -2.19 |
| NM_001194998 /// NM_014985 | *CEP152* | | | | *centrosomal protein 152kDa* | 2.01E-02 | -2.19 |
| NM_001033044 /// NM_001033056 /// NM_002065 | *GLUL* | | | | *glutamate-ammonia ligase* | 4.84E-02 | -2.19 |
| NM_004508 | *IDI1* | | | | *isopentenyl-diphosphate delta isomerase 1* | 3.30E-02 | -2.20 |
| NM_001114121 /// NM_001114122 /// NM_001244846 /// NM_001274 /// NR_045204 /// NR_04520 | *CHEK1* | | | | *checkpoint kinase 1* | 4.50E-02 | -2.20 |
| NM_001005476 /// NM_003628 | *PKP4* | | | | *plakophilin 4* | 4.69E-04 | -2.20 |
| NM_018214 /// NM_025168 | *LRRC1* | | | | *leucine rich repeat containing 1* | 3.72E-02 | -2.20 |
| NM_024037 | *AUNIP* | | | | *aurora kinase A and ninein interacting protein* | 9.97E-05 | -2.21 |
| NM_153332 | *ERI1* | | | | *exoribonuclease 1* | 2.70E-02 | -2.23 |
| NM_005914 /// NM_182746 | *MCM4* | | | | *minichromosome maintenance complex component 4* | 2.70E-02 | -2.23 |
| NM_001135805 /// NM_001135806 /// NM_005639 | *SYT1* | | | | *synaptotagmin I* | 4.82E-02 | -2.23 |
| NM_001105533 /// NM_024913 | *CPED1* | | | | *cadherin-like and PC-esterase domain containing 1* | 1.52E-03 | -2.23 |
| NM_024945 | *RMI1* | | | | *RecQ mediated genome instability 1* | 2.52E-02 | -2.24 |
| NM_001078166 /// NM_006924 /// NR_034041 | *SRSF1* | | | | *serine/arginine-rich splicing factor 1* | 1.54E-02 | -2.24 |
| NM_003940 | *USP13* | | | | *ubiquitin specific peptidase 13 (isopeptidase T-3)* | 3.02E-02 | -2.24 |
| NM_001097633 /// NM_001097634 /// NM_001097635 /// NM_001097636 /// NM_001490 | | *GCNT1* | | | *glucosaminyl (N-acetyl) transferase 1, core 2* | 4.72E-03 | -2.25 |
| NM_001178096 /// NM_001993 | *F3* | | | | *coagulation factor III (thromboplastin, tissue factor)* | 4.25E-02 | -2.25 |
| NM_001193424 /// NM_001193425 /// NM_001193426 /// NM_001193427 /// NM_024670 | *SUV39H2* | | | | *suppressor of variegation 3-9 homolog 2 (Drosophila)* | 3.23E-02 | -2.25 |
| NM_017785 | *SPDL1* | | | | *spindle apparatus coiled-coil protein 1* | 9.04E-03 | -2.26 |
| NM_020165 | *RAD18* | | | | *RAD18 homolog (S. cerevisiae)* | 3.30E-02 | -2.26 |
| NM_017955 /// NM_145701 | *CDCA4* | | | | *cell division cycle associated 4* | 4.28E-02 | -2.27 |
| NM_001142548 /// NM_003579 | *RAD54L* | | | | *RAD54-like (S. cerevisiae)* | 3.96E-02 | -2.29 |
| NM_000965 /// NM_016152 | *RARB* | | | | *retinoic acid receptor, beta* | 9.46E-03 | -2.29 |
| NM_022490 | *POLR1E* | | | | *polymerase (RNA) I polypeptide E, 53kDa* | 1.49E-02 | -2.30 |
| NM_001033044 /// NM_001033056 /// NM_002065 | *GLUL* | | | | *glutamate-ammonia ligase* | 1.57E-02 | -2.31 |
| NM_014573 | *TMEM97* | | | | *transmembrane protein 97* | 4.07E-02 | -2.32 |
| NM_001004126 /// NM_031218 | *ZNF93* | | | | *zinc finger protein 93* | 9.01E-03 | -2.32 |
| NM_001017369 /// NM_006745 | *MSMO1* | | | | *methylsterol monooxygenase 1* | 4.82E-02 | -2.32 |
| NM_001098272 /// NM_002130 | *HMGCS1* | | | | *3-hydroxy-3-methylglutaryl-CoA synthase 1 (soluble)* | 1.66E-02 | -2.32 |
| NM_014505 | *KCNMB4* | | | | *potassium large conductance calcium-activated channel, subfamily M, beta member 4* | 2.43E-02 | -2.33 |
| NM_001080416 /// NM_001144755 | *MYBL1* | | | | *v-myb myeloblastosis viral oncogene homolog (avian)-like 1* | 3.98E-02 | -2.34 |
| NM_032290 | *ANKRD32* | | | | *ankyrin repeat domain 32* | 1.46E-03 | -2.35 |
| NM_018944 | *MIS18A* | | | | *MIS18 kinetochore protein homolog A (S. pombe)* | 4.57E-02 | -2.35 |
| NM_018451 /// NR_047594 /// NR_047595 | *CENPJ* | | | | *centromere protein J* | 3.01E-02 | -2.35 |
| NM_002266 /// NR_026899 /// NR_027487 | *KPNA2 /// LOC146880* | | | | *karyopherin alpha 2 (RAG cohort 1, importin alpha 1) /// Rho GTPase activating protein* | 3.61E-02 | -2.36 |
| NM_178448 | *SAPCD2* | | | | *suppressor APC domain containing 2* | 2.88E-02 | -2.36 |
| NM_001143824 /// NM_018018 | *SLC38A4* | | | | *solute carrier family 38, member 4* | 2.78E-02 | -2.36 |
| NM_022836 | *DCLRE1B* | | | | *DNA cross-link repair 1B* | 1.02E-02 | -2.37 |
| NM_000683 | *ADRA2C* | | | | *adrenoceptor alpha 2C* | 9.02E-03 | -2.37 |
| NM_005139 | *ANXA3* | | | | *annexin A3* | 3.96E-02 | -2.37 |
| NM_024900 /// NM_199320 | *PHF17* | | | | *PHD finger protein 17* | 1.64E-02 | -2.38 |
| NM_133459 | *CCBE1* | | | | *collagen and calcium binding EGF domains 1* | 3.90E-02 | -2.39 |
| NM_016235 | *GPRC5B* | | | | *G protein-coupled receptor, family C, group 5, member B* | 1.69E-02 | -2.39 |
| NM_002193 | *INHBB* | | | | *inhibin, beta B* | 1.78E-02 | -2.42 |
| NM_000857 | *GUCY1B3* | | | | *guanylate cyclase 1, soluble, beta 3* | 2.22E-03 | -2.42 |
| NM_020859 | *SHROOM3* | | | | *shroom family member 3* | 3.78E-02 | -2.42 |
| NM_001008544 /// NM_138285 | *NUP35* | | | | *nucleoporin 35kDa* | 1.37E-02 | -2.43 |
| NM_001145454 /// NM_005563 /// NM_152497 /// NM_203399 /// NM_203401 | *STMN1* | | | | *stathmin 1* | 2.33E-02 | -2.44 |
| NM_001135554 /// NM_001135555 /// NM_001199388 /// NM_001199389 /// NM_001252660 | | *EPB41L2* | | | *erythrocyte membrane protein band 4.1-like 2* | 4.56E-03 | -2.44 |
| NM_001134709 /// NM_003472 | *DEK* | | | | *DEK oncogene* | 4.33E-02 | -2.44 |
| NM_001130963 /// NM_015257 | *TMEM194A* | | | | *transmembrane protein 194A* | 2.27E-02 | -2.45 |
| NM_000222 /// NM_001093772 | *KIT* | | | | *v-kit Hardy-Zuckerman 4 feline sarcoma viral oncogene homolog* | 4.75E-04 | -2.45 |
| NM_001236 | *CBR3* | | | | *carbonyl reductase 3* | 2.30E-02 | -2.45 |
| NM_001171195 /// NM_001171197 /// NM_004432 | *ELAVL2* | | | | *ELAV (embryonic lethal, abnormal vision, Drosophila)-like 2 (Hu antigen B)* | 1.31E-03 | -2.46 |
| NM_000800 /// NM_001144892 /// NM_001144934 /// NM_001144935 /// NM_001257205 | | *FGF1* | | | *fibroblast growth factor 1 (acidic)* | 4.12E-02 | -2.46 |
| NM_032737 | *LMNB2* | | | | *lamin B2* | 4.82E-02 | -2.46 |
| NM_014708 | *KNTC1* | | | | *kinetochore associated 1* | 3.41E-02 | -2.47 |
| NM_001012409 /// NM_001012410 /// NM_001012411 /// NM_001012412 /// NM_001012413 | | *SGOL1* | | | *shugoshin-like 1 (S. pombe)* | 3.22E-02 | -2.47 |
| NM_017769 | *G2E3* | | | | *G2/M-phase specific E3 ubiquitin protein ligase* | 1.80E-02 | -2.50 |
| NM_006591 /// NR_046409 /// NR_046410 | *POLD3* | | | | *polymerase (DNA-directed), delta 3, accessory subunit* | 1.46E-02 | -2.51 |
| NM_005055 /// NM_032645 | *RAPSN* | | | | *receptor-associated protein of the synapse* | 2.21E-02 | -2.52 |
| NM_001145102 /// NM_001145103 /// NM_001145104 /// NM_005902 | *SMAD3* | | | | *SMAD family member 3* | 2.70E-03 | -2.54 |
| NM_001256269 /// NM_001256270 /// NM_007317 | *KIF22* | | | | *kinesin family member 22* | 1.19E-02 | -2.54 |
| NM_002296 /// NM_194442 | *LBR* | | | | *lamin B receptor* | 2.13E-02 | -2.55 |
| NM_001199893 /// NM_001615 | *ACTG2* | | | | *actin, gamma 2, smooth muscle, enteric* | 1.78E-02 | -2.55 |
| NM_002852 | *PTX3* | | | | *pentraxin 3, long* | 1.36E-02 | -2.56 |
| NM_001102654 /// NM_002527 | *NTF3* | | | | *neurotrophin 3* | 3.47E-02 | -2.58 |
| NM_152308 | *RMI2* | | | | *RecQ mediated genome instability 2* | 7.60E-03 | -2.59 |
| NM_001164269 /// NM_001164270 /// NM_002875 /// NM_133487 | *RAD51* | | | | *RAD51 homolog (S. cerevisiae)* | 3.81E-02 | -2.59 |
| NM_178431 | *LCE3A* | | | | *late cornified envelope 3A* | 3.52E-02 | -2.61 |
| NM_001190481 /// NM_022111 | *CLSPN* | | | | *claspin* | 3.95E-02 | -2.61 |
| NM_017760 | *NCAPG2* | | | | *non-SMC condensin II complex, subunit G2* | 4.96E-02 | -2.64 |
| NM_001130146 /// NM_001130147 /// NM_001130148 /// NM_032358 | *CCDC77* | | | | *coiled-coil domain containing 77* | 2.09E-02 | -2.64 |
| NM_178496 | *MB21D2* | | | | *Mab-21 domain containing 2* | 4.55E-02 | -2.64 |
| NM_002916 /// NM_181573 | *RFC4* | | | | *replication factor C (activator 1) 4, 37kDa* | 2.04E-02 | -2.64 |
| NM_000050 /// NM_054012 | *ASS1* | | | | *argininosuccinate synthase 1* | 1.45E-02 | -2.66 |
| NM_007294 /// NM_007295 /// NM_007296 /// NM_007297 /// NM_007298 /// NM_007299 | *BRCA1* | | | | *breast cancer 1, early onset* | 2.42E-02 | -2.68 |
| NM_002469 | *MYF6* | | | | *myogenic factor 6 (herculin)* | 3.45E-02 | -2.72 |
| NM_001258315 /// NM_001258316 /// NM_018098 | *ECT2* | | | | *epithelial cell transforming sequence 2 oncogene* | 2.15E-02 | -2.72 |
| NM_001114173 /// NM_001814 /// NM_148170 | *CTSC* | | | | *cathepsin C* | 3.96E-03 | -2.74 |
| NM_001098802 /// NM_032171 | *CEP78* | | | | *centrosomal protein 78kDa* | 1.34E-02 | -2.74 |
| NM_001827 | *CKS2* | | | | *CDC28 protein kinase regulatory subunit 2* | 1.75E-02 | -2.77 |
| NM_001185056 /// NM_005602 | *CLDN11* | | | | *claudin 11* | 3.89E-02 | -2.77 |
| NM_001635 /// NM_139316 | *AMPH* | | | | *amphiphysin* | 1.35E-02 | -2.82 |
| NM_001042424 /// NM_007331 /// NM_014919 /// NM_133330 /// NM_133331 /// NM_133332 | *WHSC1* | | | | *Wolf-Hirschhorn syndrome candidate 1* | 3.48E-02 | -2.83 |
| NM_005342 | *HMGB3* | | | | *high mobility group box 3* | 1.92E-02 | -2.84 |
| NM_005891 | *ACAT2* | | | | *acetyl-CoA acetyltransferase 2* | 4.55E-02 | -2.84 |
| NM_001251989 /// NM_001251990 /// NM_001251991 /// NM_015895 | *GMNN* | | | | *geminin, DNA replication inhibitor* | 1.07E-02 | -2.85 |
| NM_001826 /// NR_024163 | *CKS1B* | | | | *CDC28 protein kinase regulatory subunit 1B* | 4.25E-02 | -2.85 |
| NM_001321 | *CSRP2* | | | | *cysteine and glycine-rich protein 2* | 7.65E-03 | -2.89 |
| NM_021998 | *ZNF711* | | | | *zinc finger protein 711* | 2.97E-02 | -2.90 |
| NM_000947 | *PRIM2* | | | | *primase, DNA, polypeptide 2 (58kDa)* | 7.29E-03 | -2.90 |
| NM_001142761 /// NM_001142762 /// NM_033286 | *KNSTRN* | | | | *kinetochore-localized astrin/SPAG5 binding protein* | 3.47E-02 | -2.91 |
| NM_001142393 /// NM_001271033 /// NM_006403 /// NM_182966 /// NR_073131 | *NEDD9* | | | | *neural precursor cell expressed, developmentally down-regulated 9* | 1.61E-02 | -2.91 |
| NM_001009936 /// NM_015651 | *PHF19* | | | | *PHD finger protein 19* | 2.51E-02 | -2.92 |
| NM_018132 | *CENPQ* | | | | *centromere protein Q* | 2.33E-02 | -2.94 |
| NM_001002799 /// NM_001002800 /// NM_005496 | *SMC4* | | | | *structural maintenance of chromosomes 4* | 4.20E-02 | -2.95 |
| NM_001042550 /// NM_001042551 /// NM_001265602 /// NM_006444 | *SMC2* | | | | *structural maintenance of chromosomes 2* | 4.90E-02 | -2.95 |
| NM_001005290 /// NM_001032290 /// NM_001032291 /// NM_032636 | *PSRC1* | | | | *proline/serine-rich coiled-coil 1* | 2.33E-02 | -2.98 |
| NM_173529 | *C18orf54* | | | | *chromosome 18 open reading frame 54* | 7.89E-03 | -3.00 |
| NM_001098525 /// NM_018204 | *CKAP2* | | | | *cytoskeleton associated protein 2* | 9.27E-03 | -3.02 |
| NM_004526 /// NR_073375 | *MCM2* | | | | *minichromosome maintenance complex component 2* | 2.05E-02 | -3.03 |
| NM_004669 | *CLIC3* | | | | *chloride intracellular channel 3* | 1.98E-02 | -3.06 |
| NM_001127181 /// NM_001171182 /// NM_033319 | *CENPL* | | | | *centromere protein L* | 3.08E-02 | -3.07 |
| NM_014109 | *ATAD2* | | | | *ATPase family, AAA domain containing 2* | 1.17E-02 | -3.08 |
| NM_002478 | *MYOD1* | | | | *myogenic differentiation 1* | 1.28E-02 | -3.08 |
| NM_001256014 /// NM_001256015 /// NM_001256016 /// NM_013290 /// NM_016556 | *PSMC3IP* | | | | *PSMC3 interacting protein* | 2.06E-02 | -3.08 |
| NM_015351 | *TTC9* | | | | *tetratricopeptide repeat domain 9* | 9.51E-03 | -3.09 |
| NM_005517 | *HMGN2* | | | | *high mobility group nucleosomal binding domain 2* | 2.89E-02 | -3.15 |
| NM_001114133 /// NM_024875 | *SYNPO2L* | | | | *synaptopodin 2-like* | 4.58E-02 | -3.18 |
| NM_016937 | *POLA1* | | | | *polymerase (DNA directed), alpha 1, catalytic subunit* | 3.36E-02 | -3.20 |
| NM_001143976 /// NM_003390 | *WEE1* | | | | *WEE1 homolog (S. pombe)* | 1.82E-02 | -3.21 |
| NM_001034077 /// NM_003495 /// NM_003538 | *HIST1H4A /// HIST1H4B /// HIST1H4I* | | | | *histone cluster 1, H4a /// histone cluster 1, H4b /// histone cluster 1, H4i* | 2.49E-02 | -3.22 |
| NM_004418 | *DUSP2* | | | | *dual specificity phosphatase 2* | 1.01E-02 | -3.27 |
| NM_015441 | *OLFML2B* | | | | *olfactomedin-like 2B* | 7.72E-03 | -3.29 |
| NM_024094 | *DSCC1* | | | | *DNA replication and sister chromatid cohesion 1* | 1.82E-02 | -3.29 |
| NM_025049 | *PIF1* | | | | *PIF1 5'-to-3' DNA helicase* | 1.03E-02 | -3.31 |
| NM_020890 | *KIAA1524* | | | | *KIAA1524* | 6.61E-03 | -3.36 |
| NM_001017415 /// NM_001017416 /// NM_003368 | *USP1* | | | | *ubiquitin specific peptidase 1* | 3.04E-02 | -3.50 |
| NM_001142519 /// NM_001142520 /// NM_001142521 /// NM_022074 /// NM_198847 | | *FAM111A* | | | *family with sequence similarity 111, member A* | 1.10E-02 | -3.50 |
| NM_001009954 /// NM_017669 | *ERCC6L* | | | | *excision repair cross-complementing rodent repair deficiency, complementation group 6-l* | 2.04E-02 | -3.53 |
| NM_002105 | *H2AFX* | | | | *H2A histone family, member X* | 2.81E-02 | -3.53 |
| NM_014762 | *DHCR24* | | | | *24-dehydrocholesterol reductase* | 4.65E-02 | -3.56 |
| NM_014176 | *UBE2T* | | | | *ubiquitin-conjugating enzyme E2T (putative)* | 4.52E-02 | -3.61 |
| NM_014865 | *NCAPD2* | | | | *non-SMC condensin I complex, subunit D2* | 2.73E-02 | -3.61 |
| NM_001008393 /// NR_077234 /// NR_077235 | *C4orf46* | | | | *chromosome 4 open reading frame 46* | 6.43E-03 | -3.61 |
| NM_014783 /// NM_199357 | *ARHGAP11A* | | | | *Rho GTPase activating protein 11A* | 1.55E-03 | -3.63 |
| NM_001256685 /// NM_001256687 /// NM_001256688 /// NM_001256689 /// NM_001256690 | | *MELK* | | | *maternal embryonic leucine zipper kinase* | 3.25E-02 | -3.63 |
| NM_173084 | *TRIM59* | | | | *tripartite motif containing 59* | 2.62E-03 | -3.71 |
| NM_000916 | *OXTR* | | | | *oxytocin receptor* | 1.42E-02 | -3.72 |
| NM_001884 | *HAPLN1* | | | | *hyaluronan and proteoglycan link protein 1* | 2.21E-02 | -3.73 |
| NM_001032283 /// NM_001032284 /// NM_003276 | *TMPO* | | | | *thymopoietin* | 1.86E-02 | -3.78 |
| NM_001127370 /// NM_001127371 /// NM_018719 | *CDCA7L* | | | | *cell division cycle associated 7-like* | 1.64E-02 | -3.78 |
| NM_001253861 /// NM_032117 /// NR_045605 | *MND1* | | | | *meiotic nuclear divisions 1 homolog (S. cerevisiae)* | 2.52E-02 | -3.78 |
| NM_001790 /// NM_022809 | *CDC25C* | | | | *cell division cycle 25C* | 2.23E-02 | -3.82 |
| NM_021979 | *HSPA2* | | | | *heat shock 70kDa protein 2* | 2.24E-02 | -3.88 |
| NM_001761 | *CCNF* | | | | *cyclin F* | 3.24E-02 | -3.91 |
| NM_018424 /// NM_019114 | *EPB41L4B* | | | | *erythrocyte membrane protein band 4.1 like 4B* | 2.48E-02 | -3.92 |
| NM_024629 | *MLF1IP* | | | | *MLF1 interacting protein* | 1.27E-02 | -3.92 |
| NM_013296 | *GPSM2* | | | | *G-protein signaling modulator 2* | 2.80E-02 | -3.93 |
| NM_174942 | *GAS2L3* | | | | *growth arrest-specific 2 like 3* | 4.22E-02 | -3.97 |
| NM_005593 | *MYF5* | | | | *myogenic factor 5* | 2.29E-02 | -3.97 |
| NM_145018 | *C11orf82* | | | | *chromosome 11 open reading frame 82* | 4.14E-02 | -4.01 |
| NM_000946 | *PRIM1* | | | | *primase, DNA, polypeptide 1 (49kDa)* | 4.56E-02 | -4.06 |
| NM_001256834 /// NM_004217 | *AURKB* | | | | *aurora kinase B* | 4.33E-02 | -4.06 |
| NM_001126103 /// NM_001126104 /// NM_013277 | *RACGAP1* | | | | *Rac GTPase activating protein 1* | 4.07E-02 | -4.08 |
| NM_005916 /// NM_182776 | *MCM7* | | | | *minichromosome maintenance complex component 7* | 3.39E-02 | -4.27 |
| NM_020242 | *KIF15* | | | | *kinesin family member 15* | 7.14E-03 | -4.28 |
| NM_001254 | *CDC6* | | | | *cell division cycle 6* | 1.15E-02 | -4.30 |
| NM_001011699 /// NM_033417 | *HAUS8* | | | | *HAUS augmin-like complex, subunit 8* | 4.88E-02 | -4.32 |
| NM_018063 | *HELLS* | | | | *helicase, lymphoid-specific* | 2.49E-02 | -4.36 |
| NM_001203247 /// NM_001203248 /// NM_001203249 /// NM_004456 /// NM_152998 | | *EZH2* | | | *enhancer of zeste homolog 2 (Drosophila)* | 1.30E-02 | -4.38 |
| NM_153695 | *ZNF367* | | | | *zinc finger protein 367* | 3.12E-02 | -4.42 |
| NM_001262 /// NM_078626 | *CDKN2C* | | | | *cyclin-dependent kinase inhibitor 2C (p18, inhibits CDK4)* | 9.76E-03 | -4.44 |
| NM_016448 | *DTL* | | | | *denticleless E3 ubiquitin protein ligase homolog (Drosophila)* | 3.94E-02 | -4.45 |
| NM_001002876 /// NM_001110215 /// NM_024053 | *CENPM* | | | | *centromere protein M* | 3.02E-02 | -4.47 |
| NM_001130862 /// NM_006479 | *RAD51AP1* | | | | *RAD51 associated protein 1* | 2.53E-02 | -4.48 |
| NM_001008568 /// NM_001008570 | *TRMU* | | | | *tRNA 5-methylaminomethyl-2-thiouridylate methyltransferase* | 2.52E-02 | -4.61 |
| NM_002358 | *MAD2L1* | | | | *MAD2 mitotic arrest deficient-like 1 (yeast)* | 4.21E-02 | -4.67 |
| NM_001077511 /// NM_007109 | *TCF19* | | | | *transcription factor 19* | 2.26E-02 | -4.67 |
| NM_021067 | *GINS1* | | | | *GINS complex subunit 1 (Psf1 homolog)* | 3.41E-02 | -4.69 |
| NM_001048201 /// NM_013282 | *UHRF1* | | | | *ubiquitin-like with PHD and ring finger domains 1* | 4.41E-02 | -4.71 |
| NM_199420 | *POLQ* | | | | *polymerase (DNA directed), theta* | 4.79E-02 | -4.84 |
| NM_018518 /// NM_182751 | *MCM10* | | | | *minichromosome maintenance complex component 10* | 3.54E-02 | -4.88 |
| NM_182513 | *SPC24* | | | | *SPC24, NDC80 kinetochore complex component* | 3.48E-02 | -4.90 |
| NM_031217 | *KIF18A* | | | | *kinesin family member 18A* | 4.51E-02 | -5.06 |
| NM_080668 | *CDCA5* | | | | *cell division cycle associated 5* | 2.80E-02 | -5.29 |
| NM_001130829 /// NM_001170406 /// NM_001170407 /// NM_001786 /// NM_033379 | *CDK1* | | | | *cyclin-dependent kinase 1* | 3.00E-02 | -5.29 |
| NM_006342 | *TACC3* | | | | *transforming, acidic coiled-coil containing protein 3* | 1.88E-02 | -5.31 |
| NM_001160033 /// NM_001160046 /// NM_152524 | *SGOL2* | | | | *shugoshin-like 2 (S. pombe)* | 2.62E-02 | -5.35 |
| NM_001129897 /// NM_001243142 /// NM_001243143 /// NM_001243144 /// NM_016359 | | *NUSAP1* | | | *nucleolar and spindle associated protein 1* | 3.08E-02 | -5.36 |
| NM_005259 | *MSTN* | | | | *myostatin* | 3.24E-02 | -5.46 |
| NM_016343 | *CENPF* | | | | *centromere protein F, 350/400kDa* | 2.89E-02 | -5.47 |
| NM_016095 | *GINS2* | | | | *GINS complex subunit 2 (Psf2 homolog)* | 3.50E-02 | -5.55 |
| NM_001145966 /// NM_002417 | *MKI67* | | | | *antigen identified by monoclonal antibody Ki-67* | 2.72E-02 | -5.61 |
| NM_031966 | *CCNB1* | | | | *cyclin B1* | 3.05E-02 | -5.65 |
| NM_012414 | *RAB3GAP2* | | | | *RAB3 GTPase activating protein sub* | 3.55E-02 | -5.67 |
| NM_152515 | *CKAP2L* | | | | *cytoskeleton associated protein 2-like* | 3.68E-02 | -5.67 |
| NM_001012270 /// NM_001012271 /// NM_001168 | *BIRC5* | | | | *baculoviral IAP repeat containing 5* | 2.15E-02 | -5.69 |
| NM_006461 | *SPAG5* | | | | *sperm associated antigen 5* | 1.92E-02 | -5.71 |
| NM_001130688 /// NM_001130689 /// NM_002129 | *HMGB2* | | | | *high mobility group box 2* | 2.31E-02 | -5.83 |
| NM_001017420 | *ESCO2* | | | | *establishment of sister chromatid cohesion N-acetyltransferase 2* | 4.04E-02 | -5.84 |
| NM_001262 /// NM_078626 | *CDKN2C* | | | | *cyclin-dependent kinase inhibitor 2C (p18, inhibits CDK4)* | 2.05E-02 | -5.86 |
| NM_152562 | *CDCA2* | | | | *cell division cycle associated 2* | 2.09E-02 | -5.92 |
| NM_144508 /// NM_170589 | *CASC5* | | | | *cancer susceptibility candidate 5* | 2.07E-02 | -6.04 |
| NM_001142522 /// NM_012177 | *FBXO5* | | | | *F-box protein 5* | 1.83E-02 | -6.04 |
| NM_001130829 /// NM_001170406 /// NM_001170407 /// NM_001786 /// NM_033379 | | *CDK1* | | | *cyclin-dependent kinase 1* | 4.47E-02 | -6.10 |
| NM_016426 | *GTSE1* | | | | *G-2 and S-phase expressed 1* | 1.82E-02 | -6.18 |
| NM_018410 | *HJURP* | | | | *Holliday junction recognition protein* | 4.71E-02 | -6.22 |
| NM_001048166 /// NM_003035 | *STIL* | | | | *SCL/TAL1 interrupting locus* | 4.82E-02 | -6.28 |
| NM_015341 | *NCAPH* | | | | *non-SMC condensin I complex, subunit H* | 3.53E-02 | -6.31 |
| NM_007280 | *OIP5* | | | | *Opa interacting protein 5* | 1.56E-02 | -6.36 |
| NM_000527 /// NM_001195798 /// NM_001195799 /// NM_001195800 | | *LDLR* | | | *low density lipoprotein receptor* | 2.50E-02 | -6.49 |
| NM_007019 /// NM_181799 /// NM_181800 /// NM_181801 /// NM_181802 /// NM_181803 | *UBE2C* | | | | *ubiquitin-conjugating enzyme E2C* | 2.30E-02 | -6.57 |
| NM_014875 | *KIF14* | | | | *kinesin family member 14* | 2.36E-02 | -6.63 |
| NM_018492 | *PBK* | | | | *PDZ binding kinase* | 4.50E-02 | -6.63 |
| NM_012112 | *TPX2* | | | | *TPX2, microtubule-associated, homolog (Xenopus laevis)* | 3.33E-02 | -6.63 |
| NM_001198557 /// NM_005573 | *LMNB1* | | | | *lamin B1* | 3.56E-02 | -6.81 |
| NM_001067 | *TOP2A* | | | | *topoisomerase (DNA) II alpha 170kDa* | 2.58E-02 | -6.95 |
| NM_004702 /// NM_057735 /// NM_057749 | *CCNE2* | | | | *cyclin E2* | 2.79E-02 | -6.96 |
| NM_018685 | *ANLN* | | | | *anillin, actin binding protein* | 1.53E-02 | -6.97 |
| NM_031423 /// NM_145697 | *NUF2* | | | | *NUF2, NDC80 kinetochore complex component, homolog (S. cerevisiae)* | 2.61E-02 | -7.05 |
| NM_001039535 /// NM_145060 | *SKA1* | | | | *spindle and kinetochore associated complex subunit 1* | 1.55E-02 | -7.23 |
| NM_031942 /// NM_145810 | *CDCA7* | | | | *cell division cycle associated 7* | 1.96E-02 | -7.24 |
| NM_001142556 /// NM_001142557 /// NM_012484 /// NM_012485 | | *HMMR* | | | *hyaluronan-mediated motility receptor (RHAMM)* | 5.04E-03 | -7.62 |
| NM_012310 | *KIF4A* | | | | *kinesin family member 4A* | 3.80E-02 | -7.63 |
| NM_001237 | *CCNA2* | | | | *cyclin A2* | 2.14E-02 | -7.71 |
| NM_001114120 /// NM_017779 | *DEPDC1* | | | | *DEP domain containing 1* | 4.00E-03 | -7.84 |
| NM_000465 | *BARD1* | | | | *BRCA1 associated RING domain 1* | 5.14E-03 | -7.93 |
| NM_004523 | *KIF11* | | | | *kinesin family member 11* | 3.61E-02 | -8.45 |
| NM_001127182 /// NM_018131 | *CEP55* | | | | *centrosomal protein 55kDa* | 2.10E-02 | -8.49 |
| NM_001256875 /// NM_018101 | *CDCA8* | | | | *cell division cycle associated 8* | 2.21E-02 | -8.69 |
| NM_031299 | *CDCA3* | | | | *cell division cycle associated 3* | 9.07E-03 | -8.74 |
| NM_001099293 | *KIF4B* | | | | *kinesin family member 4B* | 1.25E-02 | -8.97 |
| NM_001142651 /// NR_015355 | *NEURL1B* | | | | *neuralized homolog 1B (Drosophila)* | 3.00E-02 | -9.29 |
| NM_004856 /// NM_138555 | *KIF23* | | | | *kinesin family member 23* | 2.28E-02 | -9.36 |
| NM_004701 | *CCNB2* | | | | *cyclin B2* | 2.33E-02 | -9.37 |
| NM_001145208 /// NM_018369 | *DEPDC1B* | | | | *DEP domain containing 1B* | 1.43E-02 | -9.54 |
| NM_001255 | *CDC20* | | | | *cell division cycle 20* | 1.14E-02 | -9.59 |
| NM_001211 | *BUB1B* | | | | *BUB1 mitotic checkpoint serine/threonine kinase B* | 2.30E-02 | -9.62 |
| NM_001267580 /// NM_003981 /// NM_199413 /// NM_199414 | *PRC1* | | | | *protein regulator of cytokinesis 1* | 4.11E-02 | -10.08 |
| NM_006845 | *KIF2C* | | | | *kinesin family member 2C* | 1.47E-02 | -10.16 |
| NM_001130851 /// NM_005192 | *CDKN3* | | | | *cyclin-dependent kinase inhibitor 3* | 2.78E-02 | -10.22 |
| NM_001166691 /// NM_003318 | *TTK* | | | | *TTK protein kinase* | 3.16E-02 | -10.35 |
| NM_020675 | *SPC25* | | | | *SPC25, NDC80 kinetochore complex component* | 1.69E-02 | -10.40 |
| NM_004336 | *BUB1* | | | | *BUB1 mitotic checkpoint serine/threonine kinase* | 5.37E-03 | -10.45 |
| NM_003600 /// NM_198433 /// NM_198434 /// NM_198435 /// NM_198436 /// NM_198437 | *AURKA* | | | | *aurora kinase A* | 2.68E-02 | -11.19 |
| NM_030919 | *FAM83D* | | | | *family with sequence similarity 83, member D* | 1.79E-02 | -11.57 |
| NM_006101 | *NDC80* | | | | *NDC80 kinetochore complex component* | 9.87E-03 | -11.78 |
| NM_001204182 /// NM_001204183 /// NM_002497 | *NEK2* | | | | *NIMA-related kinase 2* | 6.67E-03 | -11.83 |
| NM_001146015 /// NM_014750 | *DLGAP5* | | | | *discs, large (Drosophila) homolog-associated protein 5* | 2.21E-02 | -12.43 |
| NM_001237 | *CCNA2* | | | | *cyclin A2* | 2.13E-02 | -13.13 |
| NM_001206846 /// NM_018136 | *ASPM* | | | | *asp (abnormal spindle) homolog, microcephaly associated (Drosophila)* | 2.95E-02 | -13.81 |
| NM_001042426 /// NM_001809 /// NM_017877 | *CENPA /// SLC35F6* | | | | *centromere protein A /// solute carrier family 35, member F6* | 1.68E-02 | -14.48 |
| NM_001195228 /// NM_019013 | *FAM64A* | | | | *family with sequence similarity 64, member A* | 1.18E-02 | -15.60 |
| NM_005733 | *KIF20A* | | | | *kinesin family member 20A* | 6.72E-03 | -20.72 |
